# The burden of liver cirrhosis in mortality: Results from the global burden of disease study

**DOI:** 10.3389/fpubh.2022.909455

**Published:** 2022-08-11

**Authors:** Fei Ye, Mimi Zhai, Jianhai Long, Yi Gong, Chutong Ren, Dan Zhang, Xiang Lin, Sushun Liu

**Affiliations:** ^1^Department of General Surgery, The Second Xiangya Hospital, Central South University, Changsha, China; ^2^Xiangya Nursing School, Central South University, Changsha, China; ^3^Department of Respiratory, Beijing Tiantan Hospital, Capital Medicine University, Beijing, China; ^4^Clinical Nursing Teaching and Research Section, The Second Xiangya Hospital, Central South University, Changsha, China; ^5^Department of General Surgery, The Huaihua Second People's Hospital, Huaihua, China

**Keywords:** liver cirrhosis, global burden of disease study, mortality, ASR, EAPC

## Abstract

**Background:**

Liver cirrhosis-related death is a serious threat worldwide. The number of studies exploring the mortality trend of cirrhosis caused by specific etiologies was limited. This study aimed to demonstrate the pattern and trend based on the data of global burden of disease (GBD).

**Methods:**

The data of cirrhosis mortality were collected from the GBD 2017. The Age standardized mortality rate (ASR) and estimated annual percentage changes (EAPC) were used to estimate the temporal trend of liver cirrhosis mortality by etiologies, regions, sociodemographic index (SDI), and sexes.

**Results:**

Globally, mortality cases of cirrhosis increased by 47.15%. Although the global ASR of cirrhosis mortality remained stable during this period, the temporal trend varied in etiologies. The ASR of mortality caused by hepatitis C virus (HCV), alcohol consumption, and non-alcoholic steatohepatitis (NASH) increased with an EAPC of 0.17 (95% CI, 0.14–0.20), 0.20 (95% CI, 0.16–0.24), 1.00 (95% CI, 0.97–1.04), respectively. A decreasing trend of ASR was found among the causes of hepatitis B virus (BV) and other causes. The increased pattern was heterogeneous worldwide. The most pronounced increase trend was found in middle-high SDI regions and Eastern Europe. Contrarily, the most pronounced decrease trend was found in low SDI regions and Western Sub-Saharan Africa.

**Conclusion:**

Cirrhosis is still a public health problem. The growth trend of cirrhosis mortality caused by HCV was slowed by promoting direct-acting antiviral therapy. Unfortunately, we observed an unfavorable trend in etiologies for alcohol consumption and NASH, which indicated that more targeted and specific strategies should be established to limit alcohol consumption and promote healthy lifestyles in high-risk countries, especially in middle-high SDI regions and Eastern Europe.

## Introduction

Cirrhosis is the end stage of chronic liver disease (1). It has become one of the top 10 leading causes of death (2). More than 160 million people suffered from cirrhosis in 2017 around the world, and more than 0.8 million patients with cirrhosis died every year (3, 4). Among the etiologies, more than half of the patients were attributed to hepatitis B (HBV) and hepatitis C virus (HCV) (5, 6). Although the antiviral therapy has been continuously improved, the burden of cirrhosis caused by hepatitis is still quite large. Additionally, the decompensation rate and the survival of patients are also affected by the underlying etiologies (7).

Nowadays, the epidemiological distribution of cirrhosis etiologies varies worldwide due to socioeconomic development and antivirus therapy application. However, the detailed information was poorly described. Previous studies have indicated that HCV and alcohol were the major causes of cirrhosis in the United States, most European countries, and Japan. HBV caused cirrhosis mainly occurred in Asian-Pacific and African countries (8). As the morbidity and the mortality of cirrhosis are rising, it is important to study the epidemiological distribution of cirrhosis.

The Global Burden of Disease (GBD) is the most comprehensive worldwide observational epidemiological study. By exploring the trends from 1990 to 2017, we can find the challenges in health. Since Mokdad studied the burden of cirrhosis from 1980 to 2010 (6), there was no other study found in the database. Due to data additions, improvements, and methodological refinements made by GBD study 2017, it is now possible and timely to study the burden of cirrhosis in mortality and underlying etiologies. In the study, we discussed the mortality and the burden of cirrhosis worldwide from 1990 to 2017. For the first time, we demonstrated trends of cirrhosis mortality and provided estimates of annual sexual, geographical, and social liver cirrhosis rates for countries and territories.

## Methods

### Study data

In the study, the data of cirrhosis from 1990 to 2017 were collected from the Global Health Data Exchange (GHDx) query tool (http://ghdx.healthdata.org/gbd-results-tool) (9). The sociodemographic index (SDI) was used in our study. SDI used log lag-dependent income *per capita*, average educational attainment in the population over age 15, and the total fertility rate. Using SDI, the 195 countries and territories were divided into the low-SDI region, the low-middle-SDI region, the middle-SDI region, the high-middle-SDI region, and the high-SDI region. According to the geographical location, they were classified as 21 regions, including East Asia, Central Europe, North America-high income, etc. The analysis and estimated methods for studying the trend and burden of cirrhosis were mentioned by Liu et al. study (3). In brief, the “GBD compare,” a GBD visualization tool, was used to estimate the levels and trends of cirrhosis mortality. Additionally, human development index (HDI) was also used. It was a summary measure indicative of a long and healthy life, being knowledgeable and having a decent standard of living. In our study, HDI was collected and used in the study to match GBD data.

### Statistical analysis

The age-standardized mortality rate (ASR) and estimated annual percentage change (EAPC) were used to explore the trend of cirrhosis mortality (3). Using ASR, we can obtain the mortality of liver cirrhosis and changes in etiology. EAPC was also used to evaluate the changes of ASR during the period (10, 11). ASR increase was defined as a value of 95% CI of EAPC greater than 0. ASR decline was defined as a value of the 95% CI of EAPC <0. The ASR stability was defined as a value of the 95% CI of the EAPC containing 0. To explore the trend of ASR in etiologies, a complete linkage clustering in hierarchical clustering was used for EAPCs. Class spacing is defined as the maximum distance between two elements. In this complete linkage clustering, the two clusters with the smallest class spacing are combined preferentially, and the iteration ends when the result is unchanged. In our study, the end result was that 195 countries and territories were grouped into 4 categories, including significant increase, minor increase, remained stable or minor decrease, and significant decrease. By using hierarchy cluster analysis in the study, EAPC ≤ −1.34 was defined as a significant decrease group, −1.3 ≤ EAPC ≤ 0.92 was define as remained stable or a minor decrease group, 0.99 ≤ EAPC ≤ 3.38 was defined as a minor increase group, and EAPC≥ 3.87 was defined as a significant increase group. Additionally, a correlation analysis between EAPC and ASR–HDI was conducted to study the influential factors of EAPC. The R software (R 3.5.1, Institute for Statistics and Mathematics) and STATA/MP (STATA 13.1, StataCorp LLC) were used to analyze data. *p* <0.05 was considered to be statistically significant.

## Results

### Mortality burden of global cirrhosis

**Figure 1:**
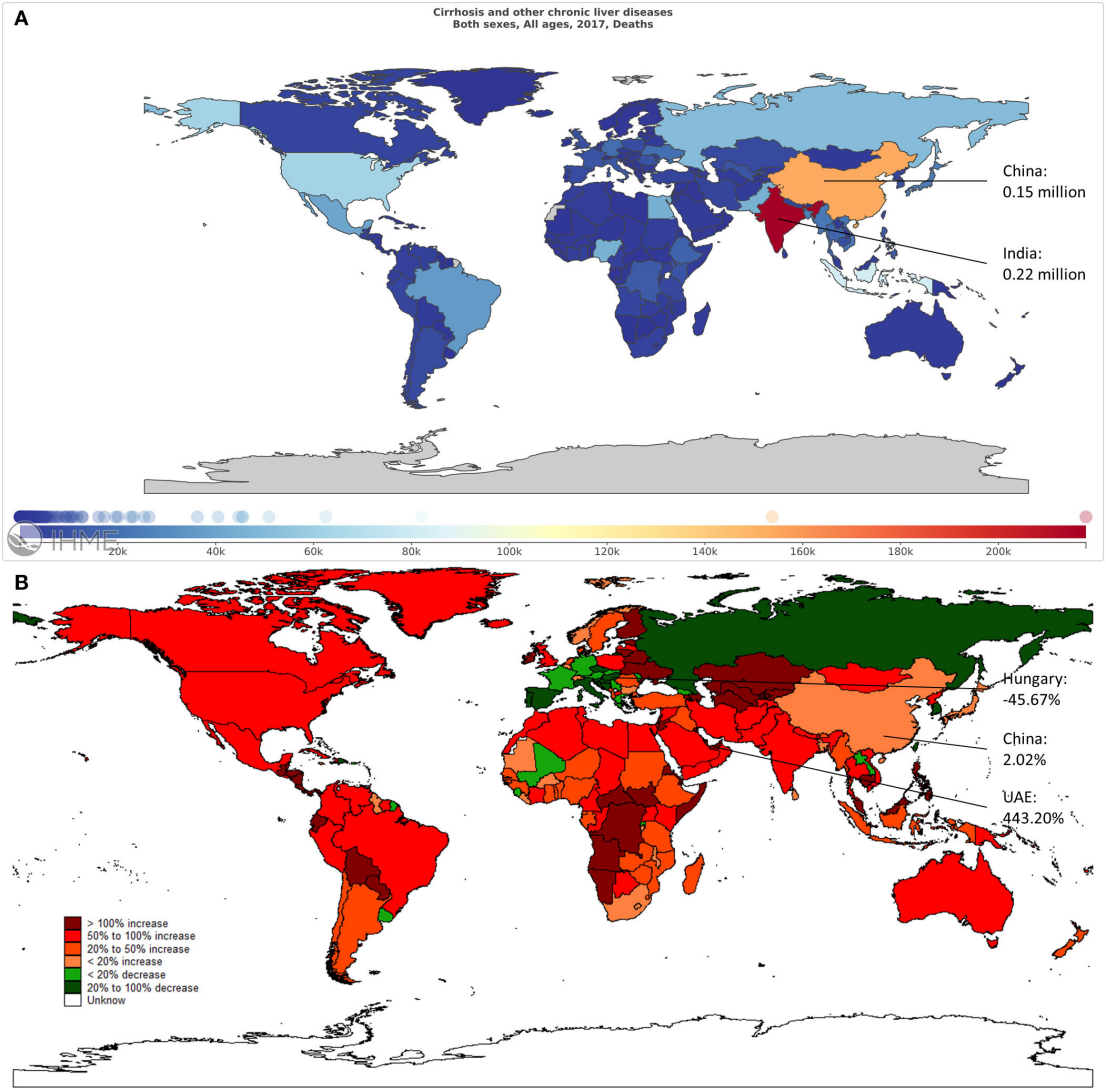
The overall mortality cases of liver cirrhosis in 195 countries and territories. **(A)** The mortality cases in 2017 across the world. **(B)** The change in mortality cases from 1990 to 2017 across the world.

Globally, the mortality of cirrhosis increased 47.15% from 1990 to 2017. India had the highest number of deaths in 2017 (Figure 1A). The highest growth of mortality was found in the United Arab Emirates (UAE), followed by Qatar and the Philippines. The mortality cases dropped the most in Hungary, with a decrease of 45.67%. During this period, the growth rate of mortality in China was 2.02% (Figure 1B).

**Figure 2:**
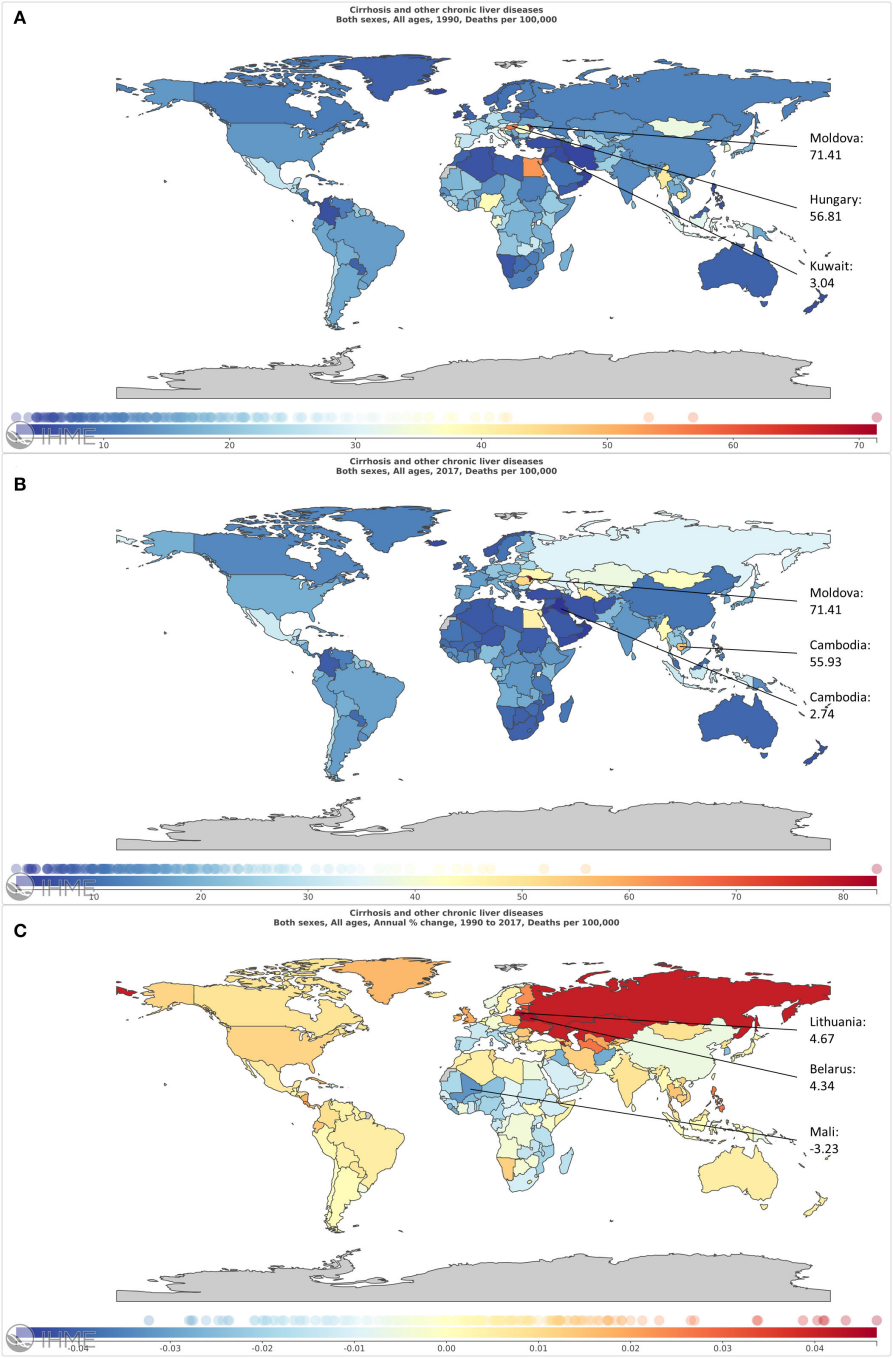
The global burden of liver cirrhosis mortality in 195 countries and territories. **(A,B)** The ASR of liver cirrhosis mortality in 1990 **(A)** and in 2017 **(B)**. **(C)** The ASR trend of liver cirrhosis from 1990 to 2017.

**Figure 3:**
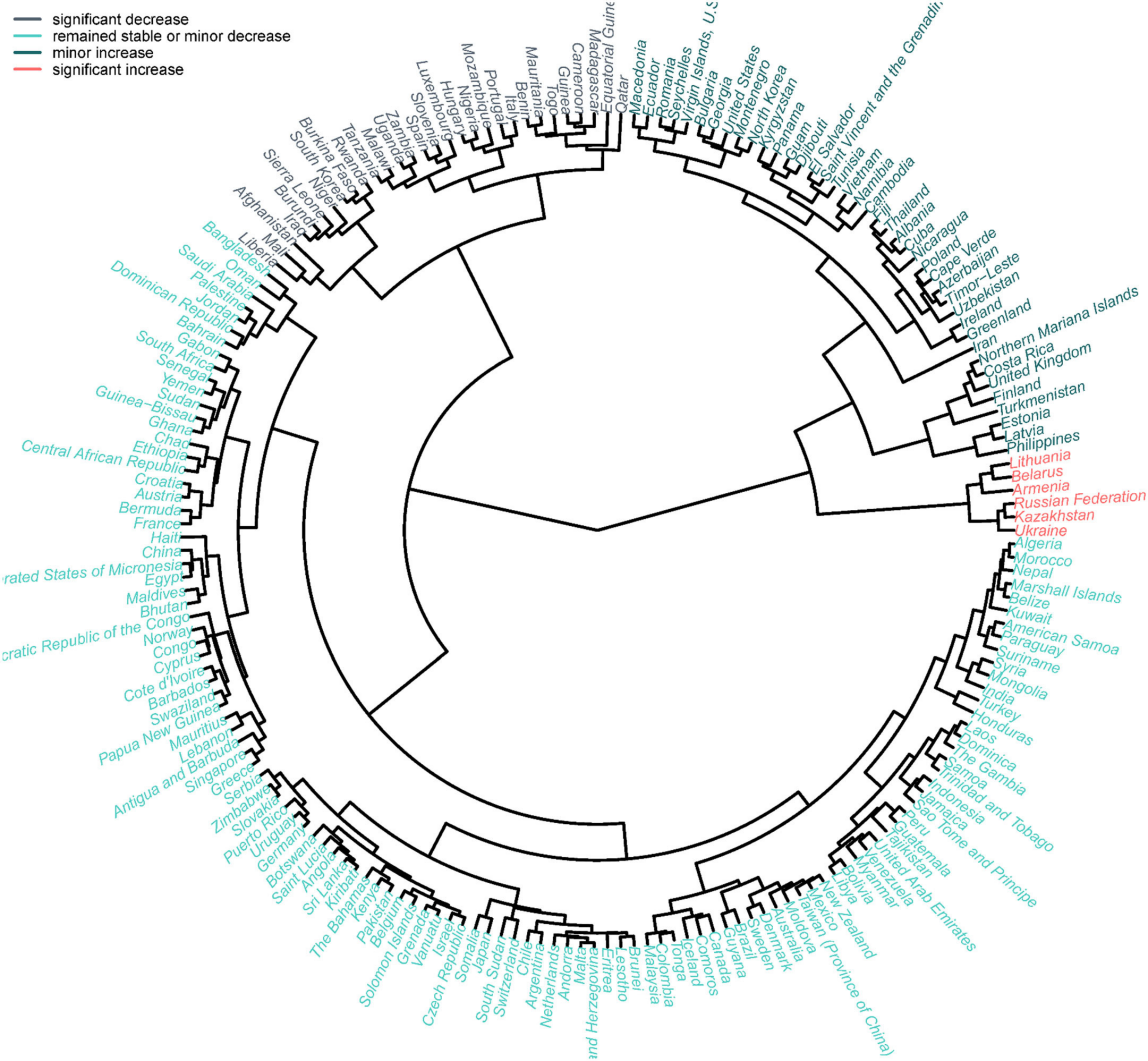
The temporal ASR trends clusters in etiologies related with liver cirrhosis for countries and territories.

The ASR of liver cirrhosis mortality varied over the world. It was 16.66 per 100,000 in 1990 and 17.31 per 100,000 in 2017 (Figures 2A,B, Table 1). In 1990, the ASR was higher in Western Sub-Saharan Africa and Central Europe, especially in Moldova (Figure 2A). In 2017, the ASR in Europe was higher (Figure 2B). Globally, the trend of ASR remained stable with an EAPC of 0.02 (95% CI, −0.01–0.06). Additionally, the ASR increased in male patients and decreased in female patients (Table 1). Higher EAPCs were found in European countries, including Lithuania, Belarus, and Russia. The lowest EAPC is in Mali (Figure 2C). Based on the results of cluster analysis, 6 countries (3.08%) were grouped into a significant increase group, such as Lithuania, Belarus, and Armenia. Forty-one countries (21.03%) were classified as the minor increase group. In contrast, 30 countries (15.38%) were classified as the significant decrease group, including South Korea, Spain, Italy, and Qatar. Additionally, most of the 195 countries (60.51%) were grouped into the remained stable or minor decrease group (Figure 3).

**Table 1:** The mortality cases, age-standardized mortality, and temporal trend of liver cirrhosis.

**Characteristics**	**1990**		**2017**		**1990–2017**	
	**Mortality cases No. ×10^3^ (95% UI)**	**ASR per 100,000 No. (95% UI)**	**Mortality cases No. ×10^3^ (95% UI)**	**ASR per 100,000 No. (95% UI)**	**EAPC No. (95% CI)**
Overall	898.99 (828.93–948.21)	16.66 (15.37–17.58)	1,322.87 (1,268.20–1,449.13)	17.31 (16.60–18.97)	0.02 (−0.01–0.06)
Sex					
Male	588.47 (532.79–625.28)	21.65 (19.61–23.01)	882.67 (838.34–966.51)	23.02 (21.86–25.21)	0.11 (0.08–0.15)
Female	310.52 (281.45–331.62)	11.60 (10.51–12.39)	440.20 (415.54–518.43)	11.57 (10.92–13.62)	−0.14 (−0.18– −0.09)
Socio-demographic index					
Low	111.18 (93.03–135.44)	15.94 (13.34–19.42)	171.16 (148.49–217.56)	13.27 (11.51–16.87)	−0.74 (−0.80– −0.67)
Low-middle	223.58 (187.05–249.36)	21.41 (17.91–23.88)	363.70 (329.01–404.22)	21.33 (19.30–23.71)	−0.11 (−0.16– −0.07)
Middle	240.42 (211.29–255.18)	15.50 (13.62–16.45)	366.92 (349.88–412.84)	17.55 (16.74–19.75)	0.23 (0.16–0.30)
Middle-high	144.08 (137.48–150.39)	12.96 (12.36–13.52)	215.78 (205.55–245.43)	15.55 (14.82–17.69)	0.56 (0.41–0.72)
High	176.52 (173.37–178.78)	18.27 (17.95–18.51)	201.83 (195.82–208.23)	17.71 (17.18–18.27)	−0.13 (−0.22– −0.04)
Etiology					
Hepatitis B	287.01 (251.68–318.06)	5.32 (4.67–5.90)	383.97 (349.07–441.67)	5.03 (4.57–5.78)	−0.35 (−0.41– −0.29)
Hepatitis C	225.27 (201.66–248.59)	4.18 (3.74–4.61)	342.24 (312.60–381.10)	4.48 (4.09–4.99)	0.17 (0.14–0.20)
Alcohol consumption	215.19 (194.90–234.59)	3.99 (3.61–4.35)	332.27 (303.00–373.28)	4.35 (3.97–4.89)	0.20 (0.16–0.24)
NASH	61.88 (55.40–67.98)	1.15 (1.03–1.26)	118.03 (108.62–128.58)	1.54 (1.42–1.68)	1.00 (0.97–1.04)
Other causes	109.64 (96.65–126.68)	2.03 (1.79–2.35)	146.36 (130.86–164.57)	1.92 (1.71–2.15)	−0.33 (−0.37– −0.28)
Region					
Asia Pacific–high income	37.97 (37.24–38.67)	21.88 (21.45–22.28)	35.08 (32.40–37.04)	18.75 (17.32–19.80)	−0.68 (−0.94– −0.42)
Central Asia	13.06 (12.78–13.48)	18.72 (18.32–19.32)	30.86 (28.66–32.91)	33.94 (31.52–36.19)	2.17 (1.87–2.48)
East Asia	161.53 (132.16–174.09)	12.83 (10.50–13.83)	167.64 (154.66–215.37)	11.28 (10.41–14.50)	−0.98 (−1.17– −0.78)
South Asia	159.40 (143.50–184.96)	14.38 (12.94–16.68)	295.61 (268.24–378.26)	16.58 (15.05–21.22)	0.47 (0.40–0.54)
Southeast Asia	110.99 (98.34–120.21)	23.78 (21.07–25.75)	176.32 (164.70–190.08)	26.70 (24.94–28.78)	0.35 (0.30–0.41)
Australasia	1.53 (1.47–1.58)	7.57 (7.24–7.79)	2.48 (2.23–2.74)	8.73 (7.87–9.67)	0.70 (0.41–0.99)
Caribbean	5.45 (5.00–5.98)	15.44 (14.15–16.94)	7.29 (6.49–8.62)	15.77 (14.03–18.63)	−0.23 (−0.44– −0.02)
Central Europe	30.08 (29.45–30.90)	24.24 (23.73–24.89)	30.99 (29.83–32.15)	26.99 (25.98–28.00)	0.15 (−0.10–0.41)
Eastern Europe	30.56 (29.06–31.58)	13.47 (12.80–13.92)	78.29 (75.81–80.58)	37.24 (36.07–38.33)	4.08 (3.41–4.75)
Western Europe	81.43 (80.03–82.97)	21.11 (20.75–21.51)	74.23 (70.80–78.16)	17.14 (16.35–18.05)	−0.98 (−1.04– −0.91)
Andean Latin America	6.02 (5.53–6.74)	15.70 (14.42–17.58)	11.23 (10.16–12.32)	18.28 (16.54–20.05)	0.58 (0.48–0.69)
Central Latin America	32.02 (31.38–32.52)	19.51 (19.12–19.81)	59.05 (56.63–62.09)	23.11 (22.16–24.30)	0.46 (0.37–0.55)
Southern Latin America	9.59 (9.33–9.88)	19.35 (18.84–19.94)	12.82 (11.69–14.02)	19.54 (17.81–21.37)	0.19 (0.04–0.33)
Tropical Latin America	22.46 (21.88–23.02)	14.63 (14.26–15.00)	36.94 (35.69–38.04)	16.89 (16.32–17.39)	0.31 (0.22–0.39)
North Africa and Middle East	48.99 (37.29–55.72)	14.37 (10.94–16.35)	77.39 (61.26–90.64)	12.89 (10.21–15.10)	−0.39 (−0.51– −0.27)
North America–high income	38.76 (37.54–39.46)	13.81 (13.37–14.06)	67.35 (65.13–69.62)	18.66 (18.05–19.29)	1.50 (1.25–1.76)
Oceania	0.94 (0.81–1.10)	14.55 (12.60–16.96)	1.73 (1.44–2.11)	13.77 (11.46–16.70)	−0.18 (−0.27– −0.10)
Central Sub-Saharan Africa	10.42 (7.74–14.09)	18.94 (14.06–25.61)	20.33 (15.74–26.35)	16.71 (12.94–21.66)	−0.52 (−0.62– −0.42)
Eastern Sub-Saharan Africa	38.73 (32.17–46.35)	20.22 (16.79–24.20)	57.85 (44.09–69.97)	14.71 (11.21–17.80)	−1.44(−1.57– −1.31)
Southern Sub-Saharan Africa	5.79 (4.63–6.51)	11.03 (8.82–12.41)	6.78 (5.63–7.91)	8.77 (7.28–10.23)	−1.34 (−1.93– −0.75)
Western Sub-Saharan Africa	53.27 (33.63–75.52)	27.71 (17.49–39.28)	72.60 (48.86–101.65)	16.73 (11.26–23.43)	−1.99 (−2.17– −1.81)

**Figure 4:**
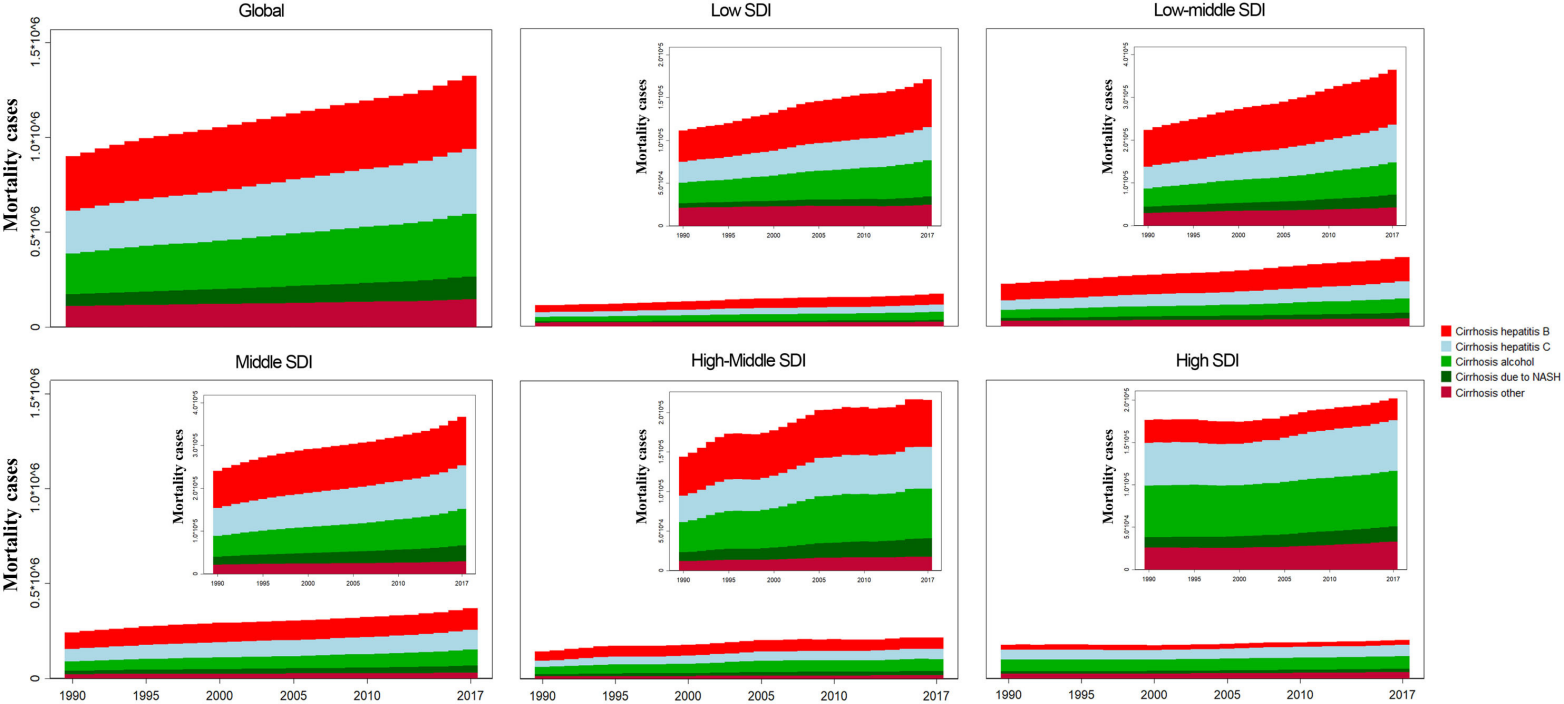
The liver cirrhosis mortality cases caused by different etiologies and SDI regions from 1990 to 2017.

**Figure 5:**
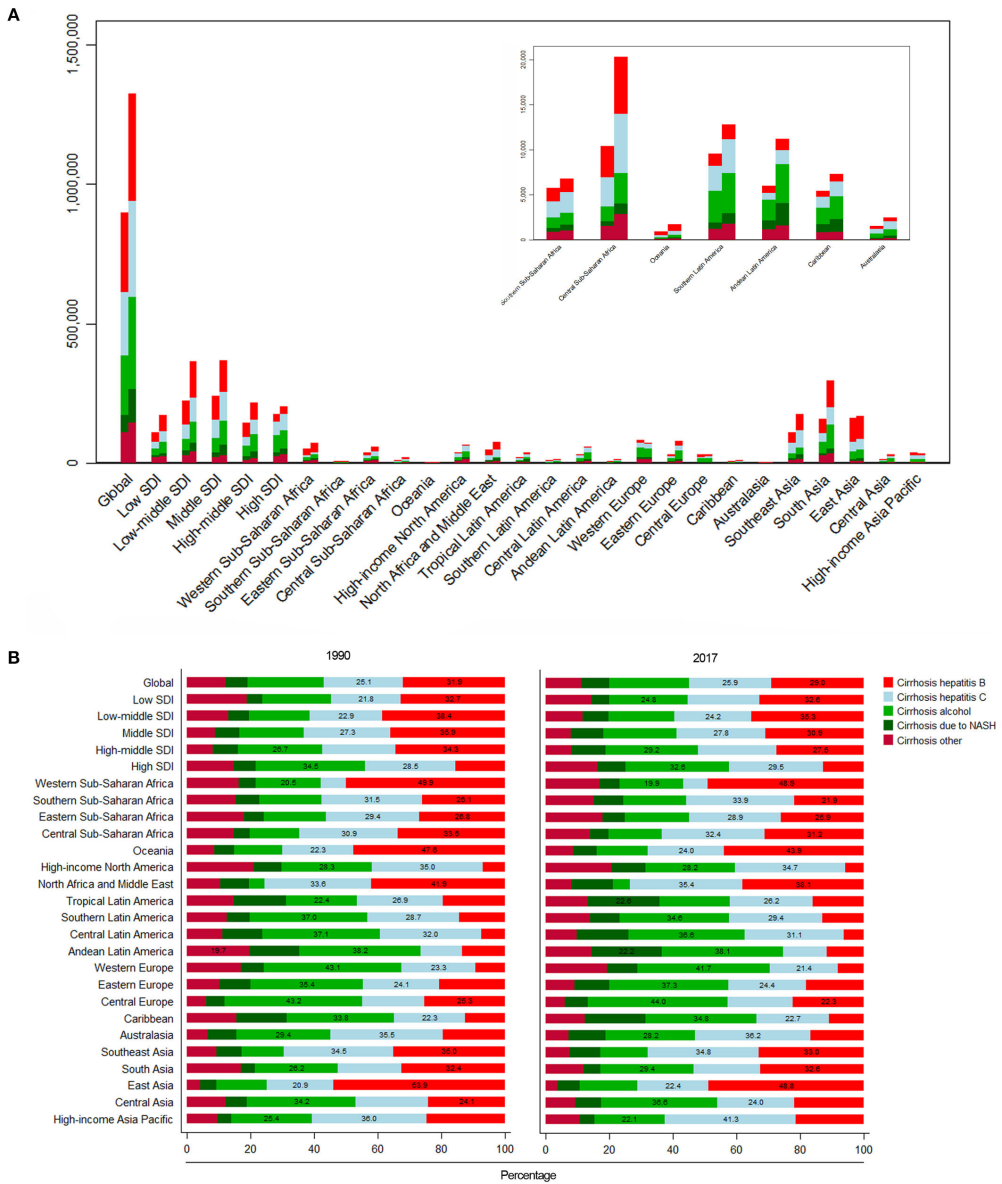
The liver cirrhosis mortality cases and its proportion in different countries and SDI regions by etiologies. **(A)** The mortality cases of liver cirrhosis in different SDI regions and geographical regions by different etiologies. **(B)** The proportion of liver cirrhosis mortality for different etiologies in different SDI regions and geographical regions.

**Figure 6:**
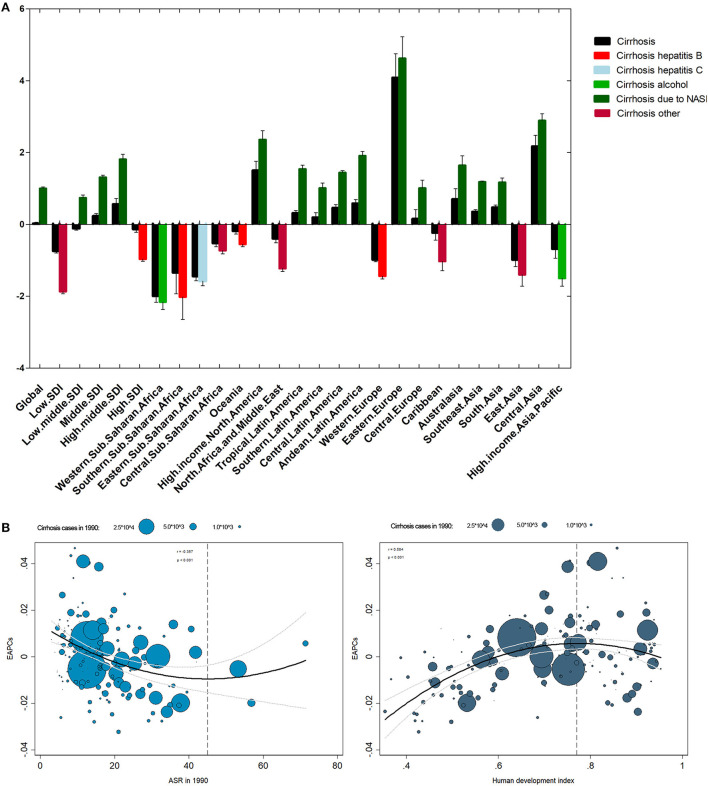
The EAPC of liver cirrhosis mortality at global and national levels. **(A)** The EAPC of liver cirrhosis mortality by regions and etiologies from 1990 to 2017. **(B)** The correlation between EAPC and ASR in 1990 and HDI in 2017.

For SDI regions, the mortality cases increased across the 5 SDI regions (Figure 4). However, the ASR decreased in the low, low-middle, and high-SDI regions (Table 1). For geographical regions, mortality cases increased in most of the regions except for Asia Pacific–high income and Western Europe (Figure 5A). During this period, the ASR in 11 of 21 regions demonstrated an increased trend, especially in Eastern Europe with the EAPC of 4.08 (95% CI, 3.41–4.75) (Figure 6A). In contrast, the most significant decrease of ASR was found in Western Sub-Saharan Africa (Figure 6A, Table 1).

For etiologies, HBV (29.03%) was the most important cause of cirrhosis in 2017, followed by HCV (25.87%) (Figure 5B). During the period, the proportion of HCV remained relatively stable. The proportion of HBV decreased from 31.93% in 1990 to 29.03% in 2017 (Figure 5B), but significant changes occurred in some regions. For instance, in the middle-high-SDI region, it decreased from 34.31 to 27.52%. Additionally, the proportion dropped from 41.93 to 38.14% in North Africa and the Middle East (Figure 5B).

### Influential factors for EAPC in mortality of liver cirrhosis

The ASR in 1990 demonstrated the disease reservoir at the baseline. The HDI in 2017 demonstrated the level of available medical resources. A significant negative association was found between EAPC and ASR in 1990 when the ASR was limited to below 45 per 100,000 (Figure 6B). On the contrary, the association disappeared when the ASR was >45 per 100,000 (Figure 6B). Additionally, EAPC was positively correlated with HDI in 2017 when HDI was < 0.77. The relationship between EAPC and HDI disappeared when HDI was >0.77 (Figure 6B).

### Mortality burden of liver cirrhosis due to HBV

Globally, ~29.03% of the mortality cases were caused by HBV in 2017 (Figure 5B). The proportion decreased from 31.93% in 1990 to 29.03% in 2017 (Figure 5B, Table 1). The highest mortality case was found in India (Supplementary Figure 1A). The mortality rate in the United Arab Emirates increased the most (Supplementary Figure 1B). The ASR caused by HBV was significantly heterogeneous across the world, and the highest ASR was observed in Southeast Asia (Supplementary Figures 2A,B, Supplementary Table 1). Contrary to the increasing trend of mortality cases, the ASR showed a downward trend with an EAPC of −0.35 (95% CI, −0.41– −0.29) (Supplementary Table 1). The highest EAPC was found in Lithuania, and the lowest EAPC was observed in Mali (Supplementary Figure 2C). The mortality cases increased in 4 SDI regions except for the high-SDI region, while the ASR decreased in all 5 SDI regions (Figure 4, Supplementary Figure 2C, Supplementary Table 1). For geographical regions, the mortality cases caused by HBV increased in 16 regions. In parallel, the greatest EAPC was found in Eastern Europe, and the lowest EAPC was found in Southern Sub-Saharan Africa and Western Sub-Saharan Africa (Supplementary Figure 2C, Supplementary Table 1).

### Mortality burden of liver cirrhosis due to HCV

In 2017, the mortality cases increased 51.92% from 1990 to 2017 (Supplementary Table 2, Supplementary Figure 3A). The proportion of mortality caused by HCV remained stable during the period (Figure 5). But the number of deaths increased dramatically in the United Arab Emirates (Supplementary Figure 3B). In China, mortality cases increased 9.20% during the period (Supplementary Figure 3B). The mortality caused by HCV was under 10 per 100,000 in most countries (Supplementary Figures 4A,B). The highest ASR was found in Moldova in 2017, followed by Cambodia and Egypt (Supplementary Figures 4A,B). The ASR was on the rise with the EAPC of 0.17 (95% CI, 0.14–0.20) (Supplementary Table 2). Among all countries, Lithuania had the highest EAPC, followed by Belarus and Armenia. Contrarily, the lowest EAPC was found in Mali (Supplementary Figure 4C). For SDI regions, the decrease trend of mortality caused by HCV was only observed in the low-SDI region with the EAPC of −0.65 (95% CI, −0.72– −0.59), and the most significant increase was found in the middle-high SDI region (Supplementary Table 2). Among the 21 geographical regions, 12 regions displayed an increase trend, and the most significant increase was found in Eastern Europe, followed by Central Asia. Contrarily, the most significant decrease was observed in Western Sub-Saharan Africa (Supplementary Table 2).

### Mortality burden of liver cirrhosis due to alcohol consumption

In 2017, ~25.12% of deaths caused by cirrhosis were ascribed to alcohol consumption (Supplementary Figure 5A, Table 1). The United Arab Emirates had the highest increase of mortality cases (434.87%), and China showed a 15.76% increase (Supplementary Figure 5B). The ASR minor increased from 1990 to 2017 (Supplementary Figures 6A,B, Supplementary Table 3). The higher ASR was found in Moldova, Romania, and Ukraine in 2017. The highest increase in ASR was found in Lithuania. Contrarily, the highest decrease in ASR was found in Mali (Supplementary Figure 6C). The ASR decreased in low- and high-SDI regions, but it increased in low-middle, middle, and middle-high regions (Supplementary Table 3). For geographical regions, ASR in nine regions was on the rise. Contrarily, 10 of 21 regions indicated a downward trend (Supplementary Figure 6C, Supplementary Table 3).

### Mortality burden of liver cirrhosis due to NASH

Globally, NASH precipitated nearly 8.92% of the total number of deaths from liver cirrhosis (Table 1). The proportion of NASH-induced deaths increased in all 5 SDI regions (Figure 5B). In 2017, India had the largest number of deaths (Supplementary Figure 7A). The mortality cases in the United Arab Emirates increased most significantly, while the mortality of China increased 39.87% during the same period (Supplementary Figure 7B). Hungary demonstrated the highest decrease (Supplementary Figure 7B). The ASR increased significantly with the EAPC of 1.00 (95% CI, 0.97–1.04) (Supplementary Figures 8A–C, Supplementary Table 4). The highest EAPC was found in Lithuania. In contrast, the most pronounced decrease in ASR was detected in Afghanistan (Supplementary Figure 8C). The ASR increased in 4 SDI regions, except for the low-SDI region (Supplementary Table 4). Moreover, the significantly increased ASR was found in the middle-high region with EAPC of 1.81 (95% CI, 1.66–1.95) (Supplementary Figure 8C, Supplementary Table 4). For geographical regions, most of the region (66.67%) showed an increase trend, especially in Eastern Europe. In contrast, Asia Pacific–high income and Sub-Saharan Africa demonstrated a decrease trend (Supplementary Table 4).

### Mortality burden of liver cirrhosis due to other causes

In 2017, deaths caused by other causes accounted for 11.06% (146.36 thousand) of total number of cirrhosis deaths (Supplementary Figure 9A, Supplementary Table 5). In North America–high income, the proportion exceeded 20%, albeit the ASR was at a relatively low level (Figure 5B, Supplementary Table 5). The mortality cases increased by 33.49% at the global level, and the most pronounced increase was observed in the United Arab Emirates (Supplementary Figure 9B). The global ASR decreased by an average 0.33% (95% CI, −0.37– −0.28). The highest ASR was observed in Moldova in 2017, while the highest increase of ASR was found in Armenia, followed by Lithuania (Supplementary Figures 10A–C). The ASR increased in middle-high, high-SDI regions and Australasia (Supplementary Figure 10C, Supplementary Table 5). Contrarily, a decrease trend of ASR was observed in low, low-middle, middle SDI regions and Western Sub-Saharan Africa (Supplementary Table 5).

## Discussion

Cirrhosis has been regarded as a major cause of the global health burden. The mortality caused by liver cirrhosis is gradually increasing (6). However, due to the heterogeneous pattern in risk factors of different countries, the mortality was obviously different (12). In the study, we demonstrated the key findings on cirrhosis mortality from GBD 2017. For the first time, we comprehensively estimated the trends of cirrhosis mortality for 195 countries and 5 SDI regions from 1990 to 2017. In general, mortality of cirrhosis increased by 47.15%. The trends were mainly dominated by an increase of HCV-caused cirrhosis, with a smaller contribution from alcohol use and NASH. Notably, the mortality cases induced by NASH increased 90.74%. Thus, the global health community should recognize the risk factors and pay more attention to the cirrhosis caused by alcohol consumption and NASH (6).

Cirrhosis caused by HBV was a major health burden worldwide. A previous study has indicated that about 248 million people were infected with HBV in 2010 (13). The mortality widely varied among countries. In some countries, HBV is the leading cause of cirrhosis, especially in East Asia and South Asia (13). Our study demonstrated that the ASR in Eastern Europe increased 3.69% per year. Contrarily, the ASR in Western Sub-Saharan Africa decreased 2.02% per year. This amazing variation might be partly explained by varying risk factors and transmission routes across countries. Additionally, our study showed that India and China had the highest number of deaths from HBV-related cirrhosis. However, mortality in India increased 102.6% from 1990 to 2017, while mortality in China decreased 7.6%. This might be related to the promoting HBV vaccine, screening of blood products, and obtaining safe injection methods in China. Moreover, socioeconomic development and improvement in people's cognition might also be related to the number of deaths. Unfortunately, despite the availability of effective vaccines and potent antiviral treatments, 76 countries out of 195 demonstrated an increased trend of ASR. This might be related to national HBV prevention policy, national immigration policy, national medical insurance policy, and national blood transfusion and blood donation policy (14, 15). Consequently, these countries should be advised to readjust their HBV prevention strategies (16). Additionally, ensuring blood safety and medical device safety were also important to reduce mortality (14, 17). Finally, the development of anti-HBV drugs might provide another way to reduce mortality of HBV-related cirrhosis (18).

Similar to previous studies, HCV was still the most important risk factor (19, 20). Compared with the cirrhosis mortality caused by HBV, the ASR of cirrhosis mortality caused by HCV increased in most regions. Although the prevalence remained low in most European countries and America, the ASR had a more significant increase in Eastern Europe, Central Asia, and North America–high income (21, 22). This might be due to the lack of effective vaccines. Moreover, a lack of treatment and poor efficacy were also closely related to the rising trend of mortality. Fortunately, direct-acting antiviral therapy has been on the market since 2014, and it was effective in more than 90% patients with HCV (23). Moreover, ensuring the safety of blood transfusion, injection drug use and therapeutic injections were also important for blocking HCV transmission (24). Now, the EAPC of HCV-related cirrhosis is significantly lower than other causes. Therefore, we predicted a possible downward trend in ASR of HCV-related liver cirrhosis.

The overall ASR of liver cirrhosis mortality caused by alcohol slightly increased, but decreased in low- and high-SDI regions. In our study, the increase was more significant in Eastern Europe, Central Asia, and North America–high income. This result was similar to a previous global survey (25). In our study, the ASR of cirrhosis mortality caused by alcohol decreased in Sub-Saharan Africa, although it increased in most regions. According to a previous global survey, the alcohol consumption was 17.1 liters per drinker in 2005, and the alcohol consumption was lowest in Africa (25). This result obtained by Liu et al. was consistent with our result (25). This might be related to the living habits, beliefs, and economic development of local people. Therefore, it might be necessary to formulate policies to limit alcohol consumption to reduce mortality and improve population-health outcomes. Moreover, we need to pay more attention to the drinking problem of young people and formulate relevant policies (26).

Although the mortality of cirrhosis caused by hepatitis decreased, the ever-increasing mortality of cirrhosis caused by NASH posed a continuing threat. In our study, the EAPC of cirrhosis mortality caused by NASH was highest among all etiologies. Moreover, the ASR increased in 4 SDI regions, and significantly increased in Asia. The increasing trend might be related to heavy and salty meat diet and westernized lifestyle (27). Similar results also demonstrated that a higher prevalence rate was found in China and other Asian countries in males and obese population (28). Based on a research, which indicated that the prevalence of NAFLD was higher in regions with a GDP <50,000 yuan and more than 100,000 yuan in China, the burden also might be closely related with national and personal economic levels (27). Thus, systemic treatment of metabolic diseases and loss of weight might effectively reduce the cirrhosis mortality caused by NASH. Additionally, we should closely monitor the patients suffering with cirrhosis caused by multiple causes, especially those who suffered with NASH and hepatitis (29).

In addition, our study showed that EAPC was negatively related with baseline ASR (<45/100,000), which indicated that countries with lower ASR had higher mortality of cirrhosis. This might be explained as follows. Firstly, the smaller the ASR, the more significant impact on the EAPC induced by ASR change. Secondly, countries with lower ASR might pay less attention to cirrhosis. Finally, with the focus on cirrhosis, EAPC increased with the increase of ASR, although there was no statistical significance. The HDI (< 0.77) was positively correlated with EAPC. This may be related to the improvement of living standards and medical technology; patients who were missed in the past had been diagnosed. As the HDI gradually increased, people became more aware of cirrhosis and invested more money and time in prevention and treatment, so the EAPC decreased.

## Conclusion

In summary, cirrhosis remains a huge threat to public health. Although the ASR of liver cirrhosis mortality caused by HBV decreased, the number of patients who died from cirrhosis due to HBV was high, especially in developing countries. The ASR of cirrhosis mortality caused by HCV still increased, although the direct-acting antiviral therapy for patients with HCV has been used since 2014. Additionally, cirrhosis due to alcohol consumption and NASH was a global health concern that cannot be ignored. Thus, developing policies to limit alcohol consumption and advocate healthy living were important to reduce the mortality, especially in several “high-risk” regions. By conducting this study, we can roughly illustrate the disease burden of cirrhosis mortality worldwide and formulate more reasonable and effective prevention strategies.

## Limitations

Although the GBD estimates demonstrated the burden of cirrhosis mortality, several limitations should be noted. First, some patients with cirrhosis were not included in the GBD database, and it might affect the results. Second, due to the data scarcity of GBD data, multi-etiological cirrhosis was not considered in this study. The interaction of several etiologies might play a role in promoting cirrhosis. For instance, alcohol consumption could worsen cirrhosis caused by hepatitis (30). Additionally, obesity and diabetes also increased the risk of cirrhosis caused by HCV (31). Therefore, further studies were needed to improve understanding of cirrhosis burden.

## Data availability statement

The original contributions presented in the study are included in the article/Supplementary material, further inquiries can be directed to the corresponding author.

## Author contributions

FY, MZ, and SL: study design. FY, JL, and SL: data collection. MZ, JL, and CR: data analysis. YG, XL, and DZ: figures. FY, MZ, and JL: manuscript writing. MZ and SL: manuscript proofing. All authors contributed to the article and approved the submitted version.

## Funding

This study was supported by Chen Xiaoping Science and Technology Development Foundation (CXPJJH11900001-2019334) and Hunan Provincial Natural Science Foundation of China (No. 2018JJ3715) in collection and analysis of the data.

## Conflict of interest

The authors declare that the research was conducted in the absence of any commercial or financial relationships that could be construed as a potential conflict of interest.

## Publisher's note

All claims expressed in this article are solely those of the authors and do not necessarily represent those of their affiliated organizations, or those of the publisher, the editors and the reviewers. Any product that may be evaluated in this article, or claim that may be made by its manufacturer, is not guaranteed or endorsed by the publisher.

## Supplementary material

Supplementary Figure 1 The overall mortality cases of liver cirrhosis caused by HBV in 195 countries and territories. **(A)** The mortality cases of liver cirrhosis caused by HBV in 2017 across the world. **(B)** The change in mortality cases of liver cirrhosis caused by HBV from 1990 to 2017 across the world.

Supplementary Figure 2 The global burden of liver cirrhosis mortality caused by HBV in 195 countries and territories. **(A,B)** The ASR of liver cirrhosis mortality caused by HBV in 1990 **(A)** and in 2017 **(B)**. **(C)** The ASR trend of liver cirrhosis caused by HBV from 1990 to 2017.

Supplementary Figure 3 The overall mortality cases of liver cirrhosis caused by HCV in 195 countries and territories. **(A)** The mortality cases of liver cirrhosis caused by HCV in 2017 across the world. **(B)** The change in mortality cases of liver cirrhosis caused by HCV from 1990 to 2017 across the world.

Supplementary Figure 4 The global burden of liver cirrhosis mortality caused by HCV in 195 countries and territories. **(A,B)** The ASR of liver cirrhosis mortality caused by HCV in 1990 **(A)** and in 2017 **(B)**. **(C)** The ASR trend of liver cirrhosis caused by HCV from 1990 to 2017.

Supplementary Figure 5 The overall mortality cases of liver cirrhosis caused by alcohol consumption in 195 countries and territories. **(A)** The mortality cases of liver cirrhosis caused by alcohol consumption in 2017 across the world. **(B)** The change in mortality cases of liver cirrhosis caused by alcohol consumption from 1990 to 2017 across the world.

Supplementary Figure 6 The global burden of liver cirrhosis mortality caused by alcohol consumption in 195 countries and territories. **(A,B)** The ASR of liver cirrhosis mortality caused by alcohol consumption in 1990 **(A)** and in 2017 **(B)**. **(C)** The ASR trend of liver cirrhosis caused by alcohol consumption from 1990 to 2017.

Supplementary Figure 7 The overall mortality cases of liver cirrhosis caused by NASH in 195 countries and territories. **(A)** The mortality cases of liver cirrhosis caused by NASH in 2017 across the world. **(B)** The change in mortality cases of liver cirrhosis caused by NASH from 1990 to 2017 across the world.

Supplementary Figure 8 The global burden of liver cirrhosis mortality caused by NASH in 195 countries and territories. **(A,B)** The ASR of liver cirrhosis mortality caused by NASH in 1990 **(A)** and in 2017 **(B)**. **(C)** The ASR trend of liver cirrhosis caused by NASH from 1990 to 2017.

Supplementary Figure 9 The overall mortality cases of liver cirrhosis caused by other causes in 195 countries and territories. **(A)** The mortality cases of liver cirrhosis caused by other causes in 2017 across the world. **(B)** The change in mortality cases of liver cirrhosis caused by other causes from 1990 to 2017 across the world.

Supplementary Figure 10 The global burden of liver cirrhosis mortality caused by other causes in 195 countries and territories. **(A,B)** The ASR of liver cirrhosis mortality caused by other causes in 1990 **(A)** and in 2017 **(B)**. **(C)** The ASR trend of liver cirrhosis caused by other causes from 1990 to 2017.

Supplementary Table 1 The mortality cases, age-standardized mortality, and temporal trend of liver cirrhosis caused by HBV.

Supplementary Table 2 The mortality cases, age-standardized mortality, and temporal trend of liver cirrhosis caused by HCV.

Supplementary Table 3 The mortality cases, age-standardized mortality, and temporal trend of liver cirrhosis caused by alcohol consumption.

Supplementary Table 4 The mortality cases, age-standardized mortality, and temporal trend of liver cirrhosis caused by NASH.

Supplementary Table 5 The mortality cases, age-standardized mortality, and temporal trend of liver cirrhosis caused by other causes.

Supplementary Table 6 The mortality cases and temporal trend of liver cirrhosis caused by each etiology in 195 countries and territories.

## Abbreviations

GBD: global burden of disease
HBV: hepatitis B virus
HCV: hepatitis C virus
SDI: sociodemographic index
HDI: human development index
ASR: age-standardized mortality rate
EAPC: estimated annual percentage changes
CI: confidence interval
NASH: non-alcoholic steatohepatitis.

## References

[1] Tsai TY Hung TH Livneh H Lin IH Lu MC Yeh CC. Chinese herbal medicine therapy and the risk of mortality for chronic hepatitis B patients with concurrent liver cirrhosis: a nationwide population-based cohort study. Oncotarget. (2018) 9:18214–23. 10.18632/oncotarget.24383 29719600 PMC5915067

[2] Corrao G Ferrari P Zambon A Torchio P Arico S Decarli A. Trends of liver cirrhosis mortality in Europe, 1970-1989: age-period-cohort analysis and changing alcohol consumption. Int J Epidemiol. (1997) 26:100–9. 10.1093/ije/26.1.100 9126509

[3] Liu Z Jiang Y Yuan H Fang Q Cai N Suo C. The trends in incidence of primary liver cancer caused by specific etiologies: results from the global burden of disease study 2016 and implications for liver cancer prevention. J Hepatol. (2019) 70:674–83. 10.1016/j.jhep.2018.12.001 30543829

[4] Nilsson E Anderson H Sargenti K Lindgren S Prytz H. Clinical course and mortality by etiology of liver cirrhosis in Sweden: a population based, long-term follow-up study of 1317 patients. Aliment Pharmacol Ther. (2019) 49:1421–30. 10.1111/apt.15255 30957910

[5] Blachier M Leleu H Peck-Radosavljevic M Valla DC Roudot-Thoraval F. The burden of liver disease in Europe: a review of available epidemiological data. J Hepatol. (2013) 58:593–608. 10.1016/j.jhep.2012.12.005 23419824

[6] Mokdad AA Lopez AD Shahraz S Lozano R Mokdad AH Stanaway J. Liver cirrhosis mortality in 187 countries between 1980 and 2010: a systematic analysis. BMC Med. (2014) 12:145. 10.1186/s12916-014-0145-y 25242656 PMC4169640

[7] Wiegand J Kuhne M Pradat P Mossner J Trepo C Tillmann HL. Different patterns of decompensation in patients with alcoholic vs. non-alcoholic liver cirrhosis. Aliment Pharmacol Ther. (2012) 35:1443–50. 10.1111/j.1365-2036.2012.05108.x 22530565

[8] Michitaka K Nishiguchi S Aoyagi Y Hiasa Y Tokumoto Y Onji M. Etiology of liver cirrhosis in Japan: a nationwide survey. J Gastroenterol. (2010) 45:86–94. 10.1007/s00535-009-0128-5 19789837

[9] Global Burden of Disease Collaborative Network. Global Burden of Disease Study 2017 Results. Seattle, United States: Institute for Health Metrics and Evaluation (IHME) (2017).

[10] Hung GY Horng JL Yen HJ Lee CY Lin LY. Changing incidence patterns of hepatocellular carcinoma among age groups in Taiwan. J Hepatol. (2015) 63:1390–6. 10.1016/j.jhep.2015.07.032 26256438

[11] Gao S Yang WS Bray F Va P Zhang W Gao J. Declining rates of hepatocellular carcinoma in urban Shanghai: incidence trends in 1976-2005. Eur J Epidemiol. (2012) 27:39–46. 10.1007/s10654-011-9636-8 22160277 PMC5477645

[12] Costa FO Lages EJP Lages EMB Cota LOM. Periodontitis in individuals with liver cirrhosis: a case-control study. J Clin Periodontol. (2019). 10.1111/jcpe.13172 31336404

[13] Schweitzer A Horn J Mikolajczyk RT Krause G Ott JJ. Estimations of worldwide prevalence of chronic hepatitis B virus infection: a systematic review of data published between 1965 and 2013. Lancet. (2015) 386:1546–55. 10.1016/S0140-6736(15)61412-X 26231459

[14] Lemoine M Thursz M Njie R Dusheiko G. Forgotten, not neglected: viral hepatitis in resource-limited settings, recall for action. Liver Int. (2014) 34:12–5. 10.1111/liv.12283 23998284

[15] Pollack HJ Kwon SC Wang SH Wyatt LC Trinh-Shevrin C Coalition A. Chronic hepatitis B and liver cancer risks among Asian immigrants in New York City: results from a large, community-based screening, evaluation, and treatment program. Cancer Epidemiol Biomarkers Prev. (2014) 23:2229–39. 10.1158/1055-9965.EPI-14-0491 25368398 PMC4373070

[16] Huang P Zhu LG Zhu YF Yue M Su J Zhu FC. Seroepidemiology of hepatitis B virus infection and impact of vaccination. World J Gastroenterol. (2015) 21:7842–50. 10.3748/wjg.v21.i25.7842 26167084 PMC4491971

[17] Lemoine M Eholie S Lacombe K. Reducing the neglected burden of viral hepatitis in Africa: strategies for a global approach. J Hepatol. (2015) 62:469–76. 10.1016/j.jhep.2014.10.008 25457207

[18] Choi J Lim YS. Comparison of risk of hepatocellular carcinoma between tenofovir and entecavir: One direction or no direction. J Hepatol. (2019). 10.1016/j.jhep.2019.06.013 31351769

[19] Perz JF Armstrong GL Farrington LA Hutin YJ Bell BP. The contributions of hepatitis B virus and hepatitis C virus infections to cirrhosis and primary liver cancer worldwide. J Hepatol. (2006) 45:529–38. 10.1016/j.jhep.2006.05.013 16879891

[20] Beste LA Leipertz SL Green PK Dominitz JA Ross D Ioannou GN. Trends in burden of cirrhosis and hepatocellular carcinoma by underlying liver disease in US veterans, 2001-2013. Gastroenterology. (2015) 149:1471–82 e5; quiz e17–8. 10.1053/j.gastro.2015.07.056 26255044

[21] Alter MJ Kruszon-Moran D Nainan OV McQuillan GM Gao F Moyer LA. The prevalence of hepatitis C virus infection in the United States, 1988 through 1994. N Engl J Med. (1999) 341:556–62. 10.1056/NEJM199908193410802 10451460

[22] Shepard CW Finelli L Alter MJ. Global epidemiology of hepatitis C virus infection. Lancet Infect Dis. (2005) 5:558–67. 10.1016/S1473-3099(05)70216-4 16122679

[23] Pawlotsky JM Feld JJ Zeuzem S Hoofnagle JH. From non-A, non-B hepatitis to hepatitis C virus cure. J Hepatol. (2015) 62:S87–99. 10.1016/j.jhep.2015.02.006 25920094

[24] Gower E Estes C Blach S Razavi-Shearer K Razavi H. Global epidemiology and genotype distribution of the hepatitis C virus infection. J Hepatol. (2014) 61:S45–57. 10.1016/j.jhep.2014.07.027 25086286

[25] Rehm J Shield KD. Global alcohol-attributable deaths from cancer, liver cirrhosis, and injury in 2010. Alcohol Res. (2013) 35:174–83. 24881325 10.35946/arcr.v35.2.07 PMC3908708

[26] Pimpin L Cortez-Pinto H Negro F Corbould E Lazarus JV Webber L. Burden of liver disease in Europe: epidemiology and analysis of risk factors to identify prevention policies. J Hepatol. (2018) 69:718–35. 10.1016/j.jhep.2018.05.011 29777749

[27] Zhou F Zhou J Wang W Zhang XJ Ji YX Zhang P. Unexpected rapid increase in the burden of NAFLD in China from 2008 to 2018: a systematic review and meta-analysis. Hepatology. (2019) 70:1119–33. 10.1002/hep.30702 31070259

[28] Younossi Z Anstee QM Marietti M Hardy T Henry L Eslam M. Global burden of NAFLD and NASH: trends, predictions, risk factors and prevention. Nat Rev Gastroenterol Hepatol. (2018) 15:11–20. 10.1038/nrgastro.2017.109 28930295

[29] Keating SE Hackett DA George J Johnson NA. Exercise and non-alcoholic fatty liver disease: a systematic review and meta-analysis. J Hepatol. (2012) 57:157–66. 10.1016/j.jhep.2012.02.023 22414768

[30] Lin CW Lin CC Mo LR Chang CY Perng DS Hsu CC. Heavy alcohol consumption increases the incidence of hepatocellular carcinoma in hepatitis B virus-related cirrhosis. J Hepatol. (2013) 58:730–5. 10.1016/j.jhep.2012.11.045 23220252

[31] Guariguata L Whiting DR Hambleton I Beagley J Linnenkamp U Shaw JE. Global estimates of diabetes prevalence for 2013 and projections for 2035. Diabetes Res Clin Pract. (2014) 103:137–49. 10.1016/j.diabres.2013.11.002 24630390

