# Supplementary material for: The burden of liver cirrhosis in mortality: Results from the global burden of disease study
Source: Front Public Health. 2022 Aug 11;10:909455. doi: 10.3389/fpubh.2022.909455 (PMC9403789; doi:10.3389/fpubh.2022.909455)
Supplement: Supplementary Figure 1 — The overall mortality cases of liver cirrhosis caused by HBV in 195 countries and territories. (A) The mortality cases of liver cirrhosis caused by HBV in 2017 across the world. (B) The change in mortality cases of liver cirrhosis caused by HBV from 1990 to 2017 across the world. [file Data_Sheet_1.PDF]

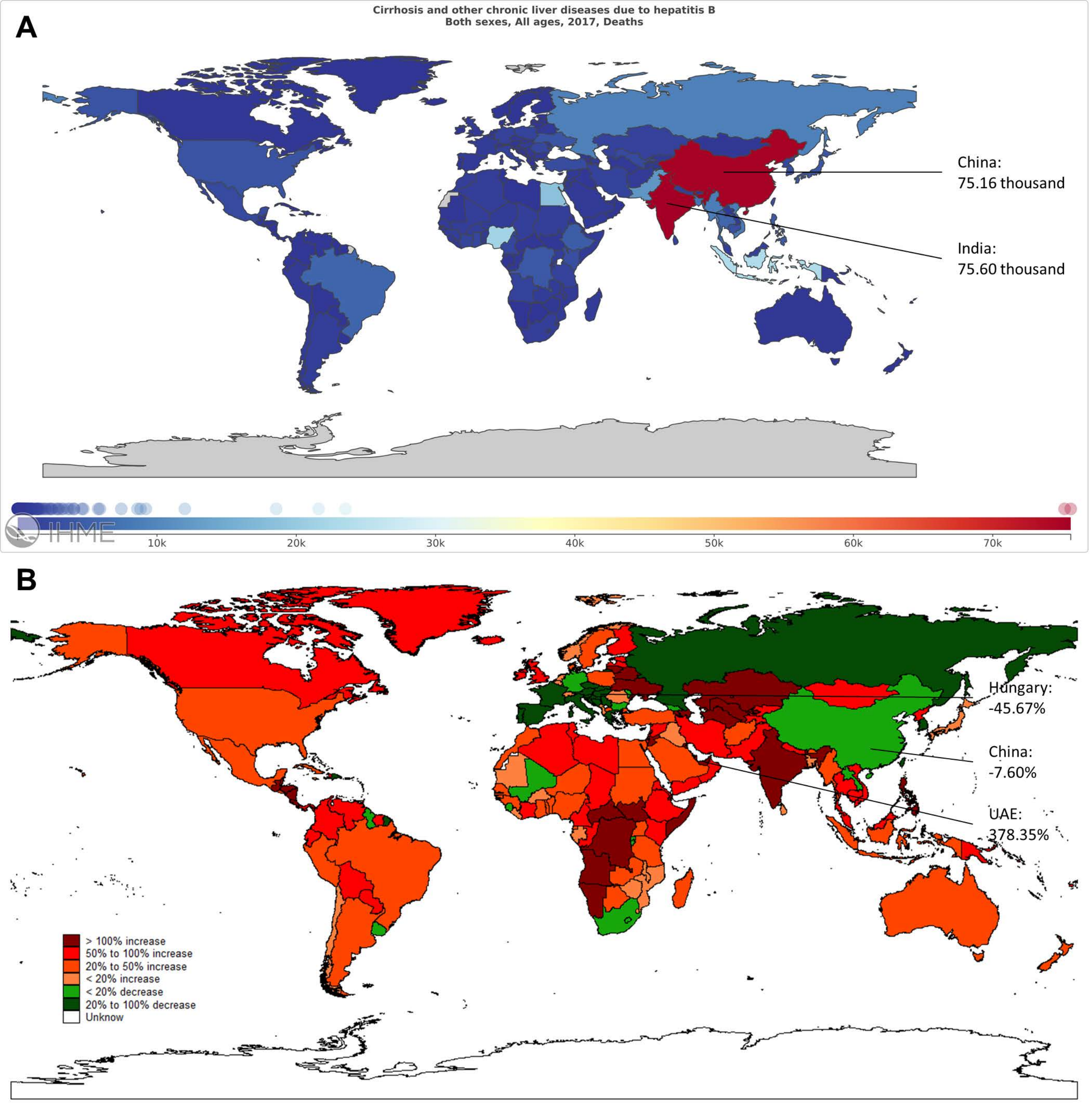
A
Cirrhosis and other chronic liver diseases due to hepatitis B
Both sexes, All ages, 2017, Deaths
China:
75.16 thousand
India:
75.60 thousand
IHME
10k
20k
30k
40k
50k
60k
70k
B
Hungary:
-45.67%
China:
-7.60%
UAE:
378.35%
> 100% increase
50% to 100% increase
20% to 50% increase
< 20% increase
< 20% decrease
20% to 100% decrease
Unknow

Fig S1

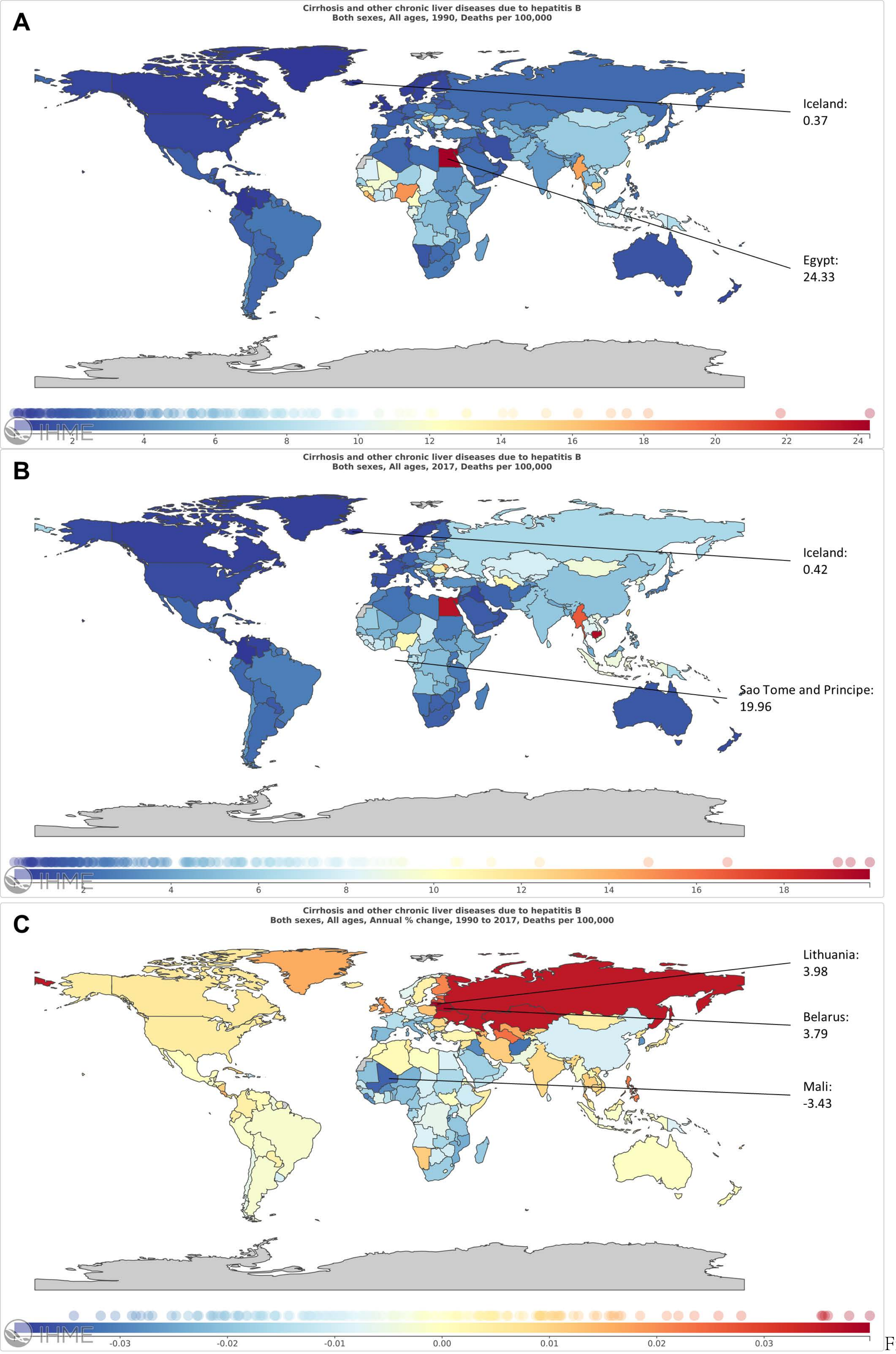
A
Cirrhosis and other chronic liver diseases due to hepatitis B
Both sexes, All ages, 1990, Deaths per 100,000
Iceland:
0.37
Egypt:
24.33
2 4 6 8 10 12 14 16 18 20 22 24
B
Cirrhosis and other chronic liver diseases due to hepatitis B
Both sexes, All ages, 2017, Deaths per 100,000
Iceland:
0.42
Sao Tome and Principe:
19.96
2 4 6 8 10 12 14 16 18
C
Cirrhosis and other chronic liver diseases due to hepatitis B
Both sexes, All ages, Annual % change, 1990 to 2017, Deaths per 100,000
Lithuania:
3.98
Belarus:
3.79
Mali:
-3.43
-0.03 -0.02 -0.01 0.00 0.01 0.02 0.03

Fig S2

A

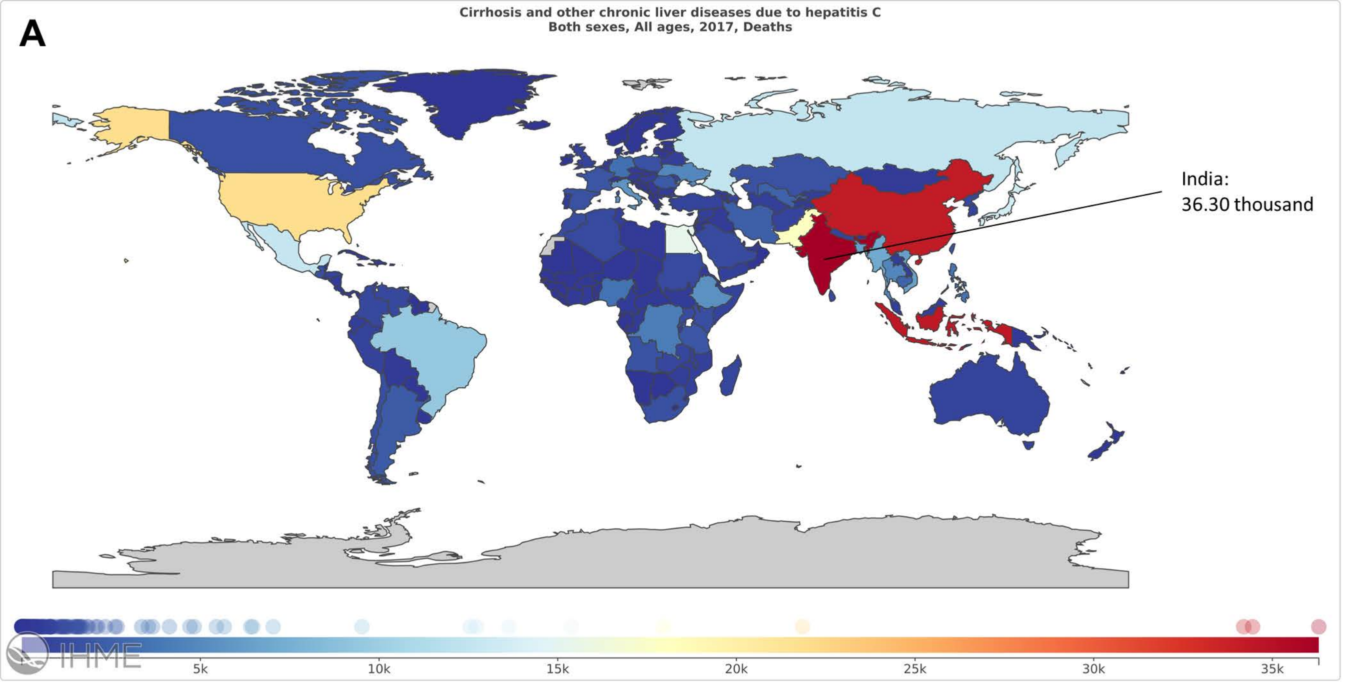
Cirrhosis and other chronic liver diseases due to hepatitis C
Both sexes, All ages, 2017, Deaths
India:
36.30 thousand
IHME
5k
10k
15k
20k
25k
30k
35k

B

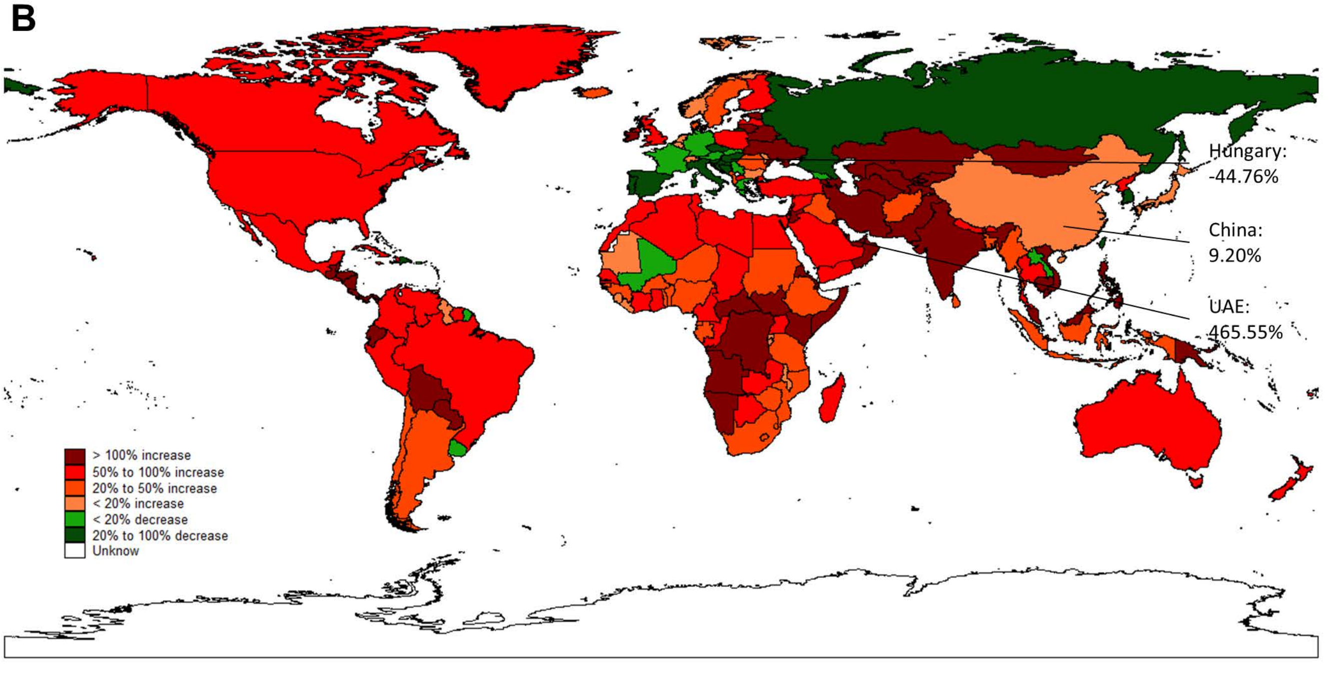
Hungary:
-44.76%
China:
9.20%
UAE:
465.55%
> 100% increase
50% to 100% increase
20% to 50% increase
< 20% increase
< 20% decrease
20% to 100% decrease
Unknow

Fig S3

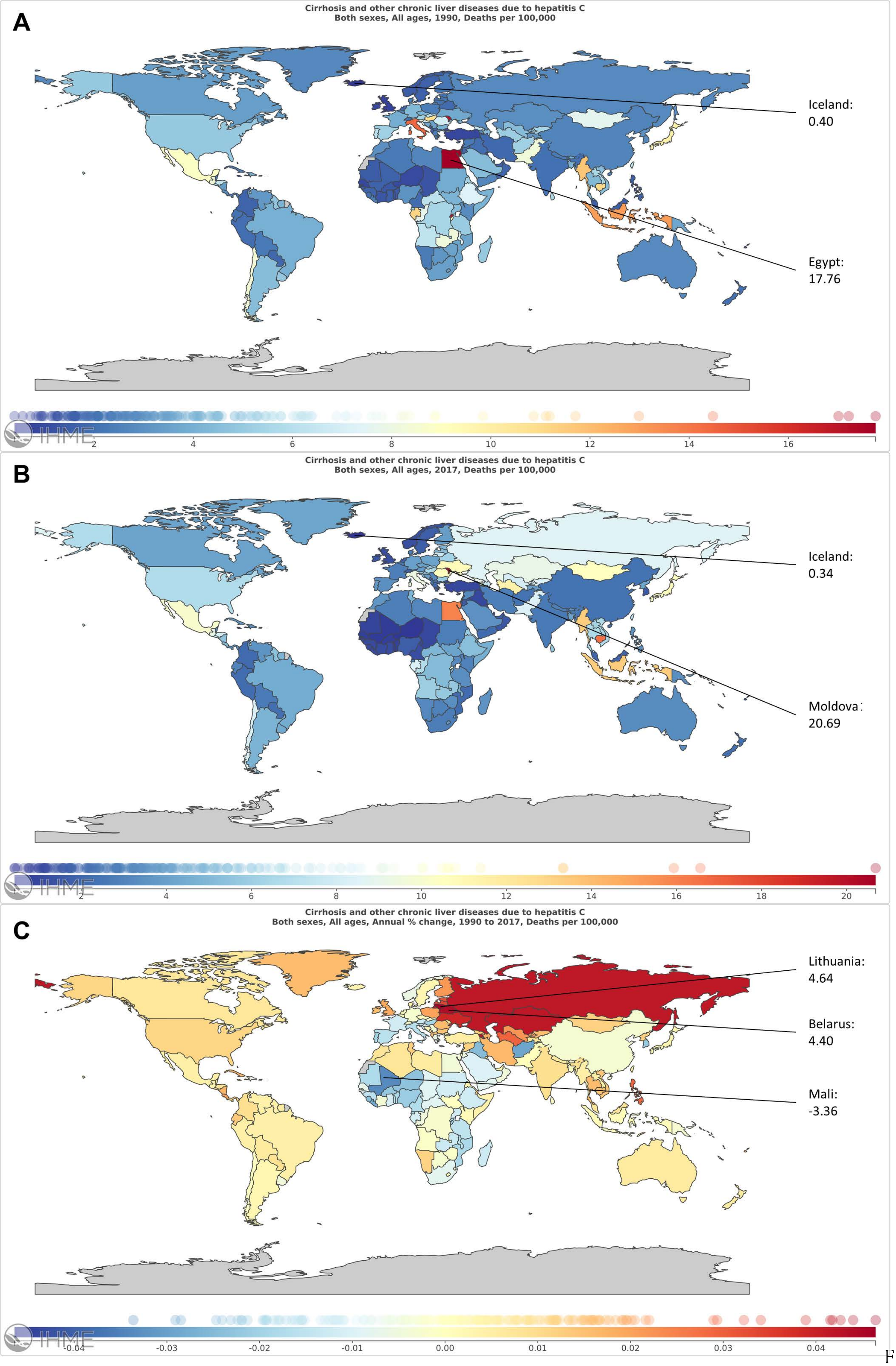
A
Cirrhosis and other chronic liver diseases due to hepatitis C
Both sexes, All ages, 1990, Deaths per 100,000
Iceland: 0.40
Egypt: 17.76
2 4 6 8 10 12 14 16
B
Cirrhosis and other chronic liver diseases due to hepatitis C
Both sexes, All ages, 2017, Deaths per 100,000
Iceland: 0.34
Moldova: 20.69
2 4 6 8 10 12 14 16 18 20
C
Cirrhosis and other chronic liver diseases due to hepatitis C
Both sexes, All ages, Annual % change, 1990 to 2017, Deaths per 100,000
Lithuania: 4.64
Belarus: 4.40
Mali: -3.36
-0.04 -0.03 -0.02 -0.01 0.00 0.01 0.02 0.03 0.04

Fig S4

A

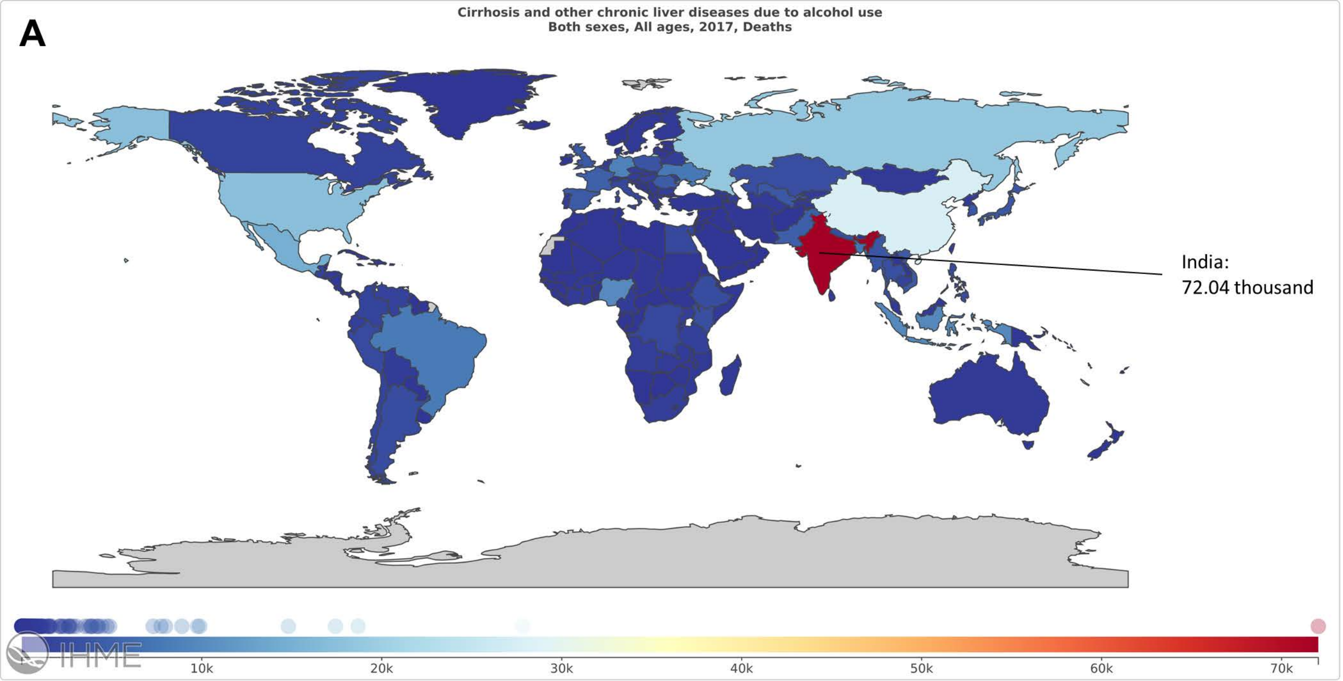
Cirrhosis and other chronic liver diseases due to alcohol use
Both sexes, All ages, 2017, Deaths
India:
72.04 thousand
IHME
10k
20k
30k
40k
50k
60k
70k

B

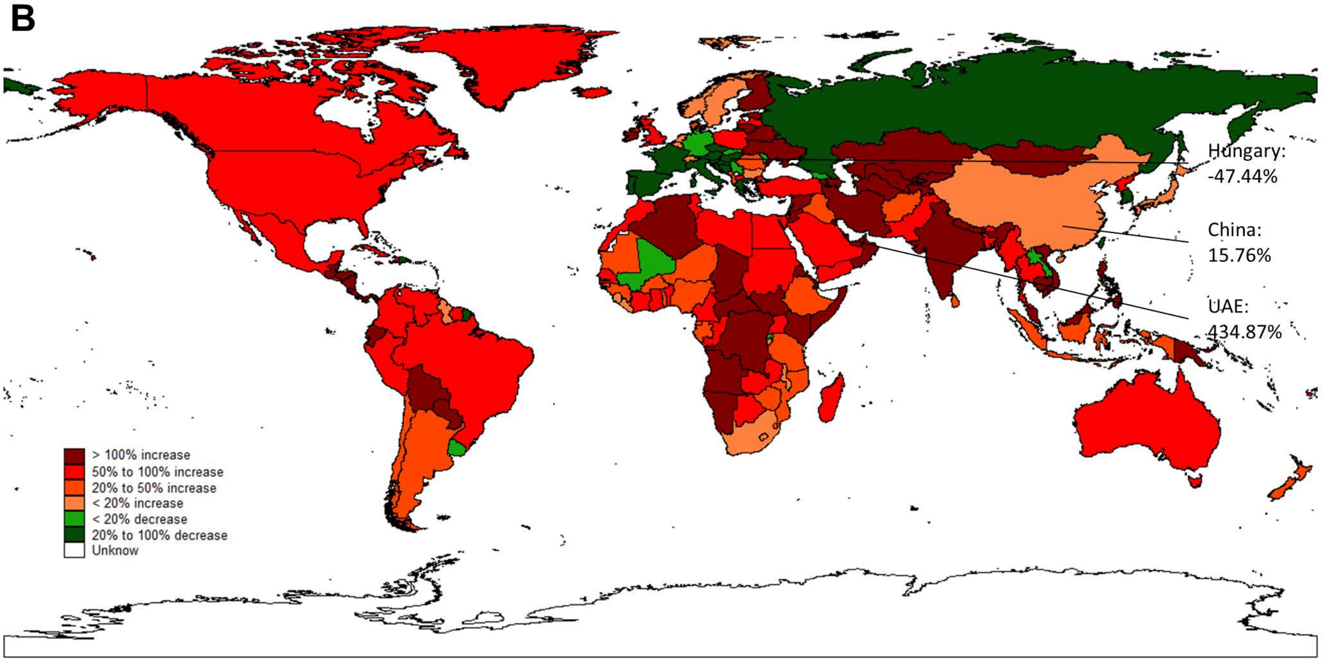
Hungary:
-47.44%
China:
15.76%
UAE:
434.87%
> 100% increase
50% to 100% increase
20% to 50% increase
< 20% increase
< 20% decrease
20% to 100% decrease
Unknow

Fig S5

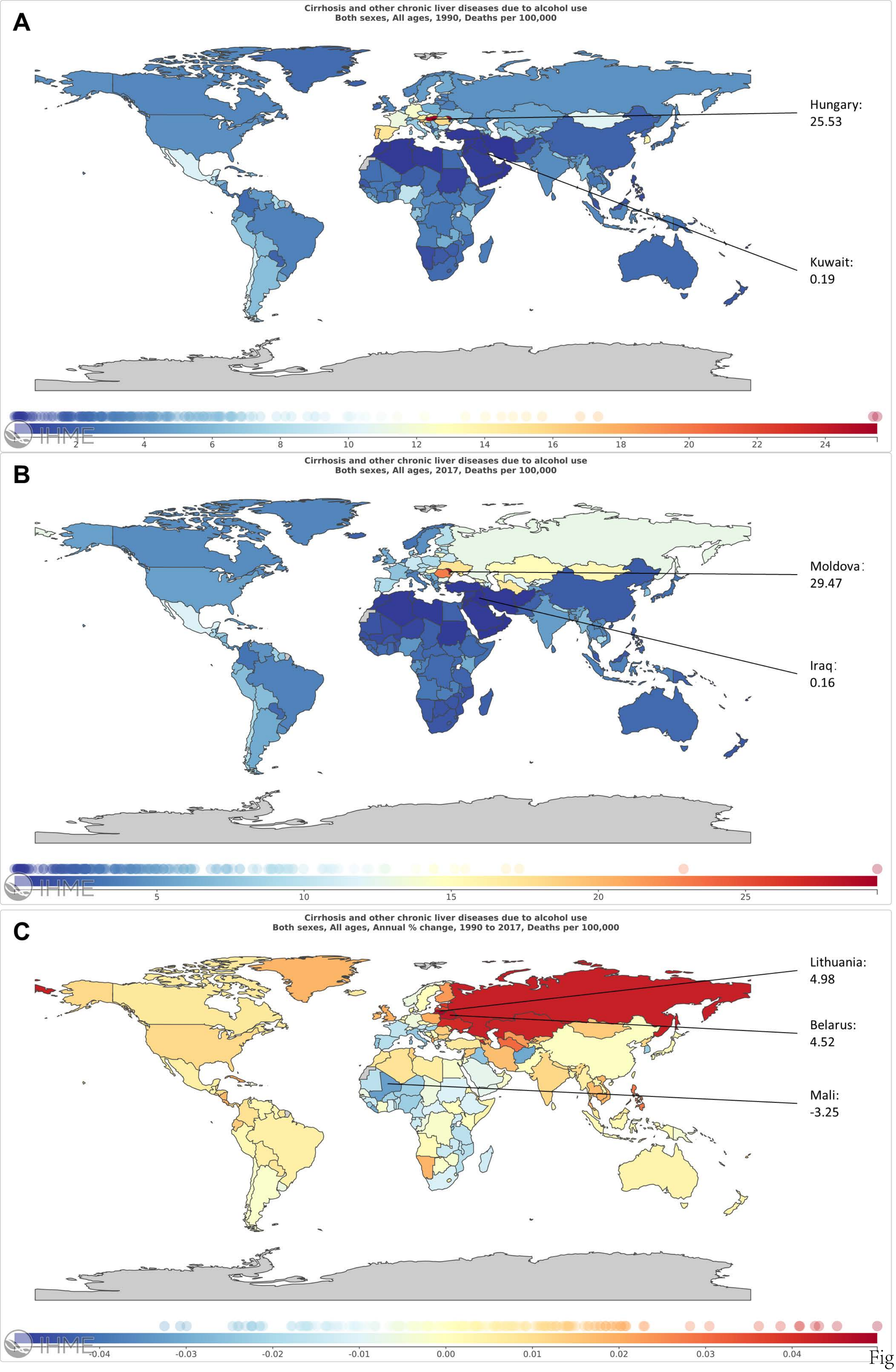
A
Cirrhosis and other chronic liver diseases due to alcohol use
Both sexes, All ages, 1990, Deaths per 100,000
Hungary:
25.53
Kuwait:
0.19
2 4 6 8 10 12 14 16 18 20 22 24
B
Cirrhosis and other chronic liver diseases due to alcohol use
Both sexes, All ages, 2017, Deaths per 100,000
Moldova:
29.47
Iraq:
0.16
5 10 15 20 25
C
Cirrhosis and other chronic liver diseases due to alcohol use
Both sexes, All ages, Annual % change, 1990 to 2017, Deaths per 100,000
Lithuania:
4.98
Belarus:
4.52
Mali:
-3.25
-0.04 -0.03 -0.02 -0.01 0.00 0.01 0.02 0.03 0.04

Fig S6

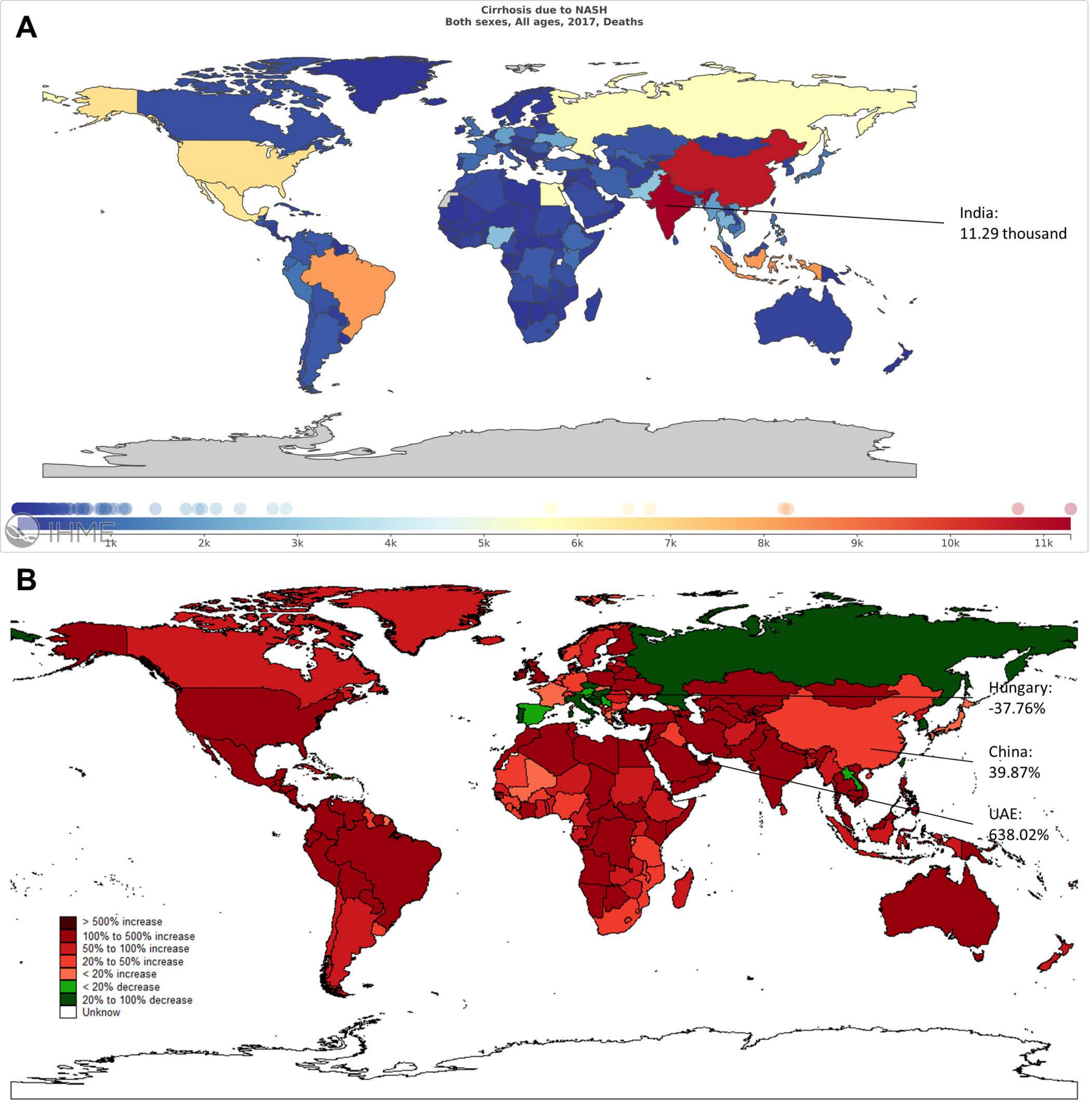
A
Cirrhosis due to NASH
Both sexes, All ages, 2017, Deaths
India:
11.29 thousand
1k
2k
3k
4k
5k
6k
7k
8k
9k
10k
11k
B
Hungary:
-37.76%
China:
39.87%
UAE:
638.02%
> 500% increase
100% to 500% increase
50% to 100% increase
20% to 50% increase
< 20% increase
< 20% decrease
20% to 100% decrease
Unknow

Fig S7

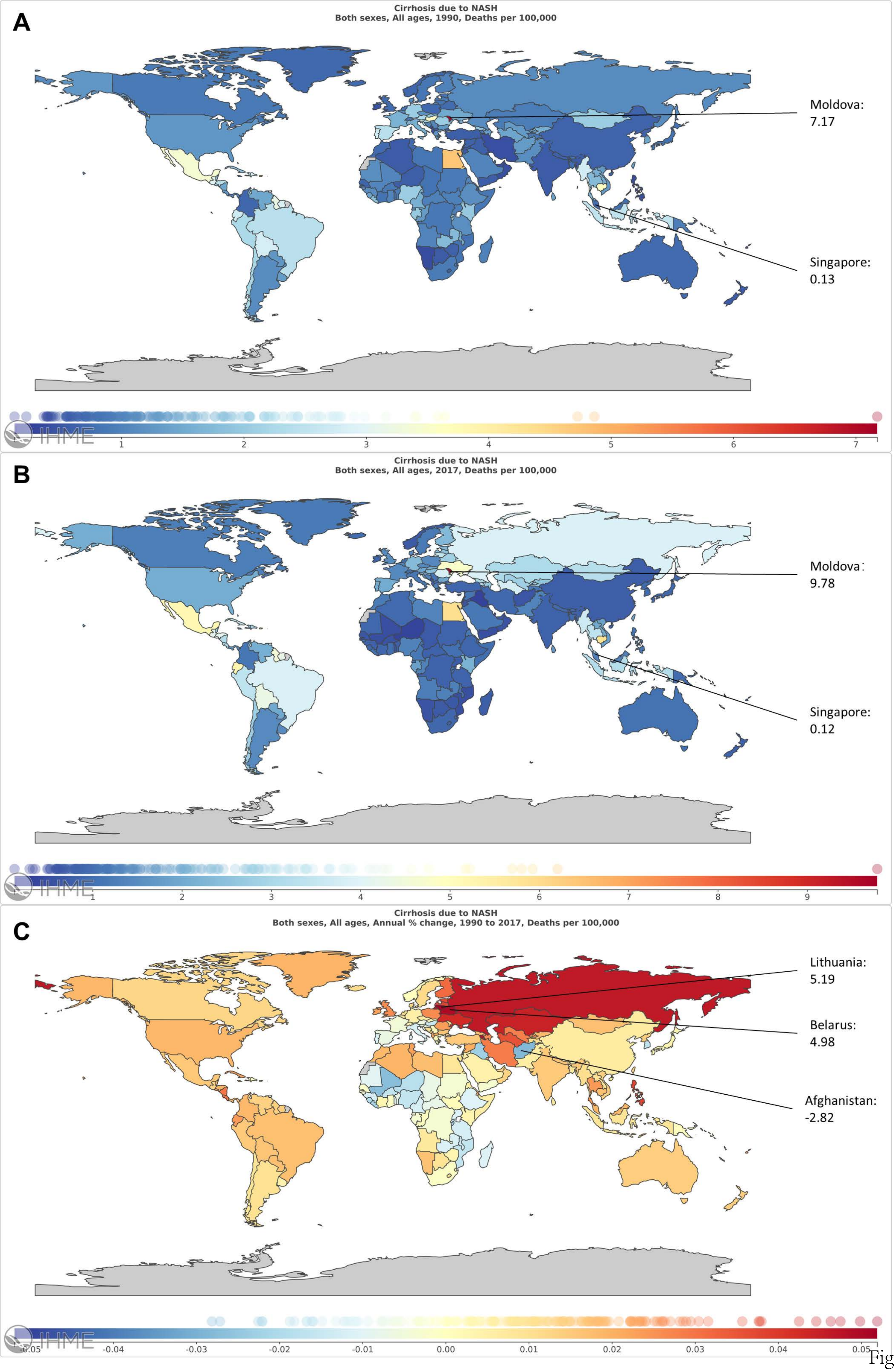
A
Cirrhosis due to NASH
Both sexes, All ages, 1990, Deaths per 100,000
Moldova:
7.17
Singapore:
0.13
IHME
1 2 3 4 5 6 7
B
Cirrhosis due to NASH
Both sexes, All ages, 2017, Deaths per 100,000
Moldova:
9.78
Singapore:
0.12
IHME
1 2 3 4 5 6 7 8 9
C
Cirrhosis due to NASH
Both sexes, All ages, Annual % change, 1990 to 2017, Deaths per 100,000
Lithuania:
5.19
Belarus:
4.98
Afghanistan:
-2.82
IHME
-0.05 -0.04 -0.03 -0.02 -0.01 0.00 0.01 0.02 0.03 0.04 0.05

Fig S8

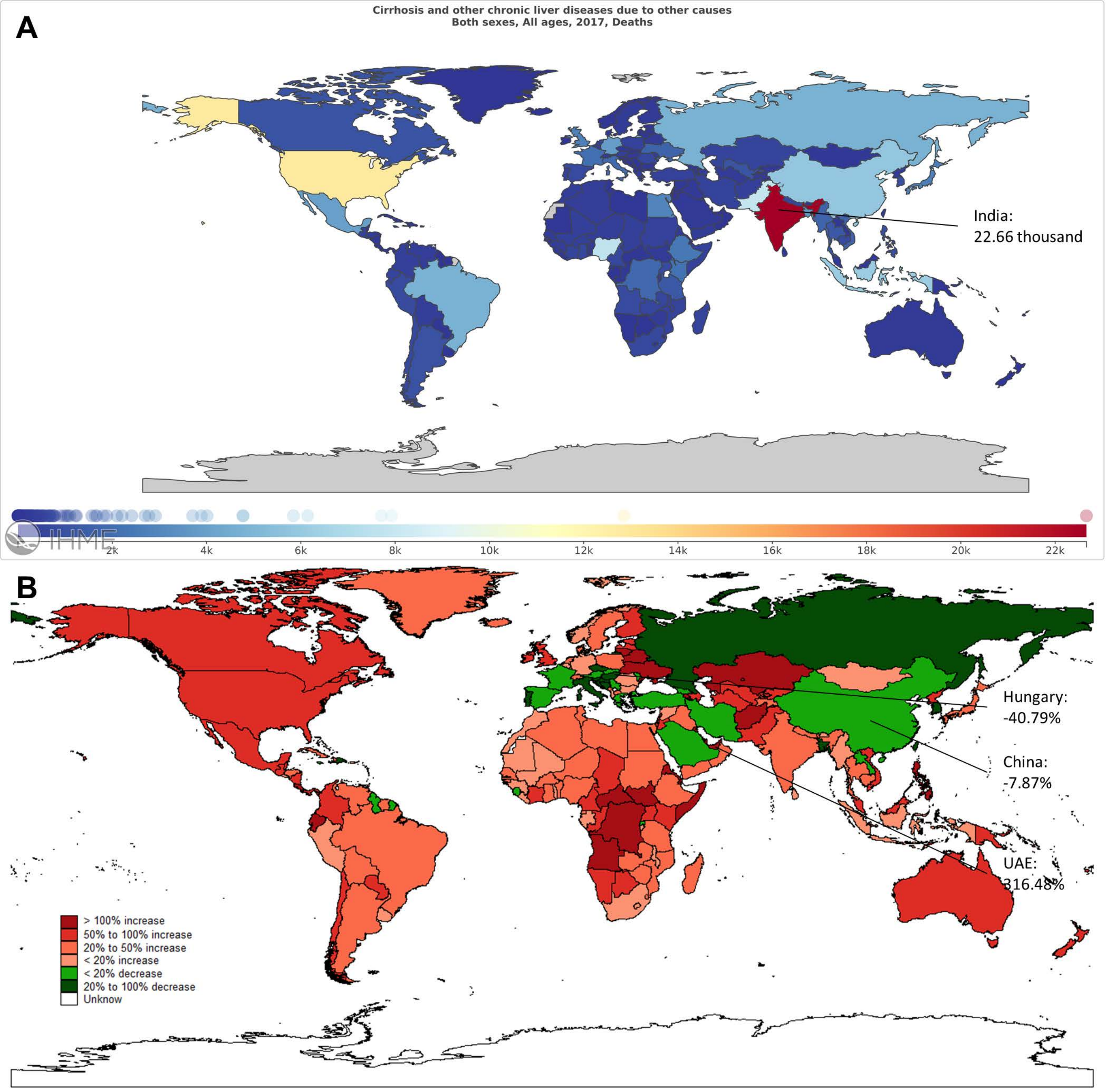
A
Cirrhosis and other chronic liver diseases due to other causes
Both sexes, All ages, 2017, Deaths
India:
22.66 thousand
2k
4k
6k
8k
10k
12k
14k
16k
18k
20k
22k
IHME
B
Hungary:
-40.79%
China:
-7.87%
UAE:
316.48%
> 100% increase
50% to 100% increase
20% to 50% increase
< 20% increase
< 20% decrease
20% to 100% decrease
Unknow

Fig S9

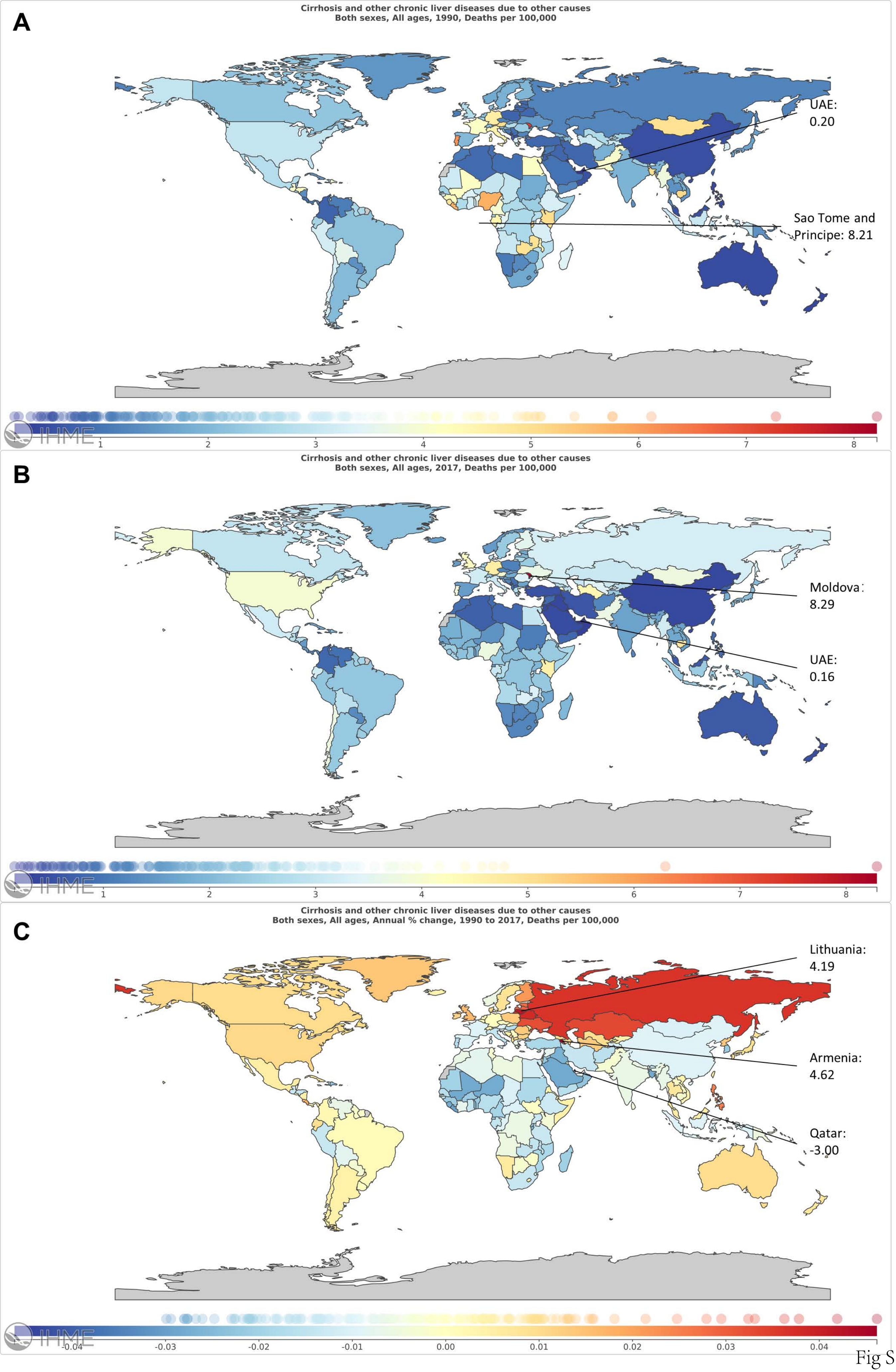

A
Cirrhosis and other chronic liver diseases due to other causes
Both sexes, All ages, 1990, Deaths per 100,000
UAE:
0.20
Sao Tome and
Principe: 8.21
IHME
1
2
3
4
5
6
7
8
B
Cirrhosis and other chronic liver diseases due to other causes
Both sexes, All ages, 2017, Deaths per 100,000
Moldova:
8.29
UAE:
0.16
IHME
1
2
3
4
5
6
7
8
C
Cirrhosis and other chronic liver diseases due to other causes
Both sexes, All ages, Annual % change, 1990 to 2017, Deaths per 100,000
Lithuania:
4.19
Armenia:
4.62
Qatar:
-3.00
IHME
-0.04
-0.03
-0.02
-0.01
0.00
0.01
0.02
0.03
0.04

Fig S10
