# Supplementary material for: The burden of liver cirrhosis in mortality: Results from the global burden of disease study
Source: Front Public Health. 2022 Aug 11;10:909455. doi: 10.3389/fpubh.2022.909455 (PMC9403789; doi:10.3389/fpubh.2022.909455)
Supplement: Supplementary Table 1 — The mortality cases, age-standardized mortality, and temporal trend of liver cirrhosis caused by HBV. [file Data_Sheet_2.docx]

**Supplemental Table 1. The mortality cases, age-standardized mortality, and temporal trend of liver cirrhosis caused by HBV**

| Characteristics | 1990 | |  | 2017 | |  | 1990–2017 |
| --- | --- | --- | --- | --- | --- | --- | --- |
|  | Mortality cases No. ×10^3^ (95% UI) | ASR per 100,000 No. (95% UI) |  | Mortality cases No. ×10^3^ (95% UI) | ASR per 100,000 No. (95% UI) |  | EAPC No. (95% CI) |
| Overall | 287.01(251.68-318.06) | 5.32(4.67-5.90) |  | 383.97(349.07-441.67) | 5.03(4.57-5.78) |  | -0.35(-0.41--0.29) |
| Sex |  |  |  |  |  |  |  |
| Male | 202.13(173.94-224.08) | 7.44(6.40-8.25) |  | 278.17(251.79-320.52) | 7.25(6.57-8.36) |  | -0.23(-0.30--0.15) |
| Female | 84.88(71.94-95.83) | 3.17(2.69-3.58) |  | 105.80(93.89-129.58) | 2.78(2.47-3.40) |  | -0.62(-0.67--0.57) |
| Socio-demographic index |  |  |  |  |  |  |  |
| Low | 36.37(30.07-44.09) | 5.21(4.31-6.32) |  | 55.83(46.81-72.28) | 4.33(3.63-5.60) |  | -0.68(-0.79--0.57) |
| Low-middle | 85.96(67.91-100.18) | 8.23(6.50-9.59) |  | 128.26(111.00-148.76) | 7.52(6.51-8.73) |  | -0.36(-0.41--0.32) |
| Middle | 86.42(72.39-95.02) | 5.57(4.67-6.13) |  | 113.26(102.57-132.74) | 5.42(4.91-6.35) |  | -0.36(-0.45--0.27) |
| Middle-high | 49.49(45.43-54.65) | 4.45(4.09-4.91) |  | 59.29(53.21-75.21) | 4.27(3.84-5.42) |  | -0.34(-0.48--0.20) |
| High | 27.19(24.64-29.81) | 2.82(2.55-3.09) |  | 25.83(23.18-28.60) | 2.27(2.03-2.51) |  | -0.97(-1.03--0.90) |
| Region |  |  |  |  |  |  |  |
| Asia Pacific–high income | 9.38(8.61-10.19) | 5.40(4.96-5.87) |  | 7.45(6.61-8.32) | 3.98(3.53-4.45) |  | -1.36(-1.62--1.09) |
| Central Asia | 3.15(2.77-3.58) | 4.51(3.97-5.13) |  | 6.76(5.86-7.73) | 7.43(6.45-8.51) |  | 1.84(1.42-2.27) |
| East Asia | 87.01(70.81-94.97) | 6.91(5.63-7.55) |  | 81.73(74.28-105.30) | 5.50(5.00-7.09) |  | -1.25(-1.41--1.09) |
| South Asia | 51.71(45.60-60.61) | 4.66(4.11-5.47) |  | 96.37(84.33-126.22) | 5.41(4.73-7.08) |  | 0.59(0.54-0.65) |
| Southeast Asia | 38.83(33.05-44.37) | 8.32(7.08-9.51) |  | 58.25(51.06-66.33) | 8.82(7.73-10.04) |  | 0.12(0.01-0.22) |
| Australasia | 0.30(0.27-0.33) | 1.47(1.32-1.63) |  | 0.41(0.36-0.48) | 1.46(1.25-1.68) |  | -0.03(-0.27-0.20) |
| Caribbean | 0.68(0.59-0.78) | 1.92(1.66-2.21) |  | 0.80(0.67-0.98) | 1.73(1.45-2.12) |  | -0.71(-0.92--0.50) |
| Central Europe | 7.61(6.83-8.50) | 6.13(5.50-6.85) |  | 6.92(6.13-7.77) | 6.03(5.34-6.77) |  | -0.42(-0.73--0.11) |
| Eastern Europe | 6.29(5.71-6.94) | 2.77(2.52-3.06) |  | 14.21(12.98-15.52) | 6.76(6.18-7.38) |  | 3.69(2.89-4.49) |
| Western Europe | 7.53(6.30-8.97) | 1.95(1.63-2.33) |  | 6.03(4.96-7.27) | 1.39(1.15-1.68) |  | -1.44(-1.52--1.37) |
| Andean Latin America | 0.81(0.70-0.94) | 2.11(1.83-2.45) |  | 1.31(1.13-1.53) | 2.13(1.84-2.49) |  | 0.01(-0.25-0.26) |
| Central Latin America | 2.34(2.17-2.51) | 1.43(1.32-1.53) |  | 3.72(3.39-4.06) | 1.46(1.33-1.59) |  | -0.17(-0.24--0.09) |
| Southern Latin America | 1.38(1.18-1.61) | 2.79(2.39-3.24) |  | 1.65(1.36-1.95) | 2.51(2.07-2.98) |  | -0.19(-0.25--0.12) |
| Tropical Latin America | 4.37(4.08-4.64) | 2.85(2.66-3.02) |  | 5.88(5.49-6.30) | 2.69(2.51-2.88) |  | -0.53(-0.64--0.43) |
| North Africa and Middle East | 20.54(14.33-25.08) | 6.03(4.20-7.36) |  | 29.52(22.51-37.06) | 4.92(3.75-6.18) |  | -0.81(-0.92--0.70) |
| North America–high income | 2.65(2.44-2.86) | 0.94(0.87-1.02) |  | 3.87(3.53-4.22) | 1.07(0.98-1.17) |  | 0.73(0.47-1.00) |
| Oceania | 0.45(0.38-0.52) | 6.93(5.95-8.09) |  | 0.76(0.62-0.94) | 6.04(4.96-7.43) |  | -0.55(-0.62--0.48) |
| Central Sub-Saharan Africa | 3.50(2.53-4.84) | 6.37(4.60-8.79) |  | 6.34(4.74-8.28) | 5.21(3.90-6.80) |  | -0.71(-0.78--0.65) |
| Eastern Sub-Saharan Africa | 10.40(8.24-13.11) | 5.43(4.30-6.84) |  | 14.98(11.33-18.74) | 3.81(2.88-4.77) |  | -1.45(-1.62--1.28) |
| Southern Sub-Saharan Africa | 1.51(1.19-1.74) | 2.88(2.26-3.32) |  | 1.49(1.19-1.80) | 1.92(1.54-2.32) |  | -2.02(-2.65--1.39) |
| Western Sub-Saharan Africa | 26.58(16.53-37.77) | 13.83(8.60-19.65) |  | 35.53(23.82-50.56) | 8.19(5.49-11.65) |  | -2.02(-2.21--1.82) |

**Supplemental Table 2.** **The mortality cases, age-standardized mortality, and temporal trend of liver cirrhosis caused by HCV**

| Characteristics | 1990 | |  | 2017 | |  | 1990–2017 |
| --- | --- | --- | --- | --- | --- | --- | --- |
|  | Mortality cases No. ×10^3^ (95% UI) | ASR per 100,000 No. (95% UI) |  | Mortality cases No. ×10^3^ (95% UI) | ASR per 100,000 No. (95% UI) |  | EAPC No. (95% CI) |
| Overall | 225.27(201.66-248.59) | 4.18(3.74-4.61) |  | 342.24(312.60-381.10) | 4.48(4.09-4.99) |  | 0.17(0.14-0.20) |
| Sex |  |  |  |  |  |  |  |
| Male | 145.23(128.97-161.38) | 5.34(4.75-5.94) |  | 224.66(202.93-250.52) | 5.86(5.29-6.53) |  | 0.26(0.23-0.29) |
| Female | 80.04(69.25-88.26) | 2.99(2.59-3.30) |  | 117.58(106.72-134.77) | 3.09(2.80-3.54) |  | 0.01(-0.04-0.07) |
| Socio-demographic index |  |  |  |  |  |  |  |
| Low | 24.29(19.58-29.49) | 3.48(2.81-4.23) |  | 38.73(32.43-47.94) | 3.00(2.51-3.72) |  | -0.65(-0.72--0.59) |
| Low-middle | 51.13(40.80-59.53) | 4.90(3.91-5.70) |  | 87.91(76.07-100.15) | 5.16(4.46-5.87) |  | 0.08(0.02-0.15) |
| Middle | 65.64(57.00-72.07) | 4.23(3.67-4.65) |  | 102.15(93.29-114.95) | 4.89(4.46-5.50) |  | 0.34(0.28-0.39) |
| Middle-high | 33.22(30.22-36.39) | 2.99(2.72-3.27) |  | 53.06(48.15-60.34) | 3.82(3.47-4.35) |  | 0.83(0.68-0.97) |
| High | 50.29(46.51-54.59) | 5.21(4.81-5.65) |  | 59.57(55.20-64.38) | 5.23(4.84-5.65) |  | 0.10(-0.04-0.25) |
| Region |  |  |  |  |  |  |  |
| Asia Pacific–high income | 13.67(13.11-14.19) | 7.88(7.55-8.18) |  | 14.49(13.38-15.40) | 7.75(7.15-8.24) |  | 0.09(-0.19-0.38) |
| Central Asia | 2.98(2.64-3.40) | 4.28(3.78-4.88) |  | 7.42(6.44-8.53) | 8.16(7.08-9.38) |  | 2.38(2.10-2.66) |
| East Asia | 33.75(27.53-37.63) | 2.68(2.19-2.99) |  | 37.56(33.35-48.49) | 2.53(2.24-3.26) |  | -0.79(-1.03--0.56) |
| South Asia | 31.89(28.29-36.26) | 2.88(2.55-3.27) |  | 61.62(54.06-76.04) | 3.46(3.03-4.27) |  | 0.62(0.54-0.69) |
| Southeast Asia | 38.27(32.94-43.41) | 8.20(7.06-9.30) |  | 61.41(54.04-69.39) | 9.30(8.18-10.51) |  | 0.40(0.35-0.46) |
| Australasia | 0.54(0.51-0.59) | 2.69(2.49-2.89) |  | 0.90(0.79-1.00) | 3.16(2.79-3.53) |  | 0.77(0.49-1.06) |
| Caribbean | 1.22(1.05-1.42) | 3.44(2.96-4.03) |  | 1.66(1.39-2.01) | 3.58(3.01-4.34) |  | -0.17(-0.37-0.03) |
| Central Europe | 5.88(5.21-6.62) | 4.73(4.20-5.34) |  | 6.33(5.56-7.18) | 5.51(4.84-6.26) |  | 0.36(0.09-0.63) |
| Eastern Europe | 7.36(6.70-8.01) | 3.24(2.95-3.53) |  | 19.12(17.56-20.69) | 9.09(8.35-9.84) |  | 4.09(3.45-4.74) |
| Western Europe | 19.00(16.05-22.49) | 4.93(4.16-5.83) |  | 15.87(13.08-19.10) | 3.67(3.02-4.41) |  | -1.30(-1.37--1.23) |
| Andean Latin America | 0.78(0.68-0.90) | 2.04(1.76-2.35) |  | 1.54(1.32-1.80) | 2.51(2.15-2.93) |  | 0.78(0.64-0.92) |
| Central Latin America | 10.24(9.77-10.72) | 6.24(5.95-6.53) |  | 18.38(17.31-19.62) | 7.20(6.77-7.68) |  | 0.37(0.30-0.45) |
| Southern Latin America | 2.75(2.40-3.12) | 5.56(4.85-6.31) |  | 3.77(3.22-4.35) | 5.74(4.91-6.63) |  | 0.25(0.12-0.39) |
| Tropical Latin America | 6.04(5.71-6.37) | 3.94(3.72-4.15) |  | 9.68(9.17-10.17) | 4.42(4.19-4.65) |  | 0.28(0.19-0.37) |
| North Africa and Middle East | 16.46(11.12-20.68) | 4.83(3.26-6.07) |  | 27.41(20.48-34.22) | 4.57(3.41-5.70) |  | -0.15(-0.27--0.02) |
| North America–high income | 13.55(12.87-14.14) | 4.83(4.59-5.04) |  | 23.35(22.04-24.59) | 6.47(6.11-6.81) |  | 1.48(1.24-1.72) |
| Oceania | 0.21(0.18-0.25) | 3.25(2.74-3.88) |  | 0.42(0.34-0.51) | 3.30(2.67-4.02) |  | 0.13(0.02-0.23) |
| Central Sub-Saharan Africa | 3.22(2.34-4.37) | 5.85(4.25-7.95) |  | 6.58(5.03-8.57) | 5.41(4.13-7.04) |  | -0.40(-0.51--0.29) |
| Eastern Sub-Saharan Africa | 11.40(8.80-14.37) | 5.95(4.59-7.50) |  | 16.74(12.40-21.23) | 4.26(3.15-5.40) |  | -1.58(-1.71--1.45) |
| Southern Sub-Saharan Africa | 1.82(1.43-2.09) | 3.47(2.72-3.97) |  | 2.30(1.88-2.72) | 2.97(2.43-3.52) |  | -1.07(-1.64--0.49) |
| Western Sub-Saharan Africa | 4.23(2.45-6.32) | 2.20(1.28-3.29) |  | 5.69(3.57-8.32) | 1.31(0.82-1.92) |  | -2.04(-2.18--1.91) |

**Supplemental Table 3. The mortality cases, age-standardized mortality, and temporal trend of liver cirrhosis caused by alcohol consumption**

| Characteristics | 1990 | |  | 2017 | |  | 1990–2017 |
| --- | --- | --- | --- | --- | --- | --- | --- |
|  | Mortality cases No. ×10^3^ (95% UI) | ASR per 100,000 No. (95% UI) |  | Mortality cases No. ×10^3^ (95% UI) | ASR per 100,000 No. (95% UI) |  | EAPC No. (95% CI) |
| Overall | 215.19(194.90-234.59) | 3.99(3.61-4.35) |  | 332.27(303.00-373.28) | 4.35(3.97-4.89) |  | 0.20(0.16-0.24) |
| Sex |  |  |  |  |  |  |  |
| Male | 154.60(138.54-169.67) | 5.69(5.10-6.24) |  | 241.38(219.49-267.78) | 6.29(5.72-6.98) |  | 0.26(0.23-0.30) |
| Female | 60.59(54.91-66.95) | 2.26(2.05-2.50) |  | 90.89(81.60-109.66) | 2.39(2.14-2.88) |  | 0.05(-0.01-0.10) |
| Socio-demographic index |  |  |  |  |  |  |  |
| Low | 24.09(19.74-29.26) | 3.45(2.83-4.20) |  | 42.45(35.76-56.09) | 3.29(2.77-4.35) |  | -0.20(-0.26--0.15) |
| Low-middle | 42.68(35.16-50.11) | 4.09(3.37-4.80) |  | 75.53(66.21-86.69) | 4.43(3.88-5.09) |  | 0.13(0.06-0.20) |
| Middle | 48.51(43.37-53.07) | 3.13(2.80-3.42) |  | 84.88(77.07-96.49) | 4.06(3.69-4.62) |  | 0.71(0.63-0.80) |
| Middle-high | 38.41(35.76-41.10) | 3.45(3.22-3.70) |  | 63.00(58.47-68.91) | 4.54(4.21-4.97) |  | 0.92(0.71-1.13) |
| High | 60.93(55.63-66.05) | 6.31(5.76-6.84) |  | 65.73(60.06-71.21) | 5.77(5.27-6.25) |  | -0.38(-0.44--0.33) |
| Region |  |  |  |  |  |  |  |
| Asia Pacific–high income | 9.65(8.79-10.43) | 5.56(5.07-6.01) |  | 7.75(6.85-8.55) | 4.15(3.66-4.57) |  | -1.50(-1.72--1.27) |
| Central Asia | 4.47(4.04-4.92) | 6.41(5.79-7.05) |  | 11.28(9.91-12.53) | 12.41(10.90-13.78) |  | 2.44(2.18-2.71) |
| East Asia | 25.81(20.85-28.55) | 2.05(1.66-2.27) |  | 30.31(27.06-39.33) | 2.04(1.82-2.65) |  | -0.63(-0.87--0.38) |
| South Asia | 41.71(35.58-49.68) | 3.76(3.21-4.48) |  | 86.77(75.29-114.81) | 4.87(4.22-6.44) |  | 0.86(0.76-0.97) |
| Southeast Asia | 14.77(12.08-17.67) | 3.17(2.59-3.79) |  | 26.28(21.82-31.23) | 3.98(3.30-4.73) |  | 0.81(0.76-0.87) |
| Australasia | 0.45(0.41-0.49) | 2.22(2.03-2.40) |  | 0.70(0.61-0.80) | 2.47(2.16-2.81) |  | 0.65(0.31-1.00) |
| Caribbean | 1.84(1.59-2.09) | 5.21(4.52-5.92) |  | 2.54(2.16-3.09) | 5.49(4.67-6.67) |  | -0.14(-0.35-0.06) |
| Central Europe | 13.00(12.18-13.92) | 10.48(9.81-11.21) |  | 13.63(12.56-14.66) | 11.87(10.94-12.77) |  | 0.23(-0.01-0.46) |
| Eastern Europe | 10.82(10.01-11.61) | 4.77(4.41-5.12) |  | 29.24(27.41-31.12) | 13.91(13.04-14.80) |  | 4.25(3.59-4.92) |
| Western Europe | 35.12(30.85-39.22) | 9.11(8.00-10.17) |  | 30.95(26.86-35.06) | 7.15(6.20-8.10) |  | -1.11(-1.17--1.05) |
| Andean Latin America | 2.30(2.05-2.64) | 5.99(5.33-6.87) |  | 4.28(3.74-4.84) | 6.97(6.09-7.87) |  | 0.55(0.42-0.68) |
| Central Latin America | 11.86(11.30-12.40) | 7.23(6.88-7.55) |  | 21.60(20.33-23.08) | 8.46(7.96-9.03) |  | 0.38(0.27-0.48) |
| Southern Latin America | 3.55(3.20-3.91) | 7.16(6.46-7.89) |  | 4.44(3.85-5.03) | 6.76(5.86-7.66) |  | -0.06(-0.27-0.16) |
| Tropical Latin America | 5.03(4.76-5.32) | 3.28(3.10-3.47) |  | 8.16(7.66-8.65) | 3.73(3.50-3.95) |  | 0.18(0.09-0.26) |
| North Africa and Middle East | 2.41(1.69-3.01) | 0.71(0.50-0.88) |  | 4.13(3.21-5.04) | 0.69(0.53-0.84) |  | -0.06(-0.18-0.07) |
| North America–high income | 10.98(10.40-11.51) | 3.91(3.71-4.10) |  | 18.97(17.90-20.10) | 5.26(4.96-5.57) |  | 1.52(1.27-1.77) |
| Oceania | 0.14(0.12-0.17) | 2.20(1.81-2.64) |  | 0.28(0.22-0.34) | 2.21(1.78-2.73) |  | 0.09(-0.03-0.21) |
| Central Sub-Saharan Africa | 1.64(1.18-2.22) | 2.98(2.15-4.04) |  | 3.41(2.58-4.46) | 2.80(2.12-3.67) |  | -0.30(-0.48--0.13) |
| Eastern Sub-Saharan Africa | 7.58(6.26-9.09) | 3.96(3.27-4.74) |  | 11.74(8.58-14.97) | 2.99(2.18-3.81) |  | -1.34(-1.47--1.22) |
| Southern Sub-Saharan Africa | 1.13(0.88-1.31) | 2.16(1.67-2.49) |  | 1.34(1.12-1.57) | 1.73(1.45-2.02) |  | -1.40(-1.98--0.82) |
| Western Sub-Saharan Africa | 10.92(6.15-16.26) | 5.68(3.20-8.46) |  | 14.46(8.90-20.86) | 3.33(2.05-4.81) |  | -2.16(-2.37--1.94) |

**Supplemental Table 4. The mortality cases, age-standardized mortality, and temporal trend of liver cirrhosis caused by NASH**

| Characteristics | 1990 | |  | 2017 | |  | 1990–2017 |
| --- | --- | --- | --- | --- | --- | --- | --- |
|  | Mortality cases No. ×10^3^ (95% UI) | ASR per 100,000 No. (95% UI) |  | Mortality cases No. ×10^3^ (95% UI) | ASR per 100,000 No. (95% UI) |  | EAPC No. (95% CI) |
| Overall | 61.88(55.40-67.98) | 1.15(1.03-1.26) |  | 118.03(108.62-128.58) | 1.54(1.42-1.68) |  | 1.00(0.97-1.04) |
| Sex |  |  |  |  |  |  |  |
| Male | 34.91(30.60-38.68) | 1.28(1.13-1.42) |  | 68.11(61.98-74.87) | 1.78(1.62-1.95) |  | 1.08(1.04-1.12) |
| Female | 26.97(23.76-29.72) | 1.01(0.89-1.11) |  | 49.92(45.64-55.86) | 1.31(1.20-1.47) |  | 0.91(0.87-0.95) |
| Socio-demographic index |  |  |  |  |  |  |  |
| Low | 5.39(4.47-6.48) | 0.77(0.64-0.93) |  | 9.63(7.96-12.22) | 0.75(0.62-0.95) |  | -0.24(-0.30--0.18) |
| Low-middle | 14.51(11.56-16.71) | 1.39(1.11-1.60) |  | 30.05(26.21-33.84) | 1.76(1.54-1.99) |  | 0.74(0.67-0.82) |
| Middle | 18.44(16.30-20.04) | 1.19(1.05-1.29) |  | 37.28(34.57-41.03) | 1.78(1.65-1.96) |  | 1.31(1.25-1.37) |
| Middle-high | 11.18(10.37-12.08) | 1.01(0.93-1.09) |  | 22.98(21.28-25.37) | 1.66(1.53-1.83) |  | 1.81(1.66-1.95) |
| High | 12.16(10.35-14.22) | 1.26(1.07-1.47) |  | 17.79(15.63-20.21) | 1.56(1.37-1.77) |  | 0.88(0.82-0.94) |
| Region |  |  |  |  |  |  |  |
| Asia Pacific–high income | 1.69(1.49-1.90) | 0.97(0.86-1.10) |  | 1.66(1.46-1.89) | 0.89(0.78-1.01) |  | -0.54(-0.77--0.30) |
| Central Asia | 0.88(0.78-0.97) | 1.26(1.12-1.39) |  | 2.50(2.19-2.82) | 2.75(2.41-3.11) |  | 2.89(2.69-3.08) |
| East Asia | 8.22(6.72-9.07) | 0.65(0.53-0.72) |  | 11.62(10.41-15.00) | 0.78(0.70-1.01) |  | 0.08(-0.18-0.34) |
| South Asia | 6.96(6.21-7.86) | 0.63(0.56-0.71) |  | 15.94(13.95-20.63) | 0.89(0.78-1.16) |  | 1.17(1.05-1.29) |
| Southeast Asia | 8.82(7.62-9.88) | 1.89(1.63-2.12) |  | 17.45(15.68-19.40) | 2.64(2.37-2.94) |  | 1.18(1.15-1.21) |
| Australasia | 0.14(0.13-0.16) | 0.70(0.62-0.78) |  | 0.29(0.25-0.34) | 1.03(0.89-1.18) |  | 1.64(1.37-1.91) |
| Caribbean | 0.86(0.75-0.97) | 2.44(2.13-2.76) |  | 1.39(1.20-1.67) | 3.01(2.60-3.61) |  | 0.49(0.29-0.70) |
| Central Europe | 1.79(1.62-1.99) | 1.44(1.30-1.60) |  | 2.24(2.01-2.48) | 1.95(1.75-2.16) |  | 1.01(0.79-1.23) |
| Eastern Europe | 2.96(2.73-3.18) | 1.30(1.20-1.40) |  | 8.65(8.08-9.24) | 4.11(3.84-4.40) |  | 4.62(4.03-5.22) |
| Western Europe | 6.02(4.44-7.86) | 1.56(1.15-2.04) |  | 7.03(5.26-9.16) | 1.62(1.22-2.12) |  | -0.03(-0.14-0.07) |
| Andean Latin America | 0.95(0.84-1.10) | 2.47(2.18-2.86) |  | 2.49(2.17-2.81) | 4.05(3.53-4.58) |  | 1.91(1.78-2.03) |
| Central Latin America | 4.00(3.76-4.24) | 2.44(2.29-2.58) |  | 9.52(8.87-10.14) | 3.72(3.47-3.97) |  | 1.44(1.37-1.50) |
| Southern Latin America | 0.71(0.60-0.82) | 1.43(1.20-1.66) |  | 1.20(1.00-1.41) | 1.82(1.52-2.14) |  | 1.01(0.88-1.15) |
| Tropical Latin America | 3.70(3.49-3.91) | 2.41(2.28-2.55) |  | 8.35(7.87-8.78) | 3.82(3.60-4.02) |  | 1.54(1.43-1.65) |
| North Africa and Middle East | 4.44(3.00-5.42) | 1.30(0.88-1.59) |  | 9.98(7.57-12.36) | 1.66(1.26-2.06) |  | 0.96(0.81-1.11) |
| North America–high income | 3.43(3.22-3.62) | 1.22(1.15-1.29) |  | 7.23(6.74-7.66) | 2.00(1.87-2.12) |  | 2.36(2.10-2.61) |
| Oceania | 0.06(0.05-0.07) | 0.92(0.77-1.08) |  | 0.13(0.11-0.16) | 1.03(0.86-1.24) |  | 0.48(0.39-0.57) |
| Central Sub-Saharan Africa | 0.52(0.38-0.73) | 0.95(0.69-1.32) |  | 1.15(0.88-1.47) | 0.95(0.72-1.21) |  | -0.17(-0.36-0.02) |
| Eastern Sub-Saharan Africa | 2.43(2.04-2.91) | 1.27(1.06-1.52) |  | 4.05(2.97-5.14) | 1.03(0.76-1.31) |  | -1.08(-1.18--0.97) |
| Southern Sub-Saharan Africa | 0.43(0.34-0.50) | 0.82(0.64-0.95) |  | 0.64(0.55-0.73) | 0.83(0.72-0.95) |  | -0.49(-1.06-0.08) |
| Western Sub-Saharan Africa | 2.87(1.75-4.19) | 1.49(0.91-2.18) |  | 4.51(2.97-6.22) | 1.04(0.68-1.43) |  | -1.56(-1.75--1.38) |

**Supplemental Table 5. The mortality cases, age-standardized mortality, and temporal trend of liver cirrhosis caused by other causes**

| Characteristics | 1990 | |  | 2017 | |  | 1990–2017 |
| --- | --- | --- | --- | --- | --- | --- | --- |
|  | Mortality cases No. ×10^3^ (95% UI) | ASR per 100,000 No. (95% UI) |  | Mortality cases No. ×10^3^ (95% UI) | ASR per 100,000 No. (95% UI) |  | EAPC No. (95% CI) |
| Overall | 109.64(96.65-126.68) | 2.03(1.79-2.35) |  | 146.36(130.86-164.57) | 1.92(1.71-2.15) |  | -0.33(-0.37--0.28) |
| Sex |  |  |  |  |  |  |  |
| Male | 51.60(44.84-59.68) | 1.90(1.65-2.20) |  | 70.35(62.09-80.45) | 1.83(1.62-2.10) |  | -0.21(-0.24--0.18) |
| Female | 58.04(50.47-69.82) | 2.17(1.89-2.61) |  | 76.00(66.99-89.60) | 2.00(1.76-2.35) |  | -0.43(-0.50--0.37) |
| Socio-demographic index |  |  |  |  |  |  |  |
| Low | 21.04(15.88-29.55) | 3.02(2.28-4.24) |  | 24.52(20.36-31.25) | 1.90(1.58-2.42) |  | -1.87(-1.93--1.80) |
| Low-middle | 29.29(25.82-34.07) | 2.81(2.47-3.26) |  | 41.95(35.57-48.97) | 2.46(2.09-2.87) |  | -0.62(-0.67--0.58) |
| Middle | 21.41(19.14-23.83) | 1.38(1.23-1.54) |  | 29.35(26.12-33.35) | 1.40(1.25-1.60) |  | -0.13(-0.21--0.05) |
| Middle-high | 11.78(10.43-13.17) | 1.06(0.94-1.18) |  | 17.45(15.44-19.68) | 1.26(1.11-1.42) |  | 0.65(0.53-0.77) |
| High | 25.95(22.30-30.23) | 2.69(2.31-3.13) |  | 32.91(28.82-37.48) | 2.89(2.53-3.29) |  | 0.25(0.09-0.40) |
| Region |  |  |  |  |  |  |  |
| Asia Pacific–high income | 3.58(3.10-4.45) | 2.06(1.79-2.56) |  | 3.72(3.26-4.21) | 1.99(1.74-2.25) |  | -0.24(-0.66-0.18) |
| Central Asia | 1.59(1.42-1.78) | 2.27(2.03-2.55) |  | 2.90(2.48-3.35) | 3.19(2.73-3.69) |  | 1.09(0.71-1.46) |
| East Asia | 6.75(5.29-7.72) | 0.54(0.42-0.61) |  | 6.41(5.43-8.85) | 0.43(0.37-0.60) |  | -1.40(-1.72--1.07) |
| South Asia | 27.12(22.20-36.35) | 2.45(2.00-3.28) |  | 34.92(29.91-46.16) | 1.96(1.68-2.59) |  | -1.02(-1.11--0.94) |
| Southeast Asia | 10.29(8.36-12.93) | 2.21(1.79-2.77) |  | 12.92(11.32-14.71) | 1.96(1.71-2.23) |  | -0.51(-0.55--0.47) |
| Australasia | 0.10(0.08-0.11) | 0.48(0.41-0.56) |  | 0.18(0.15-0.21) | 0.62(0.51-0.74) |  | 1.00(0.72-1.28) |
| Caribbean | 0.86(0.72-1.03) | 2.42(2.03-2.91) |  | 0.90(0.74-1.16) | 1.95(1.59-2.51) |  | -1.03(-1.29--0.77) |
| Central Europe | 1.80(1.50-2.13) | 1.45(1.21-1.71) |  | 1.87(1.53-2.27) | 1.63(1.34-1.98) |  | 0.30(0.13-0.48) |
| Eastern Europe | 3.14(2.79-3.50) | 1.39(1.23-1.54) |  | 7.08(6.36-7.81) | 3.37(3.03-3.72) |  | 3.61(3.07-4.14) |
| Western Europe | 13.76(10.76-17.46) | 3.57(2.79-4.53) |  | 14.34(11.25-17.93) | 3.31(2.60-4.14) |  | -0.47(-0.55--0.40) |
| Andean Latin America | 1.18(1.01-1.40) | 3.09(2.64-3.66) |  | 1.61(1.34-1.90) | 2.62(2.19-3.10) |  | -0.55(-0.75--0.34) |
| Central Latin America | 3.57(3.32-3.83) | 2.17(2.02-2.33) |  | 5.83(5.22-6.45) | 2.28(2.04-2.53) |  | 0.10(-0.09-0.29) |
| Southern Latin America | 1.19(1.00-1.41) | 2.41(2.01-2.84) |  | 1.78(1.45-2.12) | 2.71(2.21-3.23) |  | 0.56(0.46-0.67) |
| Tropical Latin America | 3.31(3.07-3.55) | 2.16(2.00-2.31) |  | 4.87(4.44-5.28) | 2.23(2.03-2.41) |  | -0.06(-0.23-0.10) |
| North Africa and Middle East | 5.12(3.92-7.34) | 1.50(1.15-2.15) |  | 6.34(5.38-7.48) | 1.06(0.90-1.25) |  | -1.23(-1.31--1.15) |
| North America–high income | 8.16(7.49-8.86) | 2.91(2.67-3.16) |  | 13.91(12.61-15.16) | 3.86(3.49-4.20) |  | 1.34(1.02-1.65) |
| Oceania | 0.08(0.07-0.10) | 1.26(1.06-1.55) |  | 0.15(0.12-0.19) | 1.18(0.97-1.47) |  | -0.11(-0.17--0.05) |
| Central Sub-Saharan Africa | 1.53(1.08-2.14) | 2.78(1.96-3.89) |  | 2.86(2.22-3.79) | 2.35(1.83-3.11) |  | -0.73(-0.82--0.64) |
| Eastern Sub-Saharan Africa | 6.92(5.55-8.71) | 3.61(2.90-4.55) |  | 10.34(7.55-12.92) | 2.63(1.92-3.29) |  | -1.45(-1.56--1.34) |
| Southern Sub-Saharan Africa | 0.90(0.74-1.02) | 1.71(1.40-1.95) |  | 1.02(0.84-1.20) | 1.32(1.08-1.56) |  | -1.24(-1.85--0.62) |
| Western Sub-Saharan Africa | 8.68(6.31-11.87) | 4.51(3.28-6.17) |  | 12.41(8.55-16.70) | 2.86(1.97-3.85) |  | -1.83(-1.93--1.74) |

**Supplemental Table 6. The mortality cases and temporal trend of liver cirrhosis caused by each etiology in 195 countries and territories**

| Region | Cause | Case in 1990 No.×10^3^ | Case in 2017 No.×10^3^ | | Change in absolute number (%) | EAPC (%) |
| --- | --- | --- | --- | --- | --- | --- |
| Afghanistan | Cirrhosis | 1453.04 | | 2242.99 | 54.36 | -2.80 |
| Afghanistan | Cirrhosis due to hepatitis B | 524.32 | | 729.35 | 39.10 | -3.18 |
| Afghanistan | Cirrhosis due to hepatitis C | 527.89 | | 790.56 | 49.76 | -2.91 |
| Afghanistan | Cirrhosis due to alcohol use | 83.63 | | 119.89 | 43.35 | -3.07 |
| Afghanistan | Cirrhosis due to other causes | 186.79 | | 402.96 | 115.72 | -1.56 |
| Afghanistan | Cirrhosis due to NASH | 130.40 | | 200.24 | 53.56 | -2.81 |
| Albania | Cirrhosis | 243.44 | | 308.80 | 26.85 | 1.54 |
| Albania | Cirrhosis due to hepatitis B | 71.82 | | 77.81 | 8.34 | 0.96 |
| Albania | Cirrhosis due to hepatitis C | 52.74 | | 69.14 | 31.10 | 1.66 |
| Albania | Cirrhosis due to alcohol use | 79.49 | | 112.95 | 42.11 | 1.96 |
| Albania | Cirrhosis due to other causes | 25.18 | | 26.65 | 5.84 | 0.87 |
| Albania | Cirrhosis due to NASH | 14.22 | | 22.25 | 56.45 | 2.32 |
| Algeria | Cirrhosis | 1614.37 | | 2935.12 | 81.81 | 0.50 |
| Algeria | Cirrhosis due to hepatitis B | 566.46 | | 933.26 | 64.75 | 0.13 |
| Algeria | Cirrhosis due to hepatitis C | 591.27 | | 1153.86 | 95.15 | 0.76 |
| Algeria | Cirrhosis due to alcohol use | 91.32 | | 182.85 | 100.23 | 0.86 |
| Algeria | Cirrhosis due to other causes | 224.11 | | 289.76 | 29.30 | -0.76 |
| Algeria | Cirrhosis due to NASH | 141.22 | | 375.39 | 165.81 | 1.91 |
| American Samoa | Cirrhosis | 4.26 | | 5.89 | 38.10 | 0.70 |
| American Samoa | Cirrhosis due to hepatitis B | 1.87 | | 2.41 | 28.43 | 0.43 |
| American Samoa | Cirrhosis due to hepatitis C | 0.90 | | 1.27 | 40.27 | 0.75 |
| American Samoa | Cirrhosis due to alcohol use | 0.62 | | 0.86 | 37.91 | 0.69 |
| American Samoa | Cirrhosis due to other causes | 0.35 | | 0.39 | 12.22 | -0.07 |
| American Samoa | Cirrhosis due to NASH | 0.52 | | 0.97 | 86.96 | 1.82 |
| Andorra | Cirrhosis | 6.32 | | 10.08 | 59.41 | 0.30 |
| Andorra | Cirrhosis due to hepatitis B | 0.57 | | 0.80 | 40.62 | -0.17 |
| Andorra | Cirrhosis due to hepatitis C | 1.14 | | 1.89 | 66.06 | 0.45 |
| Andorra | Cirrhosis due to alcohol use | 3.04 | | 4.60 | 51.31 | 0.11 |
| Andorra | Cirrhosis due to other causes | 1.10 | | 1.86 | 69.66 | 0.53 |
| Andorra | Cirrhosis due to NASH | 0.48 | | 0.93 | 93.99 | 1.03 |
| Angola | Cirrhosis | 2102.18 | | 5294.23 | 151.84 | -0.33 |
| Angola | Cirrhosis due to hepatitis B | 722.37 | | 1585.18 | 119.44 | -0.84 |
| Angola | Cirrhosis due to hepatitis C | 642.21 | | 1697.61 | 164.34 | -0.15 |
| Angola | Cirrhosis due to alcohol use | 318.96 | | 913.40 | 186.37 | 0.15 |
| Angola | Cirrhosis due to other causes | 313.70 | | 745.87 | 137.77 | -0.54 |
| Angola | Cirrhosis due to NASH | 104.93 | | 352.17 | 235.61 | 0.73 |
| Antigua and Barbuda | Cirrhosis | 8.46 | | 11.17 | 32.02 | -0.43 |
| Antigua and Barbuda | Cirrhosis due to hepatitis B | 1.07 | | 1.12 | 4.90 | -1.28 |
| Antigua and Barbuda | Cirrhosis due to hepatitis C | 1.93 | | 2.47 | 28.01 | -0.54 |
| Antigua and Barbuda | Cirrhosis due to alcohol use | 2.80 | | 3.71 | 32.45 | -0.42 |
| Antigua and Barbuda | Cirrhosis due to other causes | 1.11 | | 1.38 | 24.12 | -0.66 |
| Antigua and Barbuda | Cirrhosis due to NASH | 1.55 | | 2.49 | 60.61 | 0.30 |
| Argentina | Cirrhosis | 5360.49 | | 7346.32 | 37.05 | 0.09 |
| Argentina | Cirrhosis due to hepatitis B | 749.84 | | 939.11 | 25.24 | -0.24 |
| Argentina | Cirrhosis due to hepatitis C | 1487.67 | | 2125.87 | 42.90 | 0.25 |
| Argentina | Cirrhosis due to alcohol use | 2066.94 | | 2612.19 | 26.38 | -0.21 |
| Argentina | Cirrhosis due to other causes | 684.53 | | 1025.56 | 49.82 | 0.42 |
| Argentina | Cirrhosis due to NASH | 371.50 | | 643.59 | 73.24 | 0.96 |
| Armenia | Cirrhosis | 412.78 | | 1107.32 | 168.26 | 4.11 |
| Armenia | Cirrhosis due to hepatitis B | 102.61 | | 235.83 | 129.84 | 3.53 |
| Armenia | Cirrhosis due to hepatitis C | 98.91 | | 277.02 | 180.07 | 4.27 |
| Armenia | Cirrhosis due to alcohol use | 144.72 | | 386.41 | 167.01 | 4.09 |
| Armenia | Cirrhosis due to other causes | 37.85 | | 116.56 | 207.91 | 4.62 |
| Armenia | Cirrhosis due to NASH | 28.69 | | 91.49 | 218.90 | 4.75 |
| Australia | Cirrhosis | 1338.77 | | 2186.75 | 63.34 | 0.52 |
| Australia | Cirrhosis due to hepatitis B | 259.49 | | 363.31 | 40.01 | -0.05 |
| Australia | Cirrhosis due to hepatitis C | 475.87 | | 793.14 | 66.67 | 0.59 |
| Australia | Cirrhosis due to alcohol use | 396.39 | | 620.56 | 56.55 | 0.36 |
| Australia | Cirrhosis due to other causes | 84.11 | | 154.49 | 83.67 | 0.95 |
| Australia | Cirrhosis due to NASH | 122.91 | | 255.25 | 107.68 | 1.41 |
| Austria | Cirrhosis | 2287.24 | | 1860.13 | -18.67 | -1.23 |
| Austria | Cirrhosis due to hepatitis B | 185.20 | | 132.63 | -28.39 | -1.70 |
| Austria | Cirrhosis due to hepatitis C | 410.19 | | 331.00 | -19.31 | -1.25 |
| Austria | Cirrhosis due to alcohol use | 1125.67 | | 877.59 | -22.04 | -1.38 |
| Austria | Cirrhosis due to other causes | 391.62 | | 348.34 | -11.05 | -0.89 |
| Austria | Cirrhosis due to NASH | 174.55 | | 170.57 | -2.28 | -0.55 |
| Azerbaijan | Cirrhosis | 1405.77 | | 3140.76 | 123.42 | 1.74 |
| Azerbaijan | Cirrhosis due to hepatitis B | 343.13 | | 701.21 | 104.36 | 1.41 |
| Azerbaijan | Cirrhosis due to hepatitis C | 331.07 | | 758.87 | 129.22 | 1.84 |
| Azerbaijan | Cirrhosis due to alcohol use | 477.29 | | 1116.17 | 133.85 | 1.91 |
| Azerbaijan | Cirrhosis due to other causes | 156.47 | | 288.99 | 84.69 | 1.04 |
| Azerbaijan | Cirrhosis due to NASH | 97.81 | | 275.52 | 181.70 | 2.60 |
| Bahrain | Cirrhosis | 38.21 | | 84.43 | 120.99 | -1.00 |
| Bahrain | Cirrhosis due to hepatitis B | 13.71 | | 26.18 | 90.93 | -1.54 |
| Bahrain | Cirrhosis due to hepatitis C | 14.58 | | 34.09 | 133.90 | -0.79 |
| Bahrain | Cirrhosis due to alcohol use | 2.66 | | 6.16 | 131.45 | -0.83 |
| Bahrain | Cirrhosis due to other causes | 3.12 | | 5.11 | 63.90 | -2.11 |
| Bahrain | Cirrhosis due to NASH | 4.14 | | 12.89 | 211.36 | 0.27 |
| Bangladesh | Cirrhosis | 22244.55 | | 26389.93 | 18.64 | -0.72 |
| Bangladesh | Cirrhosis due to hepatitis B | 6642.49 | | 7439.74 | 12.00 | -0.93 |
| Bangladesh | Cirrhosis due to hepatitis C | 4548.30 | | 6464.79 | 42.14 | -0.05 |
| Bangladesh | Cirrhosis due to alcohol use | 4801.72 | | 7302.44 | 52.08 | 0.20 |
| Bangladesh | Cirrhosis due to other causes | 5420.47 | | 3705.47 | -31.64 | -2.76 |
| Bangladesh | Cirrhosis due to NASH | 831.58 | | 1477.50 | 77.67 | 0.77 |
| Barbados | Cirrhosis | 35.40 | | 38.69 | 9.32 | -0.24 |
| Barbados | Cirrhosis due to hepatitis B | 4.39 | | 4.03 | -8.28 | -0.89 |
| Barbados | Cirrhosis due to hepatitis C | 7.76 | | 8.54 | 10.14 | -0.21 |
| Barbados | Cirrhosis due to alcohol use | 12.05 | | 13.26 | 10.06 | -0.21 |
| Barbados | Cirrhosis due to other causes | 4.58 | | 4.43 | -3.18 | -0.69 |
| Barbados | Cirrhosis due to NASH | 6.62 | | 8.43 | 27.31 | 0.33 |
| Belarus | Cirrhosis | 865.48 | | 2533.23 | 192.70 | 4.34 |
| Belarus | Cirrhosis due to hepatitis B | 167.72 | | 423.90 | 152.75 | 3.79 |
| Belarus | Cirrhosis due to hepatitis C | 197.39 | | 588.00 | 197.89 | 4.40 |
| Belarus | Cirrhosis due to alcohol use | 333.96 | | 1027.01 | 207.52 | 4.52 |
| Belarus | Cirrhosis due to other causes | 88.79 | | 223.78 | 152.03 | 3.78 |
| Belarus | Cirrhosis due to NASH | 77.62 | | 270.55 | 248.55 | 4.98 |
| Belgium | Cirrhosis | 1701.52 | | 2004.40 | 17.80 | 0.14 |
| Belgium | Cirrhosis due to hepatitis B | 191.46 | | 191.50 | 0.02 | -0.47 |
| Belgium | Cirrhosis due to hepatitis C | 252.45 | | 293.01 | 16.07 | 0.08 |
| Belgium | Cirrhosis due to alcohol use | 901.78 | | 1072.99 | 18.99 | 0.18 |
| Belgium | Cirrhosis due to other causes | 251.84 | | 296.61 | 17.78 | 0.14 |
| Belgium | Cirrhosis due to NASH | 103.98 | | 150.28 | 44.52 | 0.90 |
| Belize | Cirrhosis | 21.51 | | 57.17 | 165.74 | 0.88 |
| Belize | Cirrhosis due to hepatitis B | 2.62 | | 6.05 | 130.76 | 0.36 |
| Belize | Cirrhosis due to hepatitis C | 4.65 | | 12.80 | 175.02 | 1.01 |
| Belize | Cirrhosis due to alcohol use | 6.93 | | 19.75 | 184.92 | 1.14 |
| Belize | Cirrhosis due to other causes | 3.80 | | 6.76 | 77.92 | -0.61 |
| Belize | Cirrhosis due to NASH | 3.51 | | 11.81 | 236.72 | 1.76 |
| Benin | Cirrhosis | 898.65 | | 1269.50 | 41.27 | -1.95 |
| Benin | Cirrhosis due to hepatitis B | 474.42 | | 633.97 | 33.63 | -2.16 |
| Benin | Cirrhosis due to hepatitis C | 66.79 | | 98.86 | 48.02 | -1.78 |
| Benin | Cirrhosis due to alcohol use | 138.52 | | 214.06 | 54.53 | -1.62 |
| Benin | Cirrhosis due to other causes | 173.12 | | 233.30 | 34.76 | -2.13 |
| Benin | Cirrhosis due to NASH | 45.79 | | 89.31 | 95.02 | -0.76 |
| Bermuda | Cirrhosis | 8.73 | | 6.99 | -20.01 | -1.22 |
| Bermuda | Cirrhosis due to hepatitis B | 0.95 | | 0.77 | -19.51 | -1.19 |
| Bermuda | Cirrhosis due to hepatitis C | 1.74 | | 1.40 | -19.60 | -1.20 |
| Bermuda | Cirrhosis due to alcohol use | 3.48 | | 2.52 | -27.63 | -1.59 |
| Bermuda | Cirrhosis due to other causes | 0.96 | | 0.76 | -20.35 | -1.23 |
| Bermuda | Cirrhosis due to NASH | 1.60 | | 1.53 | -3.95 | -0.54 |
| Bhutan | Cirrhosis | 107.11 | | 156.90 | 46.48 | -0.56 |
| Bhutan | Cirrhosis due to hepatitis B | 33.28 | | 45.42 | 36.48 | -0.82 |
| Bhutan | Cirrhosis due to hepatitis C | 22.56 | | 36.37 | 61.19 | -0.20 |
| Bhutan | Cirrhosis due to alcohol use | 29.19 | | 47.16 | 61.54 | -0.20 |
| Bhutan | Cirrhosis due to other causes | 17.17 | | 18.66 | 8.62 | -1.67 |
| Bhutan | Cirrhosis due to NASH | 4.90 | | 9.29 | 89.74 | 0.40 |
| Bolivia | Cirrhosis | 1101.54 | | 2215.41 | 101.12 | 0.44 |
| Bolivia | Cirrhosis due to hepatitis B | 151.89 | | 256.99 | 69.20 | -0.20 |
| Bolivia | Cirrhosis due to hepatitis C | 148.30 | | 312.39 | 110.65 | 0.61 |
| Bolivia | Cirrhosis due to alcohol use | 391.72 | | 819.02 | 109.08 | 0.58 |
| Bolivia | Cirrhosis due to other causes | 229.26 | | 330.42 | 44.13 | -0.80 |
| Bolivia | Cirrhosis due to NASH | 180.38 | | 496.59 | 175.31 | 1.60 |
| Bosnia and Herzegovina | Cirrhosis | 743.58 | | 577.39 | -22.35 | 0.11 |
| Bosnia and Herzegovina | Cirrhosis due to hepatitis B | 217.39 | | 147.59 | -32.11 | -0.39 |
| Bosnia and Herzegovina | Cirrhosis due to hepatitis C | 167.19 | | 132.90 | -20.51 | 0.20 |
| Bosnia and Herzegovina | Cirrhosis due to alcohol use | 263.54 | | 209.17 | -20.63 | 0.19 |
| Bosnia and Herzegovina | Cirrhosis due to other causes | 49.18 | | 41.22 | -16.18 | 0.39 |
| Bosnia and Herzegovina | Cirrhosis due to NASH | 46.28 | | 46.51 | 0.50 | 1.07 |
| Botswana | Cirrhosis | 118.06 | | 187.43 | 58.76 | -0.34 |
| Botswana | Cirrhosis due to hepatitis B | 30.78 | | 40.81 | 32.58 | -1.01 |
| Botswana | Cirrhosis due to hepatitis C | 41.19 | | 66.81 | 62.21 | -0.26 |
| Botswana | Cirrhosis due to alcohol use | 21.90 | | 36.23 | 65.42 | -0.19 |
| Botswana | Cirrhosis due to other causes | 17.04 | | 27.69 | 62.45 | -0.26 |
| Botswana | Cirrhosis due to NASH | 7.14 | | 15.89 | 122.48 | 0.91 |
| Brazil | Cirrhosis | 22134.43 | | 36269.34 | 63.86 | 0.54 |
| Brazil | Cirrhosis due to hepatitis B | 4309.34 | | 5774.20 | 33.99 | -0.21 |
| Brazil | Cirrhosis due to hepatitis C | 5971.31 | | 9519.90 | 59.43 | 0.44 |
| Brazil | Cirrhosis due to alcohol use | 4946.07 | | 7984.20 | 61.43 | 0.48 |
| Brazil | Cirrhosis due to other causes | 3257.97 | | 4778.28 | 46.66 | 0.13 |
| Brazil | Cirrhosis due to NASH | 3649.72 | | 8212.75 | 125.02 | 1.71 |
| Brunei | Cirrhosis | 14.25 | | 24.52 | 72.07 | 0.11 |
| Brunei | Cirrhosis due to hepatitis B | 5.42 | | 8.30 | 53.17 | -0.33 |
| Brunei | Cirrhosis due to hepatitis C | 3.13 | | 5.82 | 85.68 | 0.39 |
| Brunei | Cirrhosis due to alcohol use | 3.43 | | 6.28 | 83.10 | 0.34 |
| Brunei | Cirrhosis due to other causes | 1.70 | | 2.86 | 68.48 | 0.03 |
| Brunei | Cirrhosis due to NASH | 0.57 | | 1.27 | 121.00 | 1.03 |
| Bulgaria | Cirrhosis | 1867.38 | | 2043.05 | 9.41 | 1.20 |
| Bulgaria | Cirrhosis due to hepatitis B | 483.19 | | 468.78 | -2.98 | 0.76 |
| Bulgaria | Cirrhosis due to hepatitis C | 358.69 | | 421.15 | 17.41 | 1.46 |
| Bulgaria | Cirrhosis due to alcohol use | 802.37 | | 893.92 | 11.41 | 1.27 |
| Bulgaria | Cirrhosis due to other causes | 109.26 | | 119.08 | 8.99 | 1.19 |
| Bulgaria | Cirrhosis due to NASH | 113.85 | | 140.11 | 23.06 | 1.64 |
| Burkina Faso | Cirrhosis | 1847.20 | | 2089.52 | 13.12 | -2.48 |
| Burkina Faso | Cirrhosis due to hepatitis B | 1011.19 | | 1033.92 | 2.25 | -2.85 |
| Burkina Faso | Cirrhosis due to hepatitis C | 130.60 | | 159.67 | 22.26 | -2.19 |
| Burkina Faso | Cirrhosis due to alcohol use | 295.38 | | 365.51 | 23.74 | -2.15 |
| Burkina Faso | Cirrhosis due to other causes | 323.06 | | 405.91 | 25.65 | -2.09 |
| Burkina Faso | Cirrhosis due to NASH | 86.97 | | 124.51 | 43.16 | -1.61 |
| Burundi | Cirrhosis | 1671.05 | | 1718.91 | 2.86 | -2.43 |
| Burundi | Cirrhosis due to hepatitis B | 425.24 | | 395.23 | -7.06 | -2.81 |
| Burundi | Cirrhosis due to hepatitis C | 488.66 | | 529.64 | 8.39 | -2.24 |
| Burundi | Cirrhosis due to alcohol use | 375.56 | | 398.77 | 6.18 | -2.31 |
| Burundi | Cirrhosis due to other causes | 280.11 | | 284.44 | 1.55 | -2.48 |
| Burundi | Cirrhosis due to NASH | 101.48 | | 110.82 | 9.20 | -2.21 |
| Cambodia | Cirrhosis | 4236.79 | | 9017.62 | 112.84 | 1.18 |
| Cambodia | Cirrhosis due to hepatitis B | 1591.82 | | 3146.58 | 97.67 | 0.91 |
| Cambodia | Cirrhosis due to hepatitis C | 1157.95 | | 2670.10 | 130.59 | 1.48 |
| Cambodia | Cirrhosis due to alcohol use | 577.01 | | 1495.61 | 159.20 | 1.91 |
| Cambodia | Cirrhosis due to other causes | 530.31 | | 770.05 | 45.21 | -0.23 |
| Cambodia | Cirrhosis due to NASH | 379.70 | | 935.27 | 146.32 | 1.73 |
| Cameroon | Cirrhosis | 2450.95 | | 4080.35 | 66.48 | -1.77 |
| Cameroon | Cirrhosis due to hepatitis B | 1254.48 | | 2011.63 | 60.36 | -1.90 |
| Cameroon | Cirrhosis due to hepatitis C | 197.64 | | 333.89 | 68.94 | -1.71 |
| Cameroon | Cirrhosis due to alcohol use | 423.73 | | 759.66 | 79.28 | -1.49 |
| Cameroon | Cirrhosis due to other causes | 409.79 | | 653.36 | 59.43 | -1.93 |
| Cameroon | Cirrhosis due to NASH | 165.30 | | 321.82 | 94.69 | -1.19 |
| Canada | Cirrhosis | 2944.08 | | 4844.52 | 64.55 | 0.81 |
| Canada | Cirrhosis due to hepatitis B | 185.92 | | 288.63 | 55.25 | 0.60 |
| Canada | Cirrhosis due to hepatitis C | 937.21 | | 1501.02 | 60.16 | 0.71 |
| Canada | Cirrhosis due to alcohol use | 959.39 | | 1539.67 | 60.48 | 0.72 |
| Canada | Cirrhosis due to other causes | 615.82 | | 1061.99 | 72.45 | 0.99 |
| Canada | Cirrhosis due to NASH | 245.73 | | 453.20 | 84.43 | 1.24 |
| Cape Verde | Cirrhosis | 37.12 | | 86.06 | 131.86 | 1.49 |
| Cape Verde | Cirrhosis due to hepatitis B | 19.77 | | 42.66 | 115.75 | 1.22 |
| Cape Verde | Cirrhosis due to hepatitis C | 2.90 | | 7.53 | 159.52 | 1.91 |
| Cape Verde | Cirrhosis due to alcohol use | 6.12 | | 16.62 | 171.73 | 2.08 |
| Cape Verde | Cirrhosis due to other causes | 6.28 | | 12.67 | 101.75 | 0.97 |
| Cape Verde | Cirrhosis due to NASH | 2.05 | | 6.58 | 221.47 | 2.70 |
| Central African Republic | Cirrhosis | 537.79 | | 698.68 | 29.92 | -0.98 |
| Central African Republic | Cirrhosis due to hepatitis B | 179.14 | | 226.44 | 26.40 | -1.08 |
| Central African Republic | Cirrhosis due to hepatitis C | 169.10 | | 226.05 | 33.68 | -0.87 |
| Central African Republic | Cirrhosis due to alcohol use | 90.26 | | 115.64 | 28.12 | -1.03 |
| Central African Republic | Cirrhosis due to other causes | 72.58 | | 93.78 | 29.20 | -1.00 |
| Central African Republic | Cirrhosis due to NASH | 26.70 | | 36.77 | 37.71 | -0.76 |
| Chad | Cirrhosis | 1024.40 | | 2040.32 | 99.17 | -0.87 |
| Chad | Cirrhosis due to hepatitis B | 570.72 | | 1118.31 | 95.95 | -0.93 |
| Chad | Cirrhosis due to hepatitis C | 76.07 | | 151.33 | 98.93 | -0.88 |
| Chad | Cirrhosis due to alcohol use | 149.10 | | 325.51 | 118.32 | -0.53 |
| Chad | Cirrhosis due to other causes | 179.48 | | 343.31 | 91.28 | -1.02 |
| Chad | Cirrhosis due to NASH | 49.03 | | 101.86 | 107.74 | -0.72 |
| Chile | Cirrhosis | 3717.99 | | 4980.29 | 33.95 | -0.03 |
| Chile | Cirrhosis due to hepatitis B | 553.26 | | 642.68 | 16.16 | -0.55 |
| Chile | Cirrhosis due to hepatitis C | 1111.33 | | 1488.79 | 33.96 | -0.03 |
| Chile | Cirrhosis due to alcohol use | 1323.52 | | 1678.32 | 26.81 | -0.23 |
| Chile | Cirrhosis due to other causes | 434.79 | | 668.73 | 53.80 | 0.49 |
| Chile | Cirrhosis due to NASH | 295.09 | | 501.77 | 70.04 | 0.86 |
| China | Cirrhosis | 150718.36 | | 153769.46 | 2.02 | -0.54 |
| China | Cirrhosis due to hepatitis B | 81337.42 | | 75158.77 | -7.60 | -0.91 |
| China | Cirrhosis due to hepatitis C | 31317.18 | | 34198.30 | 9.20 | -0.29 |
| China | Cirrhosis due to alcohol use | 24055.09 | | 27845.68 | 15.76 | -0.07 |
| China | Cirrhosis due to other causes | 6340.86 | | 5841.82 | -7.87 | -0.92 |
| China | Cirrhosis due to NASH | 7667.81 | | 10724.90 | 39.87 | 0.63 |
| Colombia | Cirrhosis | 1953.39 | | 3886.21 | 98.95 | 0.92 |
| Colombia | Cirrhosis due to hepatitis B | 154.82 | | 266.24 | 71.96 | 0.38 |
| Colombia | Cirrhosis due to hepatitis C | 540.98 | | 1079.69 | 99.58 | 0.94 |
| Colombia | Cirrhosis due to alcohol use | 784.23 | | 1559.41 | 98.85 | 0.92 |
| Colombia | Cirrhosis due to other causes | 251.07 | | 411.85 | 64.04 | 0.21 |
| Colombia | Cirrhosis due to NASH | 222.29 | | 569.02 | 155.98 | 1.86 |
| Comoros | Cirrhosis | 46.50 | | 87.69 | 88.57 | 0.72 |
| Comoros | Cirrhosis due to hepatitis B | 12.41 | | 21.57 | 73.74 | 0.42 |
| Comoros | Cirrhosis due to hepatitis C | 14.04 | | 28.20 | 100.81 | 0.95 |
| Comoros | Cirrhosis due to alcohol use | 7.66 | | 15.18 | 98.27 | 0.91 |
| Comoros | Cirrhosis due to other causes | 9.28 | | 16.06 | 73.08 | 0.40 |
| Comoros | Cirrhosis due to NASH | 3.11 | | 6.69 | 114.83 | 1.20 |
| Congo | Cirrhosis | 589.08 | | 1014.05 | 72.14 | -0.60 |
| Congo | Cirrhosis due to hepatitis B | 185.74 | | 307.36 | 65.48 | -0.74 |
| Congo | Cirrhosis due to hepatitis C | 186.66 | | 334.19 | 79.04 | -0.45 |
| Congo | Cirrhosis due to alcohol use | 100.45 | | 175.23 | 74.44 | -0.55 |
| Congo | Cirrhosis due to other causes | 84.59 | | 132.73 | 56.91 | -0.94 |
| Congo | Cirrhosis due to NASH | 31.64 | | 64.54 | 104.00 | 0.03 |
| Costa Rica | Cirrhosis | 309.29 | | 840.40 | 171.71 | 2.13 |
| Costa Rica | Cirrhosis due to hepatitis B | 22.31 | | 51.92 | 132.76 | 1.55 |
| Costa Rica | Cirrhosis due to hepatitis C | 89.35 | | 237.69 | 166.01 | 2.05 |
| Costa Rica | Cirrhosis due to alcohol use | 130.02 | | 343.61 | 164.27 | 2.02 |
| Costa Rica | Cirrhosis due to other causes | 28.48 | | 70.98 | 149.28 | 1.81 |
| Costa Rica | Cirrhosis due to NASH | 39.14 | | 136.18 | 247.96 | 3.04 |
| Cote d'Ivoire | Cirrhosis | 1971.52 | | 3510.54 | 78.06 | -0.50 |
| Cote d'Ivoire | Cirrhosis due to hepatitis B | 1052.04 | | 1810.22 | 72.07 | -0.63 |
| Cote d'Ivoire | Cirrhosis due to hepatitis C | 154.90 | | 285.58 | 84.36 | -0.37 |
| Cote d'Ivoire | Cirrhosis due to alcohol use | 337.00 | | 648.76 | 92.51 | -0.21 |
| Cote d'Ivoire | Cirrhosis due to other causes | 328.84 | | 547.35 | 66.45 | -0.75 |
| Cote d'Ivoire | Cirrhosis due to NASH | 98.74 | | 218.63 | 121.41 | 0.31 |
| Croatia | Cirrhosis | 1756.05 | | 1095.27 | -37.63 | -1.24 |
| Croatia | Cirrhosis due to hepatitis B | 459.14 | | 254.26 | -44.62 | -1.68 |
| Croatia | Cirrhosis due to hepatitis C | 346.43 | | 222.17 | -35.87 | -1.14 |
| Croatia | Cirrhosis due to alcohol use | 745.56 | | 470.57 | -36.88 | -1.20 |
| Croatia | Cirrhosis due to other causes | 97.09 | | 68.87 | -29.07 | -0.77 |
| Croatia | Cirrhosis due to NASH | 107.84 | | 79.40 | -26.38 | -0.63 |
| Cuba | Cirrhosis | 1109.98 | | 1868.81 | 68.36 | 1.75 |
| Cuba | Cirrhosis due to hepatitis B | 139.45 | | 194.14 | 39.22 | 1.05 |
| Cuba | Cirrhosis due to hepatitis C | 253.72 | | 429.82 | 69.41 | 1.77 |
| Cuba | Cirrhosis due to alcohol use | 354.44 | | 649.46 | 83.23 | 2.06 |
| Cuba | Cirrhosis due to other causes | 165.63 | | 208.19 | 25.70 | 0.67 |
| Cuba | Cirrhosis due to NASH | 196.74 | | 387.20 | 96.81 | 2.33 |
| Cyprus | Cirrhosis | 89.79 | | 122.89 | 36.87 | -0.64 |
| Cyprus | Cirrhosis due to hepatitis B | 8.22 | | 10.03 | 22.00 | -1.07 |
| Cyprus | Cirrhosis due to hepatitis C | 16.06 | | 22.27 | 38.65 | -0.59 |
| Cyprus | Cirrhosis due to alcohol use | 38.25 | | 52.16 | 36.36 | -0.65 |
| Cyprus | Cirrhosis due to other causes | 19.80 | | 26.25 | 32.62 | -0.76 |
| Cyprus | Cirrhosis due to NASH | 7.46 | | 12.17 | 63.26 | 0.01 |
| Czech Republic | Cirrhosis | 2173.73 | | 2174.96 | 0.06 | -0.11 |
| Czech Republic | Cirrhosis due to hepatitis B | 549.09 | | 462.01 | -15.86 | -0.75 |
| Czech Republic | Cirrhosis due to hepatitis C | 417.95 | | 433.20 | 3.65 | 0.02 |
| Czech Republic | Cirrhosis due to alcohol use | 957.40 | | 1000.76 | 4.53 | 0.05 |
| Czech Republic | Cirrhosis due to other causes | 124.09 | | 128.19 | 3.30 | 0.01 |
| Czech Republic | Cirrhosis due to NASH | 125.20 | | 150.80 | 20.45 | 0.58 |
| Democratic Republic of the Congo | Cirrhosis | 6770.96 | | 12752.83 | 88.35 | -0.43 |
| Democratic Republic of the Congo | Cirrhosis due to hepatitis B | 2286.32 | | 4054.61 | 77.34 | -0.66 |
| Democratic Republic of the Congo | Cirrhosis due to hepatitis C | 2088.60 | | 4133.53 | 97.91 | -0.25 |
| Democratic Republic of the Congo | Cirrhosis due to alcohol use | 1052.27 | | 2100.53 | 99.62 | -0.22 |
| Democratic Republic of the Congo | Cirrhosis due to other causes | 1006.15 | | 1811.32 | 80.02 | -0.60 |
| Democratic Republic of the Congo | Cirrhosis due to NASH | 337.61 | | 652.84 | 93.37 | -0.34 |
| Denmark | Cirrhosis | 734.21 | | 947.18 | 29.01 | 0.54 |
| Denmark | Cirrhosis due to hepatitis B | 64.44 | | 79.28 | 23.03 | 0.36 |
| Denmark | Cirrhosis due to hepatitis C | 132.56 | | 162.95 | 22.92 | 0.36 |
| Denmark | Cirrhosis due to alcohol use | 364.55 | | 447.55 | 22.77 | 0.36 |
| Denmark | Cirrhosis due to other causes | 116.10 | | 169.86 | 46.30 | 1.00 |
| Denmark | Cirrhosis due to NASH | 56.55 | | 87.55 | 54.80 | 1.21 |
| Djibouti | Cirrhosis | 46.29 | | 143.13 | 209.23 | 1.20 |
| Djibouti | Cirrhosis due to hepatitis B | 12.45 | | 36.79 | 195.42 | 1.04 |
| Djibouti | Cirrhosis due to hepatitis C | 13.51 | | 43.71 | 223.61 | 1.37 |
| Djibouti | Cirrhosis due to alcohol use | 8.65 | | 28.69 | 231.46 | 1.46 |
| Djibouti | Cirrhosis due to other causes | 8.78 | | 22.13 | 151.94 | 0.45 |
| Djibouti | Cirrhosis due to NASH | 2.89 | | 11.82 | 309.04 | 2.24 |
| Dominica | Cirrhosis | 9.68 | | 9.86 | 1.87 | 0.33 |
| Dominica | Cirrhosis due to hepatitis B | 1.14 | | 1.03 | -10.00 | -0.13 |
| Dominica | Cirrhosis due to hepatitis C | 2.09 | | 2.15 | 2.89 | 0.37 |
| Dominica | Cirrhosis due to alcohol use | 3.37 | | 3.41 | 1.07 | 0.30 |
| Dominica | Cirrhosis due to other causes | 1.45 | | 1.30 | -10.36 | -0.14 |
| Dominica | Cirrhosis due to NASH | 1.62 | | 1.97 | 21.46 | 0.98 |
| Dominican Republic | Cirrhosis | 1398.06 | | 1611.90 | 15.30 | -0.85 |
| Dominican Republic | Cirrhosis due to hepatitis B | 167.13 | | 173.36 | 3.73 | -1.24 |
| Dominican Republic | Cirrhosis due to hepatitis C | 294.74 | | 356.62 | 20.99 | -0.67 |
| Dominican Republic | Cirrhosis due to alcohol use | 455.19 | | 570.76 | 25.39 | -0.54 |
| Dominican Republic | Cirrhosis due to other causes | 280.33 | | 220.70 | -21.27 | -2.27 |
| Dominican Republic | Cirrhosis due to NASH | 200.67 | | 290.46 | 44.75 | -0.01 |
| Ecuador | Cirrhosis | 1420.85 | | 3456.61 | 143.28 | 1.40 |
| Ecuador | Cirrhosis due to hepatitis B | 217.98 | | 412.36 | 89.17 | 0.47 |
| Ecuador | Cirrhosis due to hepatitis C | 209.46 | | 499.40 | 138.42 | 1.33 |
| Ecuador | Cirrhosis due to alcohol use | 467.66 | | 1139.62 | 143.68 | 1.41 |
| Ecuador | Cirrhosis due to other causes | 251.87 | | 534.18 | 112.09 | 0.90 |
| Ecuador | Cirrhosis due to NASH | 273.88 | | 871.05 | 218.04 | 2.40 |
| Egypt | Cirrhosis | 29306.55 | | 44692.44 | 52.50 | -0.52 |
| Egypt | Cirrhosis due to hepatitis B | 13378.13 | | 18552.80 | 38.68 | -0.87 |
| Egypt | Cirrhosis due to hepatitis C | 9766.11 | | 15377.45 | 57.46 | -0.40 |
| Egypt | Cirrhosis due to alcohol use | 1302.41 | | 2131.87 | 63.69 | -0.26 |
| Egypt | Cirrhosis due to other causes | 2261.38 | | 2915.13 | 28.91 | -1.14 |
| Egypt | Cirrhosis due to NASH | 2598.51 | | 5715.19 | 119.94 | 0.84 |
| El Salvador | Cirrhosis | 913.65 | | 1479.66 | 61.95 | 1.23 |
| El Salvador | Cirrhosis due to hepatitis B | 74.25 | | 98.60 | 32.80 | 0.50 |
| El Salvador | Cirrhosis due to hepatitis C | 279.59 | | 447.21 | 59.95 | 1.19 |
| El Salvador | Cirrhosis due to alcohol use | 324.20 | | 544.12 | 67.83 | 1.37 |
| El Salvador | Cirrhosis due to other causes | 121.95 | | 140.41 | 15.14 | -0.03 |
| El Salvador | Cirrhosis due to NASH | 113.66 | | 249.32 | 119.36 | 2.36 |
| Equatorial Guinea | Cirrhosis | 78.09 | | 148.98 | 90.77 | -1.89 |
| Equatorial Guinea | Cirrhosis due to hepatitis B | 25.29 | | 43.51 | 72.04 | -2.27 |
| Equatorial Guinea | Cirrhosis due to hepatitis C | 24.42 | | 45.82 | 87.65 | -1.95 |
| Equatorial Guinea | Cirrhosis due to alcohol use | 12.78 | | 25.99 | 103.37 | -1.65 |
| Equatorial Guinea | Cirrhosis due to other causes | 11.22 | | 20.42 | 82.04 | -2.06 |
| Equatorial Guinea | Cirrhosis due to NASH | 4.39 | | 13.25 | 201.54 | -0.19 |
| Eritrea | Cirrhosis | 519.34 | | 1112.14 | 114.15 | 0.21 |
| Eritrea | Cirrhosis due to hepatitis B | 144.66 | | 277.00 | 91.49 | -0.21 |
| Eritrea | Cirrhosis due to hepatitis C | 157.74 | | 352.13 | 123.24 | 0.36 |
| Eritrea | Cirrhosis due to alcohol use | 98.33 | | 218.85 | 122.56 | 0.35 |
| Eritrea | Cirrhosis due to other causes | 89.11 | | 194.18 | 117.91 | 0.27 |
| Eritrea | Cirrhosis due to NASH | 29.50 | | 69.97 | 137.20 | 0.59 |
| Estonia | Cirrhosis | 139.88 | | 292.22 | 108.91 | 3.38 |
| Estonia | Cirrhosis due to hepatitis B | 28.13 | | 47.24 | 67.96 | 2.57 |
| Estonia | Cirrhosis due to hepatitis C | 32.60 | | 65.24 | 100.12 | 3.22 |
| Estonia | Cirrhosis due to alcohol use | 53.09 | | 126.36 | 138.02 | 3.87 |
| Estonia | Cirrhosis due to other causes | 13.06 | | 23.26 | 78.14 | 2.79 |
| Estonia | Cirrhosis due to NASH | 13.01 | | 30.12 | 131.55 | 3.76 |
| Ethiopia | Cirrhosis | 10886.15 | | 16068.94 | 47.61 | -1.13 |
| Ethiopia | Cirrhosis due to hepatitis B | 3110.38 | | 4707.65 | 51.35 | -1.03 |
| Ethiopia | Cirrhosis due to hepatitis C | 3748.78 | | 5444.40 | 45.23 | -1.19 |
| Ethiopia | Cirrhosis due to alcohol use | 1743.19 | | 2610.25 | 49.74 | -1.07 |
| Ethiopia | Cirrhosis due to other causes | 1689.89 | | 2412.78 | 42.78 | -1.25 |
| Ethiopia | Cirrhosis due to NASH | 593.91 | | 893.86 | 50.50 | -1.06 |
| Federated States of Micronesia | Cirrhosis | 16.21 | | 14.05 | -13.30 | -0.53 |
| Federated States of Micronesia | Cirrhosis due to hepatitis B | 7.66 | | 5.81 | -24.15 | -1.03 |
| Federated States of Micronesia | Cirrhosis due to hepatitis C | 3.48 | | 3.35 | -3.56 | -0.14 |
| Federated States of Micronesia | Cirrhosis due to alcohol use | 2.26 | | 2.16 | -4.50 | -0.18 |
| Federated States of Micronesia | Cirrhosis due to other causes | 1.56 | | 1.14 | -26.82 | -1.16 |
| Federated States of Micronesia | Cirrhosis due to NASH | 1.26 | | 1.59 | 26.81 | 0.87 |
| Fiji | Cirrhosis | 37.84 | | 65.67 | 73.54 | 1.40 |
| Fiji | Cirrhosis due to hepatitis B | 17.18 | | 26.47 | 54.12 | 0.96 |
| Fiji | Cirrhosis due to hepatitis C | 8.65 | | 16.13 | 86.58 | 1.67 |
| Fiji | Cirrhosis due to alcohol use | 5.39 | | 10.37 | 92.37 | 1.78 |
| Fiji | Cirrhosis due to other causes | 3.63 | | 5.55 | 52.91 | 0.93 |
| Fiji | Cirrhosis due to NASH | 2.99 | | 7.14 | 138.50 | 2.58 |
| Finland | Cirrhosis | 572.09 | | 1177.54 | 105.83 | 2.31 |
| Finland | Cirrhosis due to hepatitis B | 54.20 | | 105.22 | 94.15 | 2.09 |
| Finland | Cirrhosis due to hepatitis C | 109.48 | | 216.53 | 97.78 | 2.16 |
| Finland | Cirrhosis due to alcohol use | 259.19 | | 531.66 | 105.12 | 2.30 |
| Finland | Cirrhosis due to other causes | 99.72 | | 196.10 | 96.65 | 2.14 |
| Finland | Cirrhosis due to NASH | 49.51 | | 128.04 | 158.62 | 3.16 |
| France | Cirrhosis | 13236.07 | | 10621.09 | -19.76 | -1.30 |
| France | Cirrhosis due to hepatitis B | 1086.73 | | 839.50 | -22.75 | -1.44 |
| France | Cirrhosis due to hepatitis C | 2245.39 | | 1860.89 | -17.12 | -1.18 |
| France | Cirrhosis due to alcohol use | 6596.36 | | 4875.73 | -26.08 | -1.60 |
| France | Cirrhosis due to other causes | 2372.24 | | 2096.31 | -11.63 | -0.94 |
| France | Cirrhosis due to NASH | 935.35 | | 948.65 | 1.42 | -0.43 |
| Gabon | Cirrhosis | 341.01 | | 421.44 | 23.59 | -1.26 |
| Gabon | Cirrhosis due to hepatitis B | 103.77 | | 118.35 | 14.05 | -1.56 |
| Gabon | Cirrhosis due to hepatitis C | 109.66 | | 142.08 | 29.57 | -1.09 |
| Gabon | Cirrhosis due to alcohol use | 64.19 | | 77.68 | 21.02 | -1.34 |
| Gabon | Cirrhosis due to other causes | 43.83 | | 52.22 | 19.14 | -1.40 |
| Gabon | Cirrhosis due to NASH | 19.57 | | 31.11 | 58.96 | -0.33 |
| Georgia | Cirrhosis | 1475.15 | | 1381.13 | -6.37 | 1.24 |
| Georgia | Cirrhosis due to hepatitis B | 353.64 | | 302.00 | -14.60 | 0.90 |
| Georgia | Cirrhosis due to hepatitis C | 347.32 | | 334.01 | -3.83 | 1.34 |
| Georgia | Cirrhosis due to alcohol use | 552.17 | | 531.67 | -3.71 | 1.34 |
| Georgia | Cirrhosis due to other causes | 117.47 | | 100.82 | -14.17 | 0.92 |
| Georgia | Cirrhosis due to NASH | 104.56 | | 112.64 | 7.73 | 1.76 |
| Germany | Cirrhosis | 20334.44 | | 19557.64 | -3.82 | -0.29 |
| Germany | Cirrhosis due to hepatitis B | 1703.62 | | 1440.09 | -15.47 | -0.77 |
| Germany | Cirrhosis due to hepatitis C | 3468.84 | | 3357.51 | -3.21 | -0.27 |
| Germany | Cirrhosis due to alcohol use | 9796.72 | | 8892.68 | -9.23 | -0.51 |
| Germany | Cirrhosis due to other causes | 3724.69 | | 3891.83 | 4.49 | 0.02 |
| Germany | Cirrhosis due to NASH | 1640.57 | | 1975.52 | 20.42 | 0.54 |
| Ghana | Cirrhosis | 2749.11 | | 4004.00 | 45.65 | -1.22 |
| Ghana | Cirrhosis due to hepatitis B | 1406.97 | | 1939.31 | 37.84 | -1.42 |
| Ghana | Cirrhosis due to hepatitis C | 231.30 | | 347.03 | 50.03 | -1.11 |
| Ghana | Cirrhosis due to alcohol use | 472.45 | | 732.60 | 55.06 | -0.98 |
| Ghana | Cirrhosis due to other causes | 484.47 | | 682.85 | 40.95 | -1.34 |
| Ghana | Cirrhosis due to NASH | 153.92 | | 302.22 | 96.35 | -0.11 |
| Greece | Cirrhosis | 1598.32 | | 1307.96 | -18.17 | -0.74 |
| Greece | Cirrhosis due to hepatitis B | 183.71 | | 131.68 | -28.32 | -1.23 |
| Greece | Cirrhosis due to hepatitis C | 258.56 | | 217.83 | -15.75 | -0.63 |
| Greece | Cirrhosis due to alcohol use | 689.54 | | 542.86 | -21.27 | -0.88 |
| Greece | Cirrhosis due to other causes | 338.23 | | 283.50 | -16.18 | -0.65 |
| Greece | Cirrhosis due to NASH | 128.28 | | 132.09 | 2.97 | 0.11 |
| Greenland | Cirrhosis | 4.36 | | 6.90 | 58.47 | 1.67 |
| Greenland | Cirrhosis due to hepatitis B | 0.27 | | 0.42 | 55.71 | 1.60 |
| Greenland | Cirrhosis due to hepatitis C | 1.53 | | 2.37 | 55.11 | 1.59 |
| Greenland | Cirrhosis due to alcohol use | 1.34 | | 2.23 | 66.35 | 1.85 |
| Greenland | Cirrhosis due to other causes | 0.82 | | 1.21 | 47.12 | 1.39 |
| Greenland | Cirrhosis due to NASH | 0.39 | | 0.67 | 70.40 | 1.93 |
| Grenada | Cirrhosis | 14.53 | | 18.65 | 28.35 | 0.02 |
| Grenada | Cirrhosis due to hepatitis B | 1.78 | | 1.93 | 8.25 | -0.61 |
| Grenada | Cirrhosis due to hepatitis C | 3.19 | | 4.15 | 30.07 | 0.07 |
| Grenada | Cirrhosis due to alcohol use | 5.30 | | 6.59 | 24.23 | -0.10 |
| Grenada | Cirrhosis due to other causes | 1.97 | | 2.31 | 17.13 | -0.32 |
| Grenada | Cirrhosis due to NASH | 2.28 | | 3.67 | 60.86 | 0.85 |
| Guam | Cirrhosis | 20.77 | | 34.49 | 66.03 | 1.12 |
| Guam | Cirrhosis due to hepatitis B | 9.40 | | 14.74 | 56.77 | 0.91 |
| Guam | Cirrhosis due to hepatitis C | 4.62 | | 7.76 | 67.77 | 1.16 |
| Guam | Cirrhosis due to alcohol use | 3.44 | | 5.87 | 70.71 | 1.23 |
| Guam | Cirrhosis due to other causes | 1.53 | | 2.08 | 36.30 | 0.39 |
| Guam | Cirrhosis due to NASH | 1.78 | | 4.04 | 126.78 | 2.28 |
| Guatemala | Cirrhosis | 2063.98 | | 4623.29 | 124.00 | 0.22 |
| Guatemala | Cirrhosis due to hepatitis B | 175.98 | | 353.46 | 100.86 | -0.19 |
| Guatemala | Cirrhosis due to hepatitis C | 660.79 | | 1536.29 | 132.49 | 0.35 |
| Guatemala | Cirrhosis due to alcohol use | 666.57 | | 1541.68 | 131.28 | 0.33 |
| Guatemala | Cirrhosis due to other causes | 321.45 | | 485.26 | 50.96 | -1.25 |
| Guatemala | Cirrhosis due to NASH | 239.19 | | 706.61 | 195.41 | 1.24 |
| Guinea | Cirrhosis | 1369.68 | | 1688.58 | 23.28 | -1.65 |
| Guinea | Cirrhosis due to hepatitis B | 737.73 | | 911.49 | 23.55 | -1.64 |
| Guinea | Cirrhosis due to hepatitis C | 103.40 | | 130.78 | 26.47 | -1.55 |
| Guinea | Cirrhosis due to alcohol use | 198.56 | | 263.73 | 32.82 | -1.37 |
| Guinea | Cirrhosis due to other causes | 260.46 | | 286.50 | 10.00 | -2.07 |
| Guinea | Cirrhosis due to NASH | 69.52 | | 96.07 | 38.19 | -1.22 |
| Guinea-Bissau | Cirrhosis | 211.38 | | 290.34 | 37.36 | -1.08 |
| Guinea-Bissau | Cirrhosis due to hepatitis B | 114.28 | | 156.46 | 36.91 | -1.09 |
| Guinea-Bissau | Cirrhosis due to hepatitis C | 15.84 | | 21.65 | 36.66 | -1.10 |
| Guinea-Bissau | Cirrhosis due to alcohol use | 33.45 | | 46.76 | 39.81 | -1.01 |
| Guinea-Bissau | Cirrhosis due to other causes | 36.48 | | 46.23 | 26.73 | -1.38 |
| Guinea-Bissau | Cirrhosis due to NASH | 11.32 | | 19.23 | 69.83 | -0.29 |
| Guyana | Cirrhosis | 165.76 | | 173.67 | 4.77 | 0.35 |
| Guyana | Cirrhosis due to hepatitis B | 21.86 | | 18.69 | -14.49 | -0.40 |
| Guyana | Cirrhosis due to hepatitis C | 37.70 | | 40.39 | 7.14 | 0.44 |
| Guyana | Cirrhosis due to alcohol use | 61.47 | | 62.43 | 1.57 | 0.24 |
| Guyana | Cirrhosis due to other causes | 20.11 | | 18.28 | -9.13 | -0.17 |
| Guyana | Cirrhosis due to NASH | 24.62 | | 33.88 | 37.59 | 1.36 |
| Haiti | Cirrhosis | 1004.05 | | 1656.33 | 64.97 | -0.43 |
| Haiti | Cirrhosis due to hepatitis B | 126.11 | | 207.31 | 64.39 | -0.45 |
| Haiti | Cirrhosis due to hepatitis C | 231.28 | | 391.88 | 69.44 | -0.33 |
| Haiti | Cirrhosis due to alcohol use | 342.13 | | 581.41 | 69.94 | -0.32 |
| Haiti | Cirrhosis due to other causes | 166.52 | | 212.42 | 27.56 | -1.39 |
| Haiti | Cirrhosis due to NASH | 138.01 | | 263.31 | 90.80 | 0.11 |
| Honduras | Cirrhosis | 981.67 | | 2388.38 | 143.30 | 0.69 |
| Honduras | Cirrhosis due to hepatitis B | 80.48 | | 168.09 | 108.85 | 0.13 |
| Honduras | Cirrhosis due to hepatitis C | 300.50 | | 770.23 | 156.32 | 0.89 |
| Honduras | Cirrhosis due to alcohol use | 291.99 | | 784.51 | 168.68 | 1.06 |
| Honduras | Cirrhosis due to other causes | 193.76 | | 271.60 | 40.17 | -1.35 |
| Honduras | Cirrhosis due to NASH | 114.94 | | 393.96 | 242.75 | 1.96 |
| Hungary | Cirrhosis | 5941.34 | | 3227.81 | -45.67 | -1.99 |
| Hungary | Cirrhosis due to hepatitis B | 1469.34 | | 791.09 | -46.16 | -2.03 |
| Hungary | Cirrhosis due to hepatitis C | 1136.22 | | 627.70 | -44.76 | -1.93 |
| Hungary | Cirrhosis due to alcohol use | 2669.42 | | 1403.12 | -47.44 | -2.11 |
| Hungary | Cirrhosis due to other causes | 291.63 | | 172.66 | -40.79 | -1.67 |
| Hungary | Cirrhosis due to NASH | 374.72 | | 233.23 | -37.76 | -1.49 |
| Iceland | Cirrhosis | 12.19 | | 19.05 | 56.32 | 0.60 |
| Iceland | Cirrhosis due to hepatitis B | 0.94 | | 1.42 | 51.07 | 0.47 |
| Iceland | Cirrhosis due to hepatitis C | 1.00 | | 1.45 | 44.92 | 0.32 |
| Iceland | Cirrhosis due to alcohol use | 4.74 | | 7.56 | 59.43 | 0.67 |
| Iceland | Cirrhosis due to other causes | 3.69 | | 5.36 | 45.19 | 0.32 |
| Iceland | Cirrhosis due to NASH | 1.81 | | 3.26 | 79.88 | 1.12 |
| India | Cirrhosis | 110090.59 | | 217895.56 | 97.92 | 0.82 |
| India | Cirrhosis due to hepatitis B | 37316.56 | | 75602.68 | 102.60 | 0.91 |
| India | Cirrhosis due to hepatitis C | 17910.89 | | 36299.89 | 102.67 | 0.91 |
| India | Cirrhosis due to alcohol use | 33225.18 | | 72040.02 | 116.82 | 1.16 |
| India | Cirrhosis due to other causes | 16876.06 | | 22663.27 | 34.29 | -0.61 |
| India | Cirrhosis due to NASH | 4761.91 | | 11289.70 | 137.08 | 1.49 |
| Indonesia | Cirrhosis | 58847.33 | | 82144.53 | 39.59 | 0.02 |
| Indonesia | Cirrhosis due to hepatitis B | 17651.68 | | 23532.21 | 33.31 | -0.15 |
| Indonesia | Cirrhosis due to hepatitis C | 24126.08 | | 34444.23 | 42.77 | 0.10 |
| Indonesia | Cirrhosis due to alcohol use | 6691.17 | | 9763.48 | 45.92 | 0.18 |
| Indonesia | Cirrhosis due to other causes | 5668.66 | | 6144.32 | 8.39 | -0.92 |
| Indonesia | Cirrhosis due to NASH | 4709.74 | | 8260.28 | 75.39 | 0.86 |
| Iran | Cirrhosis | 2723.85 | | 5399.93 | 98.25 | 1.24 |
| Iran | Cirrhosis due to hepatitis B | 810.01 | | 1549.42 | 91.28 | 1.10 |
| Iran | Cirrhosis due to hepatitis C | 1057.17 | | 2340.52 | 121.40 | 1.64 |
| Iran | Cirrhosis due to alcohol use | 131.02 | | 290.90 | 122.03 | 1.66 |
| Iran | Cirrhosis due to other causes | 482.35 | | 472.31 | -2.08 | -1.38 |
| Iran | Cirrhosis due to NASH | 243.31 | | 746.77 | 206.92 | 2.85 |
| Iraq | Cirrhosis | 966.12 | | 1185.07 | 22.66 | -2.61 |
| Iraq | Cirrhosis due to hepatitis B | 323.23 | | 367.73 | 13.77 | -2.89 |
| Iraq | Cirrhosis due to hepatitis C | 338.94 | | 431.94 | 27.44 | -2.47 |
| Iraq | Cirrhosis due to alcohol use | 54.30 | | 69.31 | 27.64 | -2.46 |
| Iraq | Cirrhosis due to other causes | 144.42 | | 174.10 | 20.55 | -2.68 |
| Iraq | Cirrhosis due to NASH | 105.22 | | 142.00 | 34.96 | -2.26 |
| Ireland | Cirrhosis | 189.60 | | 392.54 | 107.04 | 1.58 |
| Ireland | Cirrhosis due to hepatitis B | 17.19 | | 34.31 | 99.53 | 1.45 |
| Ireland | Cirrhosis due to hepatitis C | 34.53 | | 69.87 | 102.32 | 1.50 |
| Ireland | Cirrhosis due to alcohol use | 80.67 | | 173.08 | 114.55 | 1.71 |
| Ireland | Cirrhosis due to other causes | 40.65 | | 74.64 | 83.61 | 1.14 |
| Ireland | Cirrhosis due to NASH | 16.55 | | 40.65 | 145.60 | 2.22 |
| Israel | Cirrhosis | 393.38 | | 707.31 | 79.81 | -0.01 |
| Israel | Cirrhosis due to hepatitis B | 40.05 | | 61.23 | 52.89 | -0.61 |
| Israel | Cirrhosis due to hepatitis C | 77.97 | | 140.18 | 79.80 | -0.01 |
| Israel | Cirrhosis due to alcohol use | 135.08 | | 232.70 | 72.27 | -0.17 |
| Israel | Cirrhosis due to other causes | 101.66 | | 189.39 | 86.29 | 0.12 |
| Israel | Cirrhosis due to NASH | 38.62 | | 83.81 | 117.02 | 0.69 |
| Italy | Cirrhosis | 17687.88 | | 11695.34 | -33.88 | -1.77 |
| Italy | Cirrhosis due to hepatitis B | 2122.60 | | 1268.45 | -40.24 | -2.15 |
| Italy | Cirrhosis due to hepatitis C | 8223.70 | | 5667.58 | -31.08 | -1.62 |
| Italy | Cirrhosis due to alcohol use | 3886.74 | | 2174.29 | -44.06 | -2.39 |
| Italy | Cirrhosis due to other causes | 2559.12 | | 1885.90 | -26.31 | -1.37 |
| Italy | Cirrhosis due to NASH | 895.73 | | 699.11 | -21.95 | -1.16 |
| Jamaica | Cirrhosis | 161.17 | | 191.98 | 19.11 | 0.06 |
| Jamaica | Cirrhosis due to hepatitis B | 19.81 | | 20.46 | 3.30 | -0.47 |
| Jamaica | Cirrhosis due to hepatitis C | 35.19 | | 43.52 | 23.68 | 0.20 |
| Jamaica | Cirrhosis due to alcohol use | 49.82 | | 61.60 | 23.63 | 0.20 |
| Jamaica | Cirrhosis due to other causes | 30.43 | | 27.14 | -10.83 | -1.01 |
| Jamaica | Cirrhosis due to NASH | 25.91 | | 39.25 | 51.47 | 0.95 |
| Japan | Cirrhosis | 22683.82 | | 25351.93 | 11.76 | 0.34 |
| Japan | Cirrhosis due to hepatitis B | 3485.90 | | 4021.14 | 15.35 | 0.46 |
| Japan | Cirrhosis due to hepatitis C | 12389.13 | | 13630.37 | 10.02 | 0.28 |
| Japan | Cirrhosis due to alcohol use | 3671.31 | | 3842.24 | 4.66 | 0.10 |
| Japan | Cirrhosis due to other causes | 2100.49 | | 2692.72 | 28.20 | 0.85 |
| Japan | Cirrhosis due to NASH | 1037.00 | | 1165.46 | 12.39 | 0.36 |
| Jordan | Cirrhosis | 194.77 | | 444.77 | 128.36 | -0.82 |
| Jordan | Cirrhosis due to hepatitis B | 65.69 | | 134.35 | 104.52 | -1.23 |
| Jordan | Cirrhosis due to hepatitis C | 69.20 | | 169.53 | 144.99 | -0.56 |
| Jordan | Cirrhosis due to alcohol use | 10.59 | | 27.19 | 156.90 | -0.38 |
| Jordan | Cirrhosis due to other causes | 28.44 | | 45.06 | 58.43 | -2.17 |
| Jordan | Cirrhosis due to NASH | 20.85 | | 68.64 | 229.14 | 0.54 |
| Kazakhstan | Cirrhosis | 2171.43 | | 6848.52 | 215.39 | 4.03 |
| Kazakhstan | Cirrhosis due to hepatitis B | 502.17 | | 1408.77 | 180.54 | 3.59 |
| Kazakhstan | Cirrhosis due to hepatitis C | 496.00 | | 1629.30 | 228.49 | 4.18 |
| Kazakhstan | Cirrhosis due to alcohol use | 805.42 | | 2708.05 | 236.23 | 4.26 |
| Kazakhstan | Cirrhosis due to other causes | 212.10 | | 551.12 | 159.83 | 3.31 |
| Kazakhstan | Cirrhosis due to NASH | 155.74 | | 551.27 | 253.97 | 4.46 |
| Kenya | Cirrhosis | 5272.98 | | 10398.41 | 97.20 | -0.20 |
| Kenya | Cirrhosis due to hepatitis B | 1613.20 | | 2883.83 | 78.76 | -0.57 |
| Kenya | Cirrhosis due to hepatitis C | 654.49 | | 1408.81 | 115.25 | 0.12 |
| Kenya | Cirrhosis due to alcohol use | 1379.69 | | 2854.40 | 106.89 | -0.03 |
| Kenya | Cirrhosis due to other causes | 1166.83 | | 2208.28 | 89.26 | -0.36 |
| Kenya | Cirrhosis due to NASH | 458.76 | | 1043.09 | 127.37 | 0.32 |
| Kiribati | Cirrhosis | 12.38 | | 18.05 | 45.79 | -0.31 |
| Kiribati | Cirrhosis due to hepatitis B | 5.91 | | 7.73 | 30.82 | -0.71 |
| Kiribati | Cirrhosis due to hepatitis C | 2.66 | | 4.14 | 55.54 | -0.07 |
| Kiribati | Cirrhosis due to alcohol use | 1.64 | | 2.56 | 55.43 | -0.07 |
| Kiribati | Cirrhosis due to other causes | 1.14 | | 1.62 | 41.82 | -0.41 |
| Kiribati | Cirrhosis due to NASH | 1.03 | | 2.01 | 95.55 | 0.78 |
| Kuwait | Cirrhosis | 54.02 | | 158.80 | 193.99 | 0.75 |
| Kuwait | Cirrhosis due to hepatitis B | 19.26 | | 48.09 | 149.66 | 0.14 |
| Kuwait | Cirrhosis due to hepatitis C | 20.63 | | 62.66 | 203.70 | 0.87 |
| Kuwait | Cirrhosis due to alcohol use | 3.42 | | 10.56 | 209.00 | 0.93 |
| Kuwait | Cirrhosis due to other causes | 4.25 | | 9.29 | 118.41 | -0.35 |
| Kuwait | Cirrhosis due to NASH | 6.45 | | 28.20 | 337.20 | 2.22 |
| Kyrgyzstan | Cirrhosis | 1060.11 | | 2158.98 | 103.66 | 1.32 |
| Kyrgyzstan | Cirrhosis due to hepatitis B | 263.31 | | 493.84 | 87.55 | 1.01 |
| Kyrgyzstan | Cirrhosis due to hepatitis C | 248.79 | | 542.18 | 117.93 | 1.57 |
| Kyrgyzstan | Cirrhosis due to alcohol use | 363.38 | | 777.24 | 113.89 | 1.50 |
| Kyrgyzstan | Cirrhosis due to other causes | 113.42 | | 180.40 | 59.05 | 0.40 |
| Kyrgyzstan | Cirrhosis due to NASH | 71.21 | | 165.31 | 132.15 | 1.80 |
| Laos | Cirrhosis | 877.20 | | 1595.23 | 81.86 | 0.28 |
| Laos | Cirrhosis due to hepatitis B | 348.36 | | 601.09 | 72.55 | 0.09 |
| Laos | Cirrhosis due to hepatitis C | 232.65 | | 441.76 | 89.88 | 0.44 |
| Laos | Cirrhosis due to alcohol use | 154.29 | | 287.04 | 86.03 | 0.37 |
| Laos | Cirrhosis due to other causes | 76.94 | | 124.58 | 61.91 | -0.15 |
| Laos | Cirrhosis due to NASH | 64.95 | | 140.77 | 116.73 | 0.93 |
| Latvia | Cirrhosis | 244.28 | | 434.30 | 77.79 | 3.37 |
| Latvia | Cirrhosis due to hepatitis B | 48.00 | | 72.90 | 51.86 | 2.79 |
| Latvia | Cirrhosis due to hepatitis C | 56.48 | | 101.37 | 79.46 | 3.41 |
| Latvia | Cirrhosis due to alcohol use | 91.70 | | 174.14 | 89.90 | 3.61 |
| Latvia | Cirrhosis due to other causes | 23.93 | | 37.98 | 58.73 | 2.95 |
| Latvia | Cirrhosis due to NASH | 24.16 | | 47.91 | 98.28 | 3.77 |
| Lebanon | Cirrhosis | 298.45 | | 518.65 | 73.78 | -0.65 |
| Lebanon | Cirrhosis due to hepatitis B | 111.44 | | 178.67 | 60.33 | -0.95 |
| Lebanon | Cirrhosis due to hepatitis C | 107.49 | | 189.05 | 75.88 | -0.61 |
| Lebanon | Cirrhosis due to alcohol use | 18.45 | | 30.70 | 66.36 | -0.81 |
| Lebanon | Cirrhosis due to other causes | 32.30 | | 50.52 | 56.39 | -1.04 |
| Lebanon | Cirrhosis due to NASH | 28.77 | | 69.72 | 142.37 | 0.58 |
| Lesotho | Cirrhosis | 183.31 | | 208.89 | 13.95 | 0.21 |
| Lesotho | Cirrhosis due to hepatitis B | 49.73 | | 47.56 | -4.36 | -0.44 |
| Lesotho | Cirrhosis due to hepatitis C | 63.23 | | 74.63 | 18.02 | 0.34 |
| Lesotho | Cirrhosis due to alcohol use | 34.19 | | 40.56 | 18.64 | 0.36 |
| Lesotho | Cirrhosis due to other causes | 24.69 | | 29.59 | 19.84 | 0.39 |
| Lesotho | Cirrhosis due to NASH | 11.48 | | 16.56 | 44.28 | 1.08 |
| Liberia | Cirrhosis | 650.88 | | 732.55 | 12.55 | -2.77 |
| Liberia | Cirrhosis due to hepatitis B | 339.09 | | 389.06 | 14.74 | -2.70 |
| Liberia | Cirrhosis due to hepatitis C | 48.49 | | 53.44 | 10.21 | -2.85 |
| Liberia | Cirrhosis due to alcohol use | 111.49 | | 118.12 | 5.95 | -3.00 |
| Liberia | Cirrhosis due to other causes | 114.25 | | 122.80 | 7.49 | -2.94 |
| Liberia | Cirrhosis due to NASH | 37.56 | | 49.13 | 30.79 | -2.22 |
| Libya | Cirrhosis | 308.92 | | 568.03 | 83.87 | 0.40 |
| Libya | Cirrhosis due to hepatitis B | 105.20 | | 170.39 | 61.97 | -0.07 |
| Libya | Cirrhosis due to hepatitis C | 111.26 | | 214.17 | 92.49 | 0.57 |
| Libya | Cirrhosis due to alcohol use | 17.52 | | 33.87 | 93.31 | 0.58 |
| Libya | Cirrhosis due to other causes | 39.40 | | 56.07 | 42.32 | -0.55 |
| Libya | Cirrhosis due to NASH | 35.55 | | 93.53 | 163.13 | 1.73 |
| Lithuania | Cirrhosis | 351.49 | | 942.04 | 168.01 | 4.67 |
| Lithuania | Cirrhosis due to hepatitis B | 70.95 | | 157.90 | 122.55 | 3.98 |
| Lithuania | Cirrhosis due to hepatitis C | 81.80 | | 217.50 | 165.88 | 4.64 |
| Lithuania | Cirrhosis due to alcohol use | 134.93 | | 393.20 | 191.40 | 4.98 |
| Lithuania | Cirrhosis due to other causes | 31.78 | | 74.79 | 135.33 | 4.19 |
| Lithuania | Cirrhosis due to NASH | 32.02 | | 98.66 | 208.08 | 5.19 |
| Luxembourg | Cirrhosis | 93.45 | | 99.20 | 6.16 | -1.34 |
| Luxembourg | Cirrhosis due to hepatitis B | 8.41 | | 7.27 | -13.48 | -2.10 |
| Luxembourg | Cirrhosis due to hepatitis C | 17.03 | | 17.10 | 0.39 | -1.54 |
| Luxembourg | Cirrhosis due to alcohol use | 42.46 | | 46.21 | 8.85 | -1.25 |
| Luxembourg | Cirrhosis due to other causes | 17.63 | | 18.85 | 6.94 | -1.31 |
| Luxembourg | Cirrhosis due to NASH | 7.92 | | 9.76 | 23.24 | -0.79 |
| Macedonia | Cirrhosis | 152.28 | | 235.21 | 54.46 | 1.32 |
| Macedonia | Cirrhosis due to hepatitis B | 42.94 | | 58.81 | 36.97 | 0.87 |
| Macedonia | Cirrhosis due to hepatitis C | 32.44 | | 52.69 | 62.42 | 1.51 |
| Macedonia | Cirrhosis due to alcohol use | 55.89 | | 88.68 | 58.66 | 1.42 |
| Macedonia | Cirrhosis due to other causes | 11.11 | | 15.51 | 39.64 | 0.95 |
| Macedonia | Cirrhosis due to NASH | 9.90 | | 19.51 | 97.11 | 2.22 |
| Madagascar | Cirrhosis | 2049.59 | | 2939.20 | 43.40 | -1.56 |
| Madagascar | Cirrhosis due to hepatitis B | 557.45 | | 731.44 | 31.21 | -1.89 |
| Madagascar | Cirrhosis due to hepatitis C | 595.20 | | 929.24 | 56.12 | -1.24 |
| Madagascar | Cirrhosis due to alcohol use | 371.13 | | 569.81 | 53.54 | -1.30 |
| Madagascar | Cirrhosis due to other causes | 408.87 | | 517.94 | 26.68 | -2.02 |
| Madagascar | Cirrhosis due to NASH | 116.96 | | 190.76 | 63.11 | -1.08 |
| Malawi | Cirrhosis | 2327.91 | | 2639.70 | 13.39 | -1.67 |
| Malawi | Cirrhosis due to hepatitis B | 656.23 | | 669.29 | 1.99 | -2.06 |
| Malawi | Cirrhosis due to hepatitis C | 686.89 | | 805.48 | 17.26 | -1.54 |
| Malawi | Cirrhosis due to alcohol use | 411.62 | | 481.54 | 16.99 | -1.55 |
| Malawi | Cirrhosis due to other causes | 426.07 | | 500.27 | 17.41 | -1.54 |
| Malawi | Cirrhosis due to NASH | 147.10 | | 183.11 | 24.48 | -1.32 |
| Malaysia | Cirrhosis | 1476.71 | | 3082.27 | 108.72 | 0.68 |
| Malaysia | Cirrhosis due to hepatitis B | 708.35 | | 1346.42 | 90.08 | 0.33 |
| Malaysia | Cirrhosis due to hepatitis C | 276.02 | | 594.18 | 115.26 | 0.79 |
| Malaysia | Cirrhosis due to alcohol use | 260.16 | | 554.18 | 113.01 | 0.76 |
| Malaysia | Cirrhosis due to other causes | 96.71 | | 193.03 | 99.61 | 0.52 |
| Malaysia | Cirrhosis due to NASH | 135.47 | | 394.46 | 191.19 | 1.91 |
| Maldives | Cirrhosis | 10.20 | | 18.06 | 77.18 | -0.60 |
| Maldives | Cirrhosis due to hepatitis B | 3.94 | | 6.30 | 59.66 | -0.99 |
| Maldives | Cirrhosis due to hepatitis C | 2.79 | | 5.18 | 86.00 | -0.42 |
| Maldives | Cirrhosis due to alcohol use | 1.61 | | 3.01 | 86.47 | -0.41 |
| Maldives | Cirrhosis due to other causes | 0.97 | | 1.33 | 37.01 | -1.56 |
| Maldives | Cirrhosis due to NASH | 0.88 | | 2.25 | 155.04 | 0.75 |
| Mali | Cirrhosis | 1826.22 | | 1783.98 | -2.31 | -3.23 |
| Mali | Cirrhosis due to hepatitis B | 997.02 | | 923.16 | -7.41 | -3.43 |
| Mali | Cirrhosis due to hepatitis C | 140.76 | | 132.77 | -5.68 | -3.36 |
| Mali | Cirrhosis due to alcohol use | 240.07 | | 233.34 | -2.80 | -3.25 |
| Mali | Cirrhosis due to other causes | 357.91 | | 393.19 | 9.86 | -2.80 |
| Mali | Cirrhosis due to NASH | 90.46 | | 101.52 | 12.23 | -2.72 |
| Malta | Cirrhosis | 34.82 | | 43.71 | 25.50 | 0.24 |
| Malta | Cirrhosis due to hepatitis B | 3.27 | | 3.60 | 10.11 | -0.24 |
| Malta | Cirrhosis due to hepatitis C | 6.54 | | 7.96 | 21.81 | 0.13 |
| Malta | Cirrhosis due to alcohol use | 14.77 | | 17.87 | 20.98 | 0.11 |
| Malta | Cirrhosis due to other causes | 6.86 | | 9.00 | 31.11 | 0.40 |
| Malta | Cirrhosis due to NASH | 3.38 | | 5.27 | 55.92 | 1.05 |
| Marshall Islands | Cirrhosis | 6.18 | | 8.89 | 43.96 | 0.58 |
| Marshall Islands | Cirrhosis due to hepatitis B | 2.89 | | 3.79 | 31.29 | 0.24 |
| Marshall Islands | Cirrhosis due to hepatitis C | 1.30 | | 2.05 | 57.42 | 0.91 |
| Marshall Islands | Cirrhosis due to alcohol use | 0.85 | | 1.38 | 62.40 | 1.03 |
| Marshall Islands | Cirrhosis due to other causes | 0.64 | | 0.67 | 5.53 | -0.57 |
| Marshall Islands | Cirrhosis due to NASH | 0.50 | | 1.00 | 99.85 | 1.80 |
| Mauritania | Cirrhosis | 372.39 | | 425.51 | 14.26 | -1.86 |
| Mauritania | Cirrhosis due to hepatitis B | 201.31 | | 221.12 | 9.84 | -2.01 |
| Mauritania | Cirrhosis due to hepatitis C | 30.42 | | 35.32 | 16.11 | -1.80 |
| Mauritania | Cirrhosis due to alcohol use | 51.85 | | 62.22 | 20.01 | -1.68 |
| Mauritania | Cirrhosis due to other causes | 64.95 | | 71.96 | 10.79 | -1.98 |
| Mauritania | Cirrhosis due to NASH | 23.86 | | 34.89 | 46.25 | -0.95 |
| Mauritius | Cirrhosis | 244.55 | | 238.22 | -2.59 | -0.64 |
| Mauritius | Cirrhosis due to hepatitis B | 96.81 | | 82.21 | -15.08 | -1.15 |
| Mauritius | Cirrhosis due to hepatitis C | 68.27 | | 69.32 | 1.54 | -0.49 |
| Mauritius | Cirrhosis due to alcohol use | 45.78 | | 45.34 | -0.96 | -0.58 |
| Mauritius | Cirrhosis due to other causes | 15.15 | | 14.23 | -6.03 | -0.77 |
| Mauritius | Cirrhosis due to NASH | 18.54 | | 27.12 | 46.24 | 0.86 |
| Mexico | Cirrhosis | 23103.77 | | 40508.90 | 75.33 | 0.62 |
| Mexico | Cirrhosis due to hepatitis B | 1632.59 | | 2419.67 | 48.21 | 0.00 |
| Mexico | Cirrhosis due to hepatitis C | 7575.56 | | 12719.78 | 67.91 | 0.46 |
| Mexico | Cirrhosis due to alcohol use | 8620.42 | | 14810.77 | 71.81 | 0.55 |
| Mexico | Cirrhosis due to other causes | 2360.32 | | 4011.79 | 69.97 | 0.51 |
| Mexico | Cirrhosis due to NASH | 2914.89 | | 6546.88 | 124.60 | 1.54 |
| Moldova | Cirrhosis | 3186.86 | | 3094.02 | -2.91 | 0.56 |
| Moldova | Cirrhosis due to hepatitis B | 639.69 | | 554.57 | -13.31 | 0.14 |
| Moldova | Cirrhosis due to hepatitis C | 768.40 | | 769.86 | 0.19 | 0.68 |
| Moldova | Cirrhosis due to alcohol use | 1133.92 | | 1096.97 | -3.26 | 0.55 |
| Moldova | Cirrhosis due to other causes | 324.68 | | 308.54 | -4.97 | 0.48 |
| Moldova | Cirrhosis due to NASH | 320.17 | | 364.08 | 13.71 | 1.15 |
| Mongolia | Cirrhosis | 717.11 | | 1374.37 | 91.65 | 0.88 |
| Mongolia | Cirrhosis due to hepatitis B | 176.79 | | 301.58 | 70.59 | 0.45 |
| Mongolia | Cirrhosis due to hepatitis C | 163.48 | | 341.44 | 108.85 | 1.20 |
| Mongolia | Cirrhosis due to alcohol use | 226.11 | | 503.04 | 122.48 | 1.43 |
| Mongolia | Cirrhosis due to other causes | 106.81 | | 123.03 | 15.18 | -1.00 |
| Mongolia | Cirrhosis due to NASH | 43.92 | | 105.27 | 139.67 | 1.71 |
| Montenegro | Cirrhosis | 37.46 | | 49.00 | 30.80 | 0.99 |
| Montenegro | Cirrhosis due to hepatitis B | 10.45 | | 11.78 | 12.78 | 0.44 |
| Montenegro | Cirrhosis due to hepatitis C | 7.97 | | 10.66 | 33.75 | 1.07 |
| Montenegro | Cirrhosis due to alcohol use | 14.41 | | 20.05 | 39.12 | 1.22 |
| Montenegro | Cirrhosis due to other causes | 2.20 | | 2.91 | 32.13 | 1.03 |
| Montenegro | Cirrhosis due to NASH | 2.42 | | 3.59 | 48.18 | 1.45 |
| Morocco | Cirrhosis | 2047.44 | | 3298.02 | 61.08 | 0.50 |
| Morocco | Cirrhosis due to hepatitis B | 725.25 | | 1044.50 | 44.02 | 0.08 |
| Morocco | Cirrhosis due to hepatitis C | 749.60 | | 1300.31 | 73.47 | 0.77 |
| Morocco | Cirrhosis due to alcohol use | 112.67 | | 197.40 | 75.20 | 0.81 |
| Morocco | Cirrhosis due to other causes | 275.71 | | 328.63 | 19.20 | -0.62 |
| Morocco | Cirrhosis due to NASH | 184.21 | | 427.17 | 131.89 | 1.85 |
| Mozambique | Cirrhosis | 1932.97 | | 2380.05 | 23.13 | -1.95 |
| Mozambique | Cirrhosis due to hepatitis B | 558.09 | | 614.69 | 10.14 | -2.36 |
| Mozambique | Cirrhosis due to hepatitis C | 515.57 | | 652.79 | 26.62 | -1.85 |
| Mozambique | Cirrhosis due to alcohol use | 338.86 | | 437.94 | 29.24 | -1.77 |
| Mozambique | Cirrhosis due to other causes | 404.41 | | 517.34 | 27.92 | -1.81 |
| Mozambique | Cirrhosis due to NASH | 116.04 | | 157.29 | 35.55 | -1.60 |
| Myanmar | Cirrhosis | 16886.67 | | 23171.37 | 37.22 | 0.18 |
| Myanmar | Cirrhosis due to hepatitis B | 7084.90 | | 8820.59 | 24.50 | -0.18 |
| Myanmar | Cirrhosis due to hepatitis C | 4733.68 | | 7034.48 | 48.61 | 0.48 |
| Myanmar | Cirrhosis due to alcohol use | 2427.83 | | 3708.46 | 52.75 | 0.58 |
| Myanmar | Cirrhosis due to other causes | 1525.95 | | 1672.73 | 9.62 | -0.65 |
| Myanmar | Cirrhosis due to NASH | 1114.32 | | 1935.12 | 73.66 | 1.06 |
| Namibia | Cirrhosis | 93.56 | | 216.59 | 131.50 | 1.23 |
| Namibia | Cirrhosis due to hepatitis B | 23.03 | | 51.71 | 124.48 | 1.11 |
| Namibia | Cirrhosis due to hepatitis C | 33.09 | | 75.09 | 126.96 | 1.15 |
| Namibia | Cirrhosis due to alcohol use | 16.63 | | 45.52 | 173.76 | 1.85 |
| Namibia | Cirrhosis due to other causes | 15.37 | | 30.08 | 95.70 | 0.60 |
| Namibia | Cirrhosis due to NASH | 5.44 | | 14.20 | 160.80 | 1.67 |
| Nepal | Cirrhosis | 3139.35 | | 5671.17 | 80.65 | 0.58 |
| Nepal | Cirrhosis due to hepatitis B | 821.87 | | 1281.05 | 55.87 | 0.04 |
| Nepal | Cirrhosis due to hepatitis C | 456.44 | | 848.58 | 85.91 | 0.69 |
| Nepal | Cirrhosis due to alcohol use | 1278.50 | | 2650.01 | 107.27 | 1.09 |
| Nepal | Cirrhosis due to other causes | 464.64 | | 608.59 | 30.98 | -0.61 |
| Nepal | Cirrhosis due to NASH | 117.90 | | 282.94 | 139.99 | 1.64 |
| Netherlands | Cirrhosis | 1382.23 | | 1680.56 | 21.58 | 0.23 |
| Netherlands | Cirrhosis due to hepatitis B | 123.93 | | 141.41 | 14.11 | 0.00 |
| Netherlands | Cirrhosis due to hepatitis C | 241.60 | | 287.98 | 19.19 | 0.16 |
| Netherlands | Cirrhosis due to alcohol use | 571.63 | | 672.62 | 17.67 | 0.11 |
| Netherlands | Cirrhosis due to other causes | 325.52 | | 400.85 | 23.14 | 0.28 |
| Netherlands | Cirrhosis due to NASH | 119.55 | | 177.70 | 48.65 | 0.98 |
| New Zealand | Cirrhosis | 194.90 | | 292.14 | 49.89 | 0.52 |
| New Zealand | Cirrhosis due to hepatitis B | 38.42 | | 50.56 | 31.59 | 0.04 |
| New Zealand | Cirrhosis due to hepatitis C | 69.00 | | 104.39 | 51.29 | 0.56 |
| New Zealand | Cirrhosis due to alcohol use | 54.61 | | 79.41 | 45.42 | 0.41 |
| New Zealand | Cirrhosis due to other causes | 14.08 | | 22.00 | 56.23 | 0.68 |
| New Zealand | Cirrhosis due to NASH | 18.78 | | 35.77 | 90.45 | 1.41 |
| Nicaragua | Cirrhosis | 427.06 | | 1126.54 | 163.79 | 1.75 |
| Nicaragua | Cirrhosis due to hepatitis B | 34.04 | | 74.78 | 119.66 | 1.08 |
| Nicaragua | Cirrhosis due to hepatitis C | 132.60 | | 347.74 | 162.25 | 1.73 |
| Nicaragua | Cirrhosis due to alcohol use | 150.99 | | 401.78 | 166.09 | 1.79 |
| Nicaragua | Cirrhosis due to other causes | 53.51 | | 98.68 | 84.41 | 0.43 |
| Nicaragua | Cirrhosis due to NASH | 55.91 | | 203.55 | 264.06 | 2.95 |
| Niger | Cirrhosis | 1067.67 | | 1503.90 | 40.86 | -2.36 |
| Niger | Cirrhosis due to hepatitis B | 562.01 | | 808.51 | 43.86 | -2.28 |
| Niger | Cirrhosis due to hepatitis C | 75.12 | | 107.17 | 42.67 | -2.31 |
| Niger | Cirrhosis due to alcohol use | 142.01 | | 207.21 | 45.91 | -2.23 |
| Niger | Cirrhosis due to other causes | 241.65 | | 304.70 | 26.09 | -2.77 |
| Niger | Cirrhosis due to NASH | 46.88 | | 76.32 | 62.80 | -1.82 |
| Nigeria | Cirrhosis | 33925.54 | | 45473.58 | 34.04 | -1.99 |
| Nigeria | Cirrhosis due to hepatitis B | 16270.85 | | 21601.16 | 32.76 | -2.03 |
| Nigeria | Cirrhosis due to hepatitis C | 2742.14 | | 3539.48 | 29.08 | -2.13 |
| Nigeria | Cirrhosis due to alcohol use | 7900.46 | | 9890.52 | 25.19 | -2.25 |
| Nigeria | Cirrhosis due to other causes | 5170.69 | | 7708.36 | 49.08 | -1.60 |
| Nigeria | Cirrhosis due to NASH | 1841.39 | | 2734.06 | 48.48 | -1.61 |
| North Korea | Cirrhosis | 2879.39 | | 4657.17 | 61.74 | 0.90 |
| North Korea | Cirrhosis due to hepatitis B | 1558.00 | | 2329.17 | 49.50 | 0.61 |
| North Korea | Cirrhosis due to hepatitis C | 625.15 | | 1097.13 | 75.50 | 1.21 |
| North Korea | Cirrhosis due to alcohol use | 452.67 | | 783.50 | 73.08 | 1.16 |
| North Korea | Cirrhosis due to other causes | 112.75 | | 205.19 | 81.99 | 1.34 |
| North Korea | Cirrhosis due to NASH | 130.82 | | 242.19 | 85.13 | 1.40 |
| Northern Mariana Islands | Cirrhosis | 5.21 | | 8.66 | 66.19 | 1.91 |
| Northern Mariana Islands | Cirrhosis due to hepatitis B | 2.42 | | 3.74 | 54.79 | 1.64 |
| Northern Mariana Islands | Cirrhosis due to hepatitis C | 1.19 | | 2.01 | 68.37 | 1.95 |
| Northern Mariana Islands | Cirrhosis due to alcohol use | 0.82 | | 1.42 | 74.24 | 2.08 |
| Northern Mariana Islands | Cirrhosis due to other causes | 0.33 | | 0.50 | 51.69 | 1.57 |
| Northern Mariana Islands | Cirrhosis due to NASH | 0.46 | | 0.99 | 116.79 | 2.89 |
| Norway | Cirrhosis | 344.94 | | 372.62 | 8.02 | -0.52 |
| Norway | Cirrhosis due to hepatitis B | 30.95 | | 32.26 | 4.24 | -0.65 |
| Norway | Cirrhosis due to hepatitis C | 62.55 | | 66.21 | 5.86 | -0.60 |
| Norway | Cirrhosis due to alcohol use | 147.15 | | 157.38 | 6.95 | -0.56 |
| Norway | Cirrhosis due to other causes | 75.25 | | 80.35 | 6.77 | -0.56 |
| Norway | Cirrhosis due to NASH | 29.04 | | 36.41 | 25.39 | 0.03 |
| Oman | Cirrhosis | 89.21 | | 176.29 | 97.63 | -0.67 |
| Oman | Cirrhosis due to hepatitis B | 32.02 | | 54.17 | 69.16 | -1.24 |
| Oman | Cirrhosis due to hepatitis C | 33.10 | | 69.66 | 110.41 | -0.43 |
| Oman | Cirrhosis due to alcohol use | 5.24 | | 11.68 | 122.85 | -0.22 |
| Oman | Cirrhosis due to other causes | 10.87 | | 14.00 | 28.77 | -2.25 |
| Oman | Cirrhosis due to NASH | 7.97 | | 26.79 | 236.25 | 1.30 |
| Pakistan | Cirrhosis | 23817.38 | | 45501.13 | 91.04 | -0.12 |
| Pakistan | Cirrhosis due to hepatitis B | 6891.50 | | 11998.03 | 74.10 | -0.47 |
| Pakistan | Cirrhosis due to hepatitis C | 8954.50 | | 17974.43 | 100.73 | 0.06 |
| Pakistan | Cirrhosis due to alcohol use | 2379.31 | | 4725.40 | 98.60 | 0.02 |
| Pakistan | Cirrhosis due to other causes | 4343.52 | | 7924.07 | 82.43 | -0.29 |
| Pakistan | Cirrhosis due to NASH | 1248.54 | | 2879.20 | 130.61 | 0.57 |
| Palestine | Cirrhosis | 117.67 | | 221.67 | 88.38 | -0.87 |
| Palestine | Cirrhosis due to hepatitis B | 39.70 | | 69.18 | 74.24 | -1.16 |
| Palestine | Cirrhosis due to hepatitis C | 40.46 | | 84.73 | 109.40 | -0.48 |
| Palestine | Cirrhosis due to alcohol use | 6.14 | | 13.16 | 114.29 | -0.39 |
| Palestine | Cirrhosis due to other causes | 20.08 | | 25.73 | 28.18 | -2.30 |
| Palestine | Cirrhosis due to NASH | 11.29 | | 28.86 | 155.71 | 0.26 |
| Panama | Cirrhosis | 203.05 | | 455.17 | 124.17 | 1.15 |
| Panama | Cirrhosis due to hepatitis B | 15.64 | | 31.19 | 99.40 | 0.72 |
| Panama | Cirrhosis due to hepatitis C | 61.30 | | 137.83 | 124.84 | 1.16 |
| Panama | Cirrhosis due to alcohol use | 74.58 | | 172.69 | 131.56 | 1.27 |
| Panama | Cirrhosis due to other causes | 27.77 | | 49.62 | 78.71 | 0.31 |
| Panama | Cirrhosis due to NASH | 23.76 | | 63.83 | 168.68 | 1.82 |
| Papua New Guinea | Cirrhosis | 666.52 | | 1306.56 | 96.03 | -0.54 |
| Papua New Guinea | Cirrhosis due to hepatitis B | 318.50 | | 577.81 | 81.42 | -0.83 |
| Papua New Guinea | Cirrhosis due to hepatitis C | 149.55 | | 314.38 | 110.22 | -0.28 |
| Papua New Guinea | Cirrhosis due to alcohol use | 102.27 | | 211.79 | 107.08 | -0.34 |
| Papua New Guinea | Cirrhosis due to other causes | 57.87 | | 114.83 | 98.43 | -0.50 |
| Papua New Guinea | Cirrhosis due to NASH | 38.33 | | 87.75 | 128.94 | 0.03 |
| Paraguay | Cirrhosis | 321.07 | | 670.94 | 108.97 | 0.72 |
| Paraguay | Cirrhosis due to hepatitis B | 57.38 | | 108.16 | 88.50 | 0.34 |
| Paraguay | Cirrhosis due to hepatitis C | 71.85 | | 156.45 | 117.74 | 0.88 |
| Paraguay | Cirrhosis due to alcohol use | 84.42 | | 179.55 | 112.67 | 0.79 |
| Paraguay | Cirrhosis due to other causes | 56.82 | | 89.34 | 57.24 | -0.33 |
| Paraguay | Cirrhosis due to NASH | 50.60 | | 137.44 | 171.63 | 1.69 |
| Peru | Cirrhosis | 3498.43 | | 5561.87 | 58.98 | 0.17 |
| Peru | Cirrhosis due to hepatitis B | 438.70 | | 641.46 | 46.22 | -0.14 |
| Peru | Cirrhosis due to hepatitis C | 424.72 | | 729.33 | 71.72 | 0.46 |
| Peru | Cirrhosis due to alcohol use | 1437.64 | | 2324.84 | 61.71 | 0.23 |
| Peru | Cirrhosis due to other causes | 703.86 | | 744.55 | 5.78 | -1.34 |
| Peru | Cirrhosis due to NASH | 493.50 | | 1121.69 | 127.29 | 1.49 |
| Philippines | Cirrhosis | 3796.24 | | 12696.36 | 234.45 | 2.65 |
| Philippines | Cirrhosis due to hepatitis B | 1554.47 | | 4600.86 | 195.98 | 2.20 |
| Philippines | Cirrhosis due to hepatitis C | 1025.08 | | 3659.95 | 257.04 | 2.90 |
| Philippines | Cirrhosis due to alcohol use | 679.62 | | 2377.86 | 249.88 | 2.82 |
| Philippines | Cirrhosis due to other causes | 284.01 | | 905.43 | 218.81 | 2.48 |
| Philippines | Cirrhosis due to NASH | 253.07 | | 1152.25 | 355.31 | 3.80 |
| Poland | Cirrhosis | 4827.36 | | 7788.44 | 61.34 | 1.84 |
| Poland | Cirrhosis due to hepatitis B | 1221.17 | | 1691.95 | 38.55 | 1.27 |
| Poland | Cirrhosis due to hepatitis C | 939.09 | | 1588.64 | 69.17 | 2.01 |
| Poland | Cirrhosis due to alcohol use | 2075.55 | | 3514.47 | 69.33 | 2.01 |
| Poland | Cirrhosis due to other causes | 315.82 | | 441.45 | 39.78 | 1.30 |
| Poland | Cirrhosis due to NASH | 275.74 | | 551.92 | 100.16 | 2.63 |
| Portugal | Cirrhosis | 3541.34 | | 2132.96 | -39.77 | -2.08 |
| Portugal | Cirrhosis due to hepatitis B | 315.16 | | 170.32 | -45.96 | -2.48 |
| Portugal | Cirrhosis due to hepatitis C | 625.09 | | 392.62 | -37.19 | -1.92 |
| Portugal | Cirrhosis due to alcohol use | 1701.35 | | 937.40 | -44.90 | -2.41 |
| Portugal | Cirrhosis due to other causes | 619.34 | | 413.71 | -33.20 | -1.69 |
| Portugal | Cirrhosis due to NASH | 280.41 | | 218.90 | -21.93 | -1.12 |
| Puerto Rico | Cirrhosis | 1017.77 | | 952.99 | -6.36 | -0.30 |
| Puerto Rico | Cirrhosis due to hepatitis B | 129.72 | | 95.83 | -26.13 | -1.18 |
| Puerto Rico | Cirrhosis due to hepatitis C | 230.79 | | 206.50 | -10.52 | -0.47 |
| Puerto Rico | Cirrhosis due to alcohol use | 372.64 | | 324.98 | -12.79 | -0.56 |
| Puerto Rico | Cirrhosis due to other causes | 108.96 | | 116.91 | 7.29 | 0.21 |
| Puerto Rico | Cirrhosis due to NASH | 175.66 | | 208.78 | 18.85 | 0.59 |
| Qatar | Cirrhosis | 26.88 | | 113.54 | 322.34 | -1.42 |
| Qatar | Cirrhosis due to hepatitis B | 9.22 | | 34.90 | 278.47 | -1.83 |
| Qatar | Cirrhosis due to hepatitis C | 9.80 | | 44.61 | 355.00 | -1.14 |
| Qatar | Cirrhosis due to alcohol use | 1.73 | | 8.04 | 365.87 | -1.06 |
| Qatar | Cirrhosis due to other causes | 2.50 | | 6.90 | 176.31 | -2.99 |
| Qatar | Cirrhosis due to NASH | 3.64 | | 19.10 | 425.12 | -0.61 |
| Romania | Cirrhosis | 8378.69 | | 10114.34 | 20.72 | 1.38 |
| Romania | Cirrhosis due to hepatitis B | 2064.82 | | 2199.04 | 6.50 | 0.92 |
| Romania | Cirrhosis due to hepatitis C | 1613.13 | | 2065.95 | 28.07 | 1.60 |
| Romania | Cirrhosis due to alcohol use | 3669.71 | | 4448.75 | 21.23 | 1.40 |
| Romania | Cirrhosis due to other causes | 554.34 | | 657.35 | 18.58 | 1.32 |
| Romania | Cirrhosis due to NASH | 476.68 | | 743.25 | 55.92 | 2.33 |
| Russian Federation | Cirrhosis | 17451.39 | | 50909.93 | 191.72 | 4.09 |
| Russian Federation | Cirrhosis due to hepatitis B | 3635.28 | | 9198.88 | 153.04 | 3.57 |
| Russian Federation | Cirrhosis due to hepatitis C | 4230.66 | | 12551.63 | 196.68 | 4.15 |
| Russian Federation | Cirrhosis due to alcohol use | 6039.76 | | 18683.27 | 209.34 | 4.31 |
| Russian Federation | Cirrhosis due to other causes | 1853.75 | | 4768.50 | 157.24 | 3.63 |
| Russian Federation | Cirrhosis due to NASH | 1691.94 | | 5707.67 | 237.34 | 4.63 |
| Rwanda | Cirrhosis | 2715.94 | | 2668.40 | -1.75 | -2.09 |
| Rwanda | Cirrhosis due to hepatitis B | 431.15 | | 377.11 | -12.53 | -2.52 |
| Rwanda | Cirrhosis due to hepatitis C | 1236.09 | | 1256.35 | 1.64 | -1.96 |
| Rwanda | Cirrhosis due to alcohol use | 523.26 | | 505.33 | -3.43 | -2.15 |
| Rwanda | Cirrhosis due to other causes | 392.66 | | 383.54 | -2.32 | -2.11 |
| Rwanda | Cirrhosis due to NASH | 132.78 | | 146.07 | 10.01 | -1.67 |
| Saint Lucia | Cirrhosis | 21.84 | | 27.53 | 26.03 | -0.09 |
| Saint Lucia | Cirrhosis due to hepatitis B | 2.57 | | 2.75 | 6.97 | -0.69 |
| Saint Lucia | Cirrhosis due to hepatitis C | 4.70 | | 6.02 | 27.97 | -0.03 |
| Saint Lucia | Cirrhosis due to alcohol use | 7.82 | | 9.54 | 21.99 | -0.21 |
| Saint Lucia | Cirrhosis due to other causes | 3.02 | | 3.23 | 7.18 | -0.69 |
| Saint Lucia | Cirrhosis due to NASH | 3.73 | | 5.98 | 60.52 | 0.81 |
| Saint Vincent and the Grenadines | Cirrhosis | 11.72 | | 16.93 | 44.40 | 1.23 |
| Saint Vincent and the Grenadines | Cirrhosis due to hepatitis B | 1.46 | | 1.72 | 18.18 | 0.49 |
| Saint Vincent and the Grenadines | Cirrhosis due to hepatitis C | 2.58 | | 3.63 | 40.34 | 1.13 |
| Saint Vincent and the Grenadines | Cirrhosis due to alcohol use | 4.05 | | 5.79 | 42.96 | 1.20 |
| Saint Vincent and the Grenadines | Cirrhosis due to other causes | 1.51 | | 1.63 | 8.14 | 0.16 |
| Saint Vincent and the Grenadines | Cirrhosis due to NASH | 2.12 | | 4.16 | 95.96 | 2.36 |
| Samoa | Cirrhosis | 19.16 | | 22.88 | 19.40 | -0.06 |
| Samoa | Cirrhosis due to hepatitis B | 8.80 | | 9.51 | 8.13 | -0.43 |
| Samoa | Cirrhosis due to hepatitis C | 4.05 | | 5.15 | 27.28 | 0.18 |
| Samoa | Cirrhosis due to alcohol use | 2.70 | | 3.48 | 28.70 | 0.22 |
| Samoa | Cirrhosis due to other causes | 1.79 | | 1.81 | 1.07 | -0.68 |
| Samoa | Cirrhosis due to NASH | 1.82 | | 2.92 | 60.57 | 1.04 |
| Sao Tome and Principe | Cirrhosis | 51.29 | | 79.20 | 54.44 | -0.23 |
| Sao Tome and Principe | Cirrhosis due to hepatitis B | 26.61 | | 39.96 | 50.13 | -0.33 |
| Sao Tome and Principe | Cirrhosis due to hepatitis C | 3.90 | | 6.51 | 66.89 | 0.06 |
| Sao Tome and Principe | Cirrhosis due to alcohol use | 7.93 | | 14.71 | 85.47 | 0.45 |
| Sao Tome and Principe | Cirrhosis due to other causes | 10.01 | | 12.60 | 25.86 | -0.98 |
| Sao Tome and Principe | Cirrhosis due to NASH | 2.83 | | 5.43 | 91.96 | 0.58 |
| Saudi Arabia | Cirrhosis | 1575.48 | | 2461.03 | 56.21 | -1.10 |
| Saudi Arabia | Cirrhosis due to hepatitis B | 417.93 | | 548.59 | 31.26 | -1.74 |
| Saudi Arabia | Cirrhosis due to hepatitis C | 737.08 | | 1196.96 | 62.39 | -0.96 |
| Saudi Arabia | Cirrhosis due to alcohol use | 92.74 | | 156.37 | 68.62 | -0.82 |
| Saudi Arabia | Cirrhosis due to other causes | 159.53 | | 157.38 | -1.35 | -2.80 |
| Saudi Arabia | Cirrhosis due to NASH | 168.20 | | 401.73 | 138.84 | 0.47 |
| Senegal | Cirrhosis | 980.98 | | 1421.12 | 44.87 | -1.06 |
| Senegal | Cirrhosis due to hepatitis B | 544.14 | | 775.93 | 42.60 | -1.11 |
| Senegal | Cirrhosis due to hepatitis C | 71.08 | | 112.91 | 58.86 | -0.71 |
| Senegal | Cirrhosis due to alcohol use | 123.29 | | 201.51 | 63.44 | -0.61 |
| Senegal | Cirrhosis due to other causes | 192.23 | | 239.29 | 24.49 | -1.62 |
| Senegal | Cirrhosis due to NASH | 50.25 | | 91.48 | 82.05 | -0.21 |
| Serbia | Cirrhosis | 1520.34 | | 1304.07 | -14.23 | -0.35 |
| Serbia | Cirrhosis due to hepatitis B | 413.91 | | 301.44 | -27.17 | -0.96 |
| Serbia | Cirrhosis due to hepatitis C | 332.23 | | 281.84 | -15.17 | -0.40 |
| Serbia | Cirrhosis due to alcohol use | 586.71 | | 540.71 | -7.84 | -0.09 |
| Serbia | Cirrhosis due to other causes | 88.03 | | 82.30 | -6.51 | -0.04 |
| Serbia | Cirrhosis due to NASH | 99.47 | | 97.78 | -1.69 | 0.15 |
| Seychelles | Cirrhosis | 13.47 | | 27.50 | 104.16 | 1.44 |
| Seychelles | Cirrhosis due to hepatitis B | 5.28 | | 9.41 | 78.33 | 0.94 |
| Seychelles | Cirrhosis due to hepatitis C | 3.78 | | 7.99 | 111.05 | 1.57 |
| Seychelles | Cirrhosis due to alcohol use | 2.23 | | 4.80 | 115.18 | 1.64 |
| Seychelles | Cirrhosis due to other causes | 0.84 | | 1.63 | 94.12 | 1.26 |
| Seychelles | Cirrhosis due to NASH | 1.34 | | 3.67 | 174.42 | 2.54 |
| Sierra Leone | Cirrhosis | 1102.56 | | 1086.08 | -1.49 | -2.75 |
| Sierra Leone | Cirrhosis due to hepatitis B | 610.91 | | 555.85 | -9.01 | -3.04 |
| Sierra Leone | Cirrhosis due to hepatitis C | 78.72 | | 85.14 | 8.16 | -2.40 |
| Sierra Leone | Cirrhosis due to alcohol use | 174.85 | | 197.30 | 12.84 | -2.25 |
| Sierra Leone | Cirrhosis due to other causes | 184.39 | | 180.84 | -1.93 | -2.77 |
| Sierra Leone | Cirrhosis due to NASH | 53.69 | | 66.94 | 24.69 | -1.88 |
| Singapore | Cirrhosis | 193.30 | | 254.15 | 31.48 | -1.07 |
| Singapore | Cirrhosis due to hepatitis B | 111.10 | | 137.65 | 23.90 | -1.29 |
| Singapore | Cirrhosis due to hepatitis C | 17.83 | | 24.70 | 38.50 | -0.87 |
| Singapore | Cirrhosis due to alcohol use | 30.90 | | 42.12 | 36.31 | -0.93 |
| Singapore | Cirrhosis due to other causes | 29.50 | | 42.79 | 45.05 | -0.70 |
| Singapore | Cirrhosis due to NASH | 3.97 | | 6.90 | 73.72 | -0.03 |
| Slovakia | Cirrhosis | 1654.23 | | 1526.88 | -7.70 | -0.40 |
| Slovakia | Cirrhosis due to hepatitis B | 414.82 | | 331.58 | -20.07 | -0.93 |
| Slovakia | Cirrhosis due to hepatitis C | 317.60 | | 310.24 | -2.32 | -0.19 |
| Slovakia | Cirrhosis due to alcohol use | 739.74 | | 692.19 | -6.43 | -0.34 |
| Slovakia | Cirrhosis due to other causes | 85.17 | | 84.38 | -0.93 | -0.13 |
| Slovakia | Cirrhosis due to NASH | 96.90 | | 108.51 | 11.97 | 0.32 |
| Slovenia | Cirrhosis | 787.13 | | 541.20 | -31.24 | -1.53 |
| Slovenia | Cirrhosis due to hepatitis B | 196.70 | | 122.45 | -37.75 | -1.90 |
| Slovenia | Cirrhosis due to hepatitis C | 155.34 | | 112.43 | -27.62 | -1.34 |
| Slovenia | Cirrhosis due to alcohol use | 345.06 | | 231.05 | -33.04 | -1.63 |
| Slovenia | Cirrhosis due to other causes | 42.07 | | 32.89 | -21.82 | -1.05 |
| Slovenia | Cirrhosis due to NASH | 47.96 | | 42.38 | -11.64 | -0.60 |
| Solomon Islands | Cirrhosis | 40.75 | | 74.85 | 83.69 | -0.10 |
| Solomon Islands | Cirrhosis due to hepatitis B | 19.63 | | 32.41 | 65.11 | -0.49 |
| Solomon Islands | Cirrhosis due to hepatitis C | 9.10 | | 18.06 | 98.42 | 0.19 |
| Solomon Islands | Cirrhosis due to alcohol use | 5.91 | | 11.78 | 99.31 | 0.20 |
| Solomon Islands | Cirrhosis due to other causes | 3.25 | | 5.87 | 80.78 | -0.16 |
| Solomon Islands | Cirrhosis due to NASH | 2.86 | | 6.72 | 135.54 | 0.82 |
| Somalia | Cirrhosis | 961.22 | | 2521.95 | 162.37 | 0.40 |
| Somalia | Cirrhosis due to hepatitis B | 251.32 | | 661.42 | 163.18 | 0.42 |
| Somalia | Cirrhosis due to hepatitis C | 296.49 | | 773.43 | 160.86 | 0.38 |
| Somalia | Cirrhosis due to alcohol use | 183.58 | | 478.93 | 160.88 | 0.38 |
| Somalia | Cirrhosis due to other causes | 167.34 | | 437.49 | 161.44 | 0.39 |
| Somalia | Cirrhosis due to NASH | 62.49 | | 170.67 | 173.14 | 0.55 |
| South Africa | Cirrhosis | 4167.62 | | 4604.68 | 10.49 | -1.12 |
| South Africa | Cirrhosis due to hepatitis B | 1058.55 | | 969.49 | -8.41 | -1.81 |
| South Africa | Cirrhosis due to hepatitis C | 1278.84 | | 1538.11 | 20.27 | -0.80 |
| South Africa | Cirrhosis due to alcohol use | 851.48 | | 941.96 | 10.63 | -1.11 |
| South Africa | Cirrhosis due to other causes | 642.63 | | 668.78 | 4.07 | -1.34 |
| South Africa | Cirrhosis due to NASH | 336.12 | | 486.34 | 44.69 | -0.12 |
| South Korea | Cirrhosis | 15083.04 | | 9447.36 | -37.36 | -2.38 |
| South Korea | Cirrhosis due to hepatitis B | 5774.47 | | 3281.67 | -43.17 | -2.74 |
| South Korea | Cirrhosis due to hepatitis C | 1263.27 | | 830.60 | -34.25 | -2.20 |
| South Korea | Cirrhosis due to alcohol use | 5946.01 | | 3863.69 | -35.02 | -2.24 |
| South Korea | Cirrhosis due to other causes | 1451.70 | | 981.41 | -32.40 | -2.09 |
| South Korea | Cirrhosis due to NASH | 647.58 | | 489.98 | -24.34 | -1.68 |
| South Sudan | Cirrhosis | 835.62 | | 1397.31 | 67.22 | -0.04 |
| South Sudan | Cirrhosis due to hepatitis B | 230.63 | | 381.75 | 65.52 | -0.08 |
| South Sudan | Cirrhosis due to hepatitis C | 244.56 | | 410.04 | 67.66 | -0.03 |
| South Sudan | Cirrhosis due to alcohol use | 161.14 | | 271.85 | 68.71 | -0.01 |
| South Sudan | Cirrhosis due to other causes | 141.24 | | 232.08 | 64.32 | -0.10 |
| South Sudan | Cirrhosis due to NASH | 58.05 | | 101.59 | 74.99 | 0.13 |
| Spain | Cirrhosis | 10690.39 | | 8132.42 | -23.93 | -1.59 |
| Spain | Cirrhosis due to hepatitis B | 938.31 | | 617.93 | -34.14 | -2.13 |
| Spain | Cirrhosis due to hepatitis C | 2097.68 | | 1598.49 | -23.80 | -1.59 |
| Spain | Cirrhosis due to alcohol use | 5881.83 | | 4244.78 | -27.83 | -1.79 |
| Spain | Cirrhosis due to other causes | 805.25 | | 724.70 | -10.00 | -0.97 |
| Spain | Cirrhosis due to NASH | 967.32 | | 946.53 | -2.15 | -0.66 |
| Sri Lanka | Cirrhosis | 3431.08 | | 4020.92 | 17.19 | -0.26 |
| Sri Lanka | Cirrhosis due to hepatitis B | 1404.87 | | 1448.26 | 3.09 | -0.73 |
| Sri Lanka | Cirrhosis due to hepatitis C | 990.32 | | 1223.14 | 23.51 | -0.07 |
| Sri Lanka | Cirrhosis due to alcohol use | 555.95 | | 713.53 | 28.34 | 0.08 |
| Sri Lanka | Cirrhosis due to other causes | 243.06 | | 268.41 | 10.43 | -0.48 |
| Sri Lanka | Cirrhosis due to NASH | 236.88 | | 367.58 | 55.17 | 0.78 |
| Sudan | Cirrhosis | 2247.53 | | 3147.59 | 40.05 | -1.30 |
| Sudan | Cirrhosis due to hepatitis B | 792.29 | | 1037.14 | 30.90 | -1.55 |
| Sudan | Cirrhosis due to hepatitis C | 776.78 | | 1133.29 | 45.90 | -1.15 |
| Sudan | Cirrhosis due to alcohol use | 132.47 | | 198.73 | 50.02 | -1.05 |
| Sudan | Cirrhosis due to other causes | 361.96 | | 443.94 | 22.65 | -1.80 |
| Sudan | Cirrhosis due to NASH | 184.03 | | 334.48 | 81.75 | -0.34 |
| Suriname | Cirrhosis | 70.50 | | 123.55 | 75.23 | 0.64 |
| Suriname | Cirrhosis due to hepatitis B | 8.87 | | 13.68 | 54.14 | 0.16 |
| Suriname | Cirrhosis due to hepatitis C | 16.06 | | 27.56 | 71.57 | 0.56 |
| Suriname | Cirrhosis due to alcohol use | 23.90 | | 43.34 | 81.31 | 0.77 |
| Suriname | Cirrhosis due to other causes | 10.40 | | 14.63 | 40.62 | -0.18 |
| Suriname | Cirrhosis due to NASH | 11.26 | | 24.34 | 116.15 | 1.42 |
| Swaziland | Cirrhosis | 103.80 | | 126.72 | 22.08 | -0.49 |
| Swaziland | Cirrhosis due to hepatitis B | 27.14 | | 29.08 | 7.14 | -0.97 |
| Swaziland | Cirrhosis due to hepatitis C | 35.87 | | 45.70 | 27.40 | -0.33 |
| Swaziland | Cirrhosis due to alcohol use | 19.04 | | 23.10 | 21.28 | -0.51 |
| Swaziland | Cirrhosis due to other causes | 14.38 | | 17.91 | 24.52 | -0.41 |
| Swaziland | Cirrhosis due to NASH | 7.36 | | 10.94 | 48.61 | 0.24 |
| Sweden | Cirrhosis | 827.43 | | 1070.52 | 29.38 | 0.37 |
| Sweden | Cirrhosis due to hepatitis B | 72.14 | | 92.99 | 28.89 | 0.35 |
| Sweden | Cirrhosis due to hepatitis C | 138.99 | | 174.40 | 25.48 | 0.25 |
| Sweden | Cirrhosis due to alcohol use | 380.18 | | 449.38 | 18.20 | 0.03 |
| Sweden | Cirrhosis due to other causes | 162.80 | | 236.46 | 45.24 | 0.80 |
| Sweden | Cirrhosis due to NASH | 73.31 | | 117.29 | 60.01 | 1.16 |
| Switzerland | Cirrhosis | 805.95 | | 939.85 | 16.61 | -0.21 |
| Switzerland | Cirrhosis due to hepatitis B | 71.07 | | 82.42 | 15.97 | -0.23 |
| Switzerland | Cirrhosis due to hepatitis C | 143.03 | | 166.35 | 16.30 | -0.22 |
| Switzerland | Cirrhosis due to alcohol use | 381.00 | | 406.02 | 6.57 | -0.54 |
| Switzerland | Cirrhosis due to other causes | 148.74 | | 199.34 | 34.02 | 0.31 |
| Switzerland | Cirrhosis due to NASH | 62.11 | | 85.73 | 38.05 | 0.42 |
| Syria | Cirrhosis | 845.84 | | 1525.33 | 80.33 | 0.86 |
| Syria | Cirrhosis due to hepatitis B | 286.87 | | 483.97 | 68.71 | 0.61 |
| Syria | Cirrhosis due to hepatitis C | 297.93 | | 588.14 | 97.41 | 1.20 |
| Syria | Cirrhosis due to alcohol use | 44.72 | | 92.94 | 107.84 | 1.39 |
| Syria | Cirrhosis due to other causes | 135.03 | | 156.55 | 15.94 | -0.77 |
| Syria | Cirrhosis due to NASH | 81.30 | | 203.73 | 150.61 | 2.08 |
| Taiwan (Province of China) | Cirrhosis | 5241.50 | | 6509.59 | 24.19 | 0.27 |
| Taiwan (Province of China) | Cirrhosis due to hepatitis B | 2660.10 | | 2927.99 | 10.07 | -0.18 |
| Taiwan (Province of China) | Cirrhosis due to hepatitis C | 1244.21 | | 1663.93 | 33.73 | 0.54 |
| Taiwan (Province of China) | Cirrhosis due to alcohol use | 869.17 | | 1188.87 | 36.78 | 0.62 |
| Taiwan (Province of China) | Cirrhosis due to other causes | 187.97 | | 261.12 | 38.92 | 0.68 |
| Taiwan (Province of China) | Cirrhosis due to NASH | 280.04 | | 467.68 | 67.00 | 1.36 |
| Tajikistan | Cirrhosis | 817.94 | | 1536.23 | 87.82 | 0.33 |
| Tajikistan | Cirrhosis due to hepatitis B | 180.66 | | 333.78 | 84.76 | 0.27 |
| Tajikistan | Cirrhosis due to hepatitis C | 169.36 | | 359.72 | 112.40 | 0.78 |
| Tajikistan | Cirrhosis due to alcohol use | 253.85 | | 517.47 | 103.85 | 0.63 |
| Tajikistan | Cirrhosis due to other causes | 167.76 | | 218.43 | 30.20 | -1.03 |
| Tajikistan | Cirrhosis due to NASH | 46.31 | | 106.83 | 130.67 | 1.09 |
| Tanzania | Cirrhosis | 4541.53 | | 6161.39 | 35.67 | -1.59 |
| Tanzania | Cirrhosis due to hepatitis B | 1137.97 | | 1408.82 | 23.80 | -1.93 |
| Tanzania | Cirrhosis due to hepatitis C | 1315.81 | | 1822.89 | 38.54 | -1.51 |
| Tanzania | Cirrhosis due to alcohol use | 934.32 | | 1237.57 | 32.46 | -1.68 |
| Tanzania | Cirrhosis due to other causes | 853.47 | | 1243.26 | 45.67 | -1.33 |
| Tanzania | Cirrhosis due to NASH | 299.96 | | 448.86 | 49.64 | -1.23 |
| Thailand | Cirrhosis | 9366.68 | | 17238.74 | 84.04 | 1.47 |
| Thailand | Cirrhosis due to hepatitis B | 3549.13 | | 5899.17 | 66.21 | 1.09 |
| Thailand | Cirrhosis due to hepatitis C | 2466.19 | | 4706.64 | 90.85 | 1.60 |
| Thailand | Cirrhosis due to alcohol use | 1531.16 | | 3028.56 | 97.79 | 1.73 |
| Thailand | Cirrhosis due to other causes | 815.45 | | 1219.10 | 49.50 | 0.70 |
| Thailand | Cirrhosis due to NASH | 1004.76 | | 2385.28 | 137.40 | 2.41 |
| The Bahamas | Cirrhosis | 38.39 | | 53.72 | 39.92 | -0.15 |
| The Bahamas | Cirrhosis due to hepatitis B | 4.62 | | 5.56 | 20.20 | -0.72 |
| The Bahamas | Cirrhosis due to hepatitis C | 8.32 | | 12.14 | 45.88 | 0.00 |
| The Bahamas | Cirrhosis due to alcohol use | 14.80 | | 19.59 | 32.38 | -0.36 |
| The Bahamas | Cirrhosis due to other causes | 4.50 | | 5.99 | 33.02 | -0.34 |
| The Bahamas | Cirrhosis due to NASH | 6.14 | | 10.43 | 69.94 | 0.57 |
| The Gambia | Cirrhosis | 132.42 | | 284.44 | 114.80 | -0.02 |
| The Gambia | Cirrhosis due to hepatitis B | 70.71 | | 146.55 | 107.24 | -0.16 |
| The Gambia | Cirrhosis due to hepatitis C | 9.82 | | 22.76 | 131.68 | 0.26 |
| The Gambia | Cirrhosis due to alcohol use | 20.76 | | 48.90 | 135.60 | 0.32 |
| The Gambia | Cirrhosis due to other causes | 24.10 | | 47.41 | 96.77 | -0.35 |
| The Gambia | Cirrhosis due to NASH | 7.03 | | 18.82 | 167.76 | 0.79 |
| Timor-Leste | Cirrhosis | 85.50 | | 227.36 | 165.92 | 1.77 |
| Timor-Leste | Cirrhosis due to hepatitis B | 34.07 | | 86.00 | 152.45 | 1.58 |
| Timor-Leste | Cirrhosis due to hepatitis C | 23.61 | | 66.12 | 180.01 | 1.96 |
| Timor-Leste | Cirrhosis due to alcohol use | 12.34 | | 35.14 | 184.74 | 2.03 |
| Timor-Leste | Cirrhosis due to other causes | 9.20 | | 19.72 | 114.40 | 0.97 |
| Timor-Leste | Cirrhosis due to NASH | 6.28 | | 20.38 | 224.48 | 2.51 |
| Togo | Cirrhosis | 597.01 | | 747.36 | 25.18 | -1.81 |
| Togo | Cirrhosis due to hepatitis B | 319.58 | | 408.99 | 27.98 | -1.73 |
| Togo | Cirrhosis due to hepatitis C | 45.45 | | 59.32 | 30.50 | -1.65 |
| Togo | Cirrhosis due to alcohol use | 88.60 | | 117.44 | 32.55 | -1.60 |
| Togo | Cirrhosis due to other causes | 114.08 | | 115.14 | 0.93 | -2.60 |
| Togo | Cirrhosis due to NASH | 29.29 | | 46.47 | 58.66 | -0.93 |
| Tonga | Cirrhosis | 15.20 | | 20.05 | 31.96 | 0.79 |
| Tonga | Cirrhosis due to hepatitis B | 6.86 | | 8.00 | 16.59 | 0.33 |
| Tonga | Cirrhosis due to hepatitis C | 3.23 | | 4.54 | 40.63 | 1.03 |
| Tonga | Cirrhosis due to alcohol use | 2.08 | | 2.98 | 43.19 | 1.09 |
| Tonga | Cirrhosis due to other causes | 1.42 | | 1.72 | 21.51 | 0.48 |
| Tonga | Cirrhosis due to NASH | 1.61 | | 2.81 | 74.90 | 1.83 |
| Trinidad and Tobago | Cirrhosis | 149.41 | | 181.33 | 21.36 | 0.19 |
| Trinidad and Tobago | Cirrhosis due to hepatitis B | 19.46 | | 19.94 | 2.45 | -0.44 |
| Trinidad and Tobago | Cirrhosis due to hepatitis C | 34.18 | | 40.87 | 19.55 | 0.13 |
| Trinidad and Tobago | Cirrhosis due to alcohol use | 50.45 | | 60.10 | 19.14 | 0.12 |
| Trinidad and Tobago | Cirrhosis due to other causes | 19.20 | | 19.94 | 3.87 | -0.39 |
| Trinidad and Tobago | Cirrhosis due to NASH | 26.13 | | 40.49 | 54.97 | 1.09 |
| Tunisia | Cirrhosis | 547.57 | | 1000.50 | 82.72 | 1.09 |
| Tunisia | Cirrhosis due to hepatitis B | 76.45 | | 119.95 | 56.89 | 0.53 |
| Tunisia | Cirrhosis due to hepatitis C | 293.54 | | 557.46 | 89.91 | 1.24 |
| Tunisia | Cirrhosis due to alcohol use | 37.54 | | 72.23 | 92.41 | 1.28 |
| Tunisia | Cirrhosis due to other causes | 80.54 | | 101.44 | 25.94 | -0.29 |
| Tunisia | Cirrhosis due to NASH | 59.50 | | 149.43 | 151.14 | 2.27 |
| Turkey | Cirrhosis | 3404.44 | | 5023.64 | 47.56 | 0.21 |
| Turkey | Cirrhosis due to hepatitis B | 1863.71 | | 2745.77 | 47.33 | 0.20 |
| Turkey | Cirrhosis due to hepatitis C | 507.71 | | 812.84 | 60.10 | 0.51 |
| Turkey | Cirrhosis due to alcohol use | 199.28 | | 336.22 | 68.72 | 0.70 |
| Turkey | Cirrhosis due to other causes | 493.39 | | 419.86 | -14.90 | -1.83 |
| Turkey | Cirrhosis due to NASH | 340.35 | | 708.95 | 108.30 | 1.49 |
| Turkmenistan | Cirrhosis | 841.60 | | 2341.30 | 178.19 | 2.69 |
| Turkmenistan | Cirrhosis due to hepatitis B | 207.48 | | 526.22 | 153.62 | 2.35 |
| Turkmenistan | Cirrhosis due to hepatitis C | 191.02 | | 567.21 | 196.94 | 2.93 |
| Turkmenistan | Cirrhosis due to alcohol use | 274.32 | | 838.99 | 205.84 | 3.04 |
| Turkmenistan | Cirrhosis due to other causes | 115.01 | | 219.54 | 90.90 | 1.30 |
| Turkmenistan | Cirrhosis due to NASH | 53.78 | | 189.33 | 252.08 | 3.57 |
| Uganda | Cirrhosis | 2724.24 | | 4316.00 | 58.43 | -1.30 |
| Uganda | Cirrhosis due to hepatitis B | 668.15 | | 988.04 | 47.88 | -1.56 |
| Uganda | Cirrhosis due to hepatitis C | 780.36 | | 1272.08 | 63.01 | -1.20 |
| Uganda | Cirrhosis due to alcohol use | 618.63 | | 991.19 | 60.22 | -1.26 |
| Uganda | Cirrhosis due to other causes | 489.01 | | 780.65 | 59.64 | -1.28 |
| Uganda | Cirrhosis due to NASH | 168.09 | | 284.04 | 68.98 | -1.06 |
| Ukraine | Cirrhosis | 8324.46 | | 20082.89 | 141.25 | 3.87 |
| Ukraine | Cirrhosis due to hepatitis B | 1700.95 | | 3755.77 | 120.80 | 3.54 |
| Ukraine | Cirrhosis due to hepatitis C | 1989.53 | | 4823.30 | 142.43 | 3.89 |
| Ukraine | Cirrhosis due to alcohol use | 3030.66 | | 7734.94 | 155.22 | 4.08 |
| Ukraine | Cirrhosis due to other causes | 807.19 | | 1642.27 | 103.45 | 3.24 |
| Ukraine | Cirrhosis due to NASH | 796.14 | | 2126.62 | 167.12 | 4.25 |
| United Arab Emirates | Cirrhosis | 75.98 | | 412.74 | 443.20 | 0.19 |
| United Arab Emirates | Cirrhosis due to hepatitis B | 27.35 | | 130.84 | 378.35 | -0.28 |
| United Arab Emirates | Cirrhosis due to hepatitis C | 30.41 | | 171.96 | 465.55 | 0.34 |
| United Arab Emirates | Cirrhosis due to alcohol use | 6.09 | | 32.58 | 434.87 | 0.14 |
| United Arab Emirates | Cirrhosis due to other causes | 3.79 | | 15.79 | 316.48 | -0.79 |
| United Arab Emirates | Cirrhosis due to NASH | 8.34 | | 61.57 | 638.02 | 1.33 |
| United Kingdom | Cirrhosis | 4787.70 | | 9257.61 | 93.36 | 1.90 |
| United Kingdom | Cirrhosis due to hepatitis B | 302.77 | | 576.75 | 90.49 | 1.85 |
| United Kingdom | Cirrhosis due to hepatitis C | 412.88 | | 802.81 | 94.44 | 1.92 |
| United Kingdom | Cirrhosis due to alcohol use | 2081.59 | | 4103.73 | 97.14 | 1.97 |
| United Kingdom | Cirrhosis due to other causes | 1564.76 | | 2777.20 | 77.48 | 1.58 |
| United Kingdom | Cirrhosis due to NASH | 425.71 | | 997.13 | 134.23 | 2.61 |
| United States | Cirrhosis | 35809.57 | | 62493.46 | 74.52 | 1.14 |
| United States | Cirrhosis due to hepatitis B | 2460.67 | | 3582.37 | 45.59 | 0.47 |
| United States | Cirrhosis due to hepatitis C | 12607.34 | | 21851.19 | 73.32 | 1.12 |
| United States | Cirrhosis due to alcohol use | 10016.86 | | 17429.87 | 74.01 | 1.13 |
| United States | Cirrhosis due to other causes | 7539.50 | | 12850.23 | 70.44 | 1.06 |
| United States | Cirrhosis due to NASH | 3185.21 | | 6779.81 | 112.85 | 1.88 |
| Uruguay | Cirrhosis | 506.71 | | 495.27 | -2.26 | -0.40 |
| Uruguay | Cirrhosis due to hepatitis B | 78.06 | | 66.61 | -14.66 | -0.91 |
| Uruguay | Cirrhosis due to hepatitis C | 154.95 | | 150.43 | -2.92 | -0.43 |
| Uruguay | Cirrhosis due to alcohol use | 157.49 | | 146.70 | -6.85 | -0.58 |
| Uruguay | Cirrhosis due to other causes | 74.75 | | 81.12 | 8.53 | -0.02 |
| Uruguay | Cirrhosis due to NASH | 41.46 | | 50.41 | 21.59 | 0.41 |
| Uzbekistan | Cirrhosis | 4158.83 | | 10973.93 | 163.87 | 2.00 |
| Uzbekistan | Cirrhosis due to hepatitis B | 1016.08 | | 2452.22 | 141.34 | 1.67 |
| Uzbekistan | Cirrhosis due to hepatitis C | 936.30 | | 2608.92 | 178.64 | 2.20 |
| Uzbekistan | Cirrhosis due to alcohol use | 1371.69 | | 3905.74 | 184.74 | 2.28 |
| Uzbekistan | Cirrhosis due to other causes | 559.76 | | 1103.19 | 97.08 | 0.92 |
| Uzbekistan | Cirrhosis due to NASH | 275.00 | | 903.86 | 228.68 | 2.81 |
| Vanuatu | Cirrhosis | 33.32 | | 59.24 | 77.78 | -0.27 |
| Vanuatu | Cirrhosis due to hepatitis B | 16.77 | | 26.65 | 58.85 | -0.69 |
| Vanuatu | Cirrhosis due to hepatitis C | 7.28 | | 14.09 | 93.66 | 0.05 |
| Vanuatu | Cirrhosis due to alcohol use | 4.57 | | 8.82 | 92.95 | 0.03 |
| Vanuatu | Cirrhosis due to other causes | 2.63 | | 4.75 | 80.90 | -0.20 |
| Vanuatu | Cirrhosis due to NASH | 2.07 | | 4.93 | 137.95 | 0.81 |
| Venezuela | Cirrhosis | 2062.23 | | 3744.88 | 81.59 | 0.37 |
| Venezuela | Cirrhosis due to hepatitis B | 151.92 | | 256.47 | 68.82 | 0.10 |
| Venezuela | Cirrhosis due to hepatitis C | 602.66 | | 1106.97 | 83.68 | 0.42 |
| Venezuela | Cirrhosis due to alcohol use | 820.50 | | 1443.70 | 75.95 | 0.26 |
| Venezuela | Cirrhosis due to other causes | 211.20 | | 290.52 | 37.56 | -0.65 |
| Venezuela | Cirrhosis due to NASH | 275.95 | | 647.21 | 134.54 | 1.32 |
| Vietnam | Cirrhosis | 11566.27 | | 22607.36 | 95.46 | 1.20 |
| Vietnam | Cirrhosis due to hepatitis B | 4747.52 | | 8597.18 | 81.09 | 0.92 |
| Vietnam | Cirrhosis due to hepatitis C | 3111.53 | | 6407.38 | 105.92 | 1.39 |
| Vietnam | Cirrhosis due to alcohol use | 1816.05 | | 4230.20 | 132.93 | 1.85 |
| Vietnam | Cirrhosis due to other causes | 1013.23 | | 1566.46 | 54.60 | 0.33 |
| Vietnam | Cirrhosis due to NASH | 877.94 | | 1806.14 | 105.72 | 1.39 |
| Virgin Islands, U.S. | Cirrhosis | 19.75 | | 30.41 | 53.95 | 1.64 |
| Virgin Islands, U.S. | Cirrhosis due to hepatitis B | 2.34 | | 3.05 | 30.14 | 1.02 |
| Virgin Islands, U.S. | Cirrhosis due to hepatitis C | 4.33 | | 6.48 | 49.71 | 1.54 |
| Virgin Islands, U.S. | Cirrhosis due to alcohol use | 7.31 | | 11.13 | 52.24 | 1.60 |
| Virgin Islands, U.S. | Cirrhosis due to other causes | 2.17 | | 3.24 | 49.62 | 1.53 |
| Virgin Islands, U.S. | Cirrhosis due to NASH | 3.61 | | 6.51 | 80.56 | 2.23 |
| Yemen | Cirrhosis | 1020.62 | | 1708.47 | 67.39 | -1.04 |
| Yemen | Cirrhosis due to hepatitis B | 343.08 | | 535.13 | 55.98 | -1.30 |
| Yemen | Cirrhosis due to hepatitis C | 369.45 | | 661.65 | 79.09 | -0.79 |
| Yemen | Cirrhosis due to alcohol use | 58.71 | | 104.63 | 78.21 | -0.81 |
| Yemen | Cirrhosis due to other causes | 171.50 | | 247.30 | 44.20 | -1.60 |
| Yemen | Cirrhosis due to NASH | 77.88 | | 159.76 | 105.14 | -0.29 |
| Zambia | Cirrhosis | 2181.78 | | 3257.31 | 49.30 | -1.42 |
| Zambia | Cirrhosis due to hepatitis B | 581.85 | | 812.83 | 39.70 | -1.67 |
| Zambia | Cirrhosis due to hepatitis C | 648.35 | | 1001.42 | 54.46 | -1.30 |
| Zambia | Cirrhosis due to alcohol use | 421.22 | | 634.66 | 50.67 | -1.39 |
| Zambia | Cirrhosis due to other causes | 390.49 | | 582.27 | 49.12 | -1.43 |
| Zambia | Cirrhosis due to NASH | 139.87 | | 226.13 | 61.67 | -1.13 |
| Zimbabwe | Cirrhosis | 1122.78 | | 1439.68 | 28.22 | -0.38 |
| Zimbabwe | Cirrhosis due to hepatitis B | 320.68 | | 347.20 | 8.27 | -1.00 |
| Zimbabwe | Cirrhosis due to hepatitis C | 368.63 | | 500.20 | 35.69 | -0.17 |
| Zimbabwe | Cirrhosis due to alcohol use | 188.59 | | 248.10 | 31.56 | -0.28 |
| Zimbabwe | Cirrhosis due to other causes | 181.03 | | 245.86 | 35.81 | -0.16 |
| Zimbabwe | Cirrhosis due to NASH | 63.84 | | 98.32 | 54.00 | 0.30 |
